# Gut microbiomes from Gambian infants reveal the development of a non-industrialized *Prevotella*-based trophic network

**DOI:** 10.1038/s41564-021-01023-6

**Published:** 2021-12-31

**Authors:** Marcus C. de Goffau, Amadou T. Jallow, Chilel Sanyang, Andrew M. Prentice, Niamh Meagher, David J. Price, Peter A. Revill, Julian Parkhill, Dora I. A. Pereira, Josef Wagner

**Affiliations:** 1 Parasites and Microbes, Wellcome Sanger Institute, Cambridge, UK; 2 Department of Vascular Medicine, Academic Medical Center, University of Amsterdam, Amsterdam, The Netherlands; 3 Medical Research Council Unit The Gambia at the London School of Hygiene & Tropical Medicine, Banjul, The Gambia; 4 Department of Infectious Diseases at the Doherty Institute for Infection & Immunity, The University of Melbourne and Royal Melbourne Hospital, Melbourne, VIC Australia; 5 Centre for Epidemiology & Biostatistics, Melbourne School of Population & Global Health, The University of Melbourne, Melbourne, Australia; 6 Victorian Infectious Disease Reference Laboratory, the Peter Doherty Institute for Infection and Immunity, The University of Melbourne and Royal Melbourne Hospital, Melbourne, Australia; 7 Department of Veterinary Medicine, University of Cambridge, Cambridge, UK; 8 Department of Pathology, University of Cambridge, Cambridge, UK

**Keywords:** Microbial ecology, Microbiome

## Abstract

Distinct bacterial trophic networks exist in the gut microbiota of individuals in industrialized and non-industrialized countries. In particular, non-industrialized gut microbiomes tend to be enriched with *Prevotella* species. To study the development of these *Prevotella*-rich compositions, we investigated the gut microbiota of children aged between 7 and 37 months living in rural Gambia (616 children, 1,389 stool samples, stratified by 3-month age groups). These infants, who typically eat a high-fibre, low-protein diet, were part of a double-blind, randomized iron intervention trial (NCT02941081) and here we report the secondary outcome. We found that child age was the largest discriminating factor between samples and that anthropometric indices (collection time points, season, geographic collection site, and iron supplementation) did not significantly influence the gut microbiome. *Prevotella copri*, *Faecalibacterium prausnitzii* and *Prevotella stercorea* were, on average, the most abundant species in these 1,389 samples (35%, 11% and 7%, respectively). Distinct bacterial trophic network clusters were identified, centred around either *P.* *stercorea* or *F.* *prausnitzii* and were found to develop steadily with age, whereas *P.* *copri*, independently of other species, rapidly became dominant after weaning. This dataset, set within a critical gut microbial developmental time frame, provides insights into the development of *Prevotella*-rich gut microbiomes, which are typically understudied and are underrepresented in western populations.

## Abstract (web summary)

Analysis of gut microbiomes from Gambian infants reveals bacterial trophic networks based around *Prevotella* species, which are typically enriched in non-industrialized populations.

## Main

Development of the gut microbiome in infants (aged 0 to 1 year) and toddlers (aged 1–3 years) has been an area of intense research but has mainly focused on European and US populations. Development of the infant gut microbiota in pre-industrial populations is therefore of interest. Several important studies have shown that pre-industrial microbiotas are dominated by *Prevotella*, a genus whose abundance is much lower in industrial and westernized nations where *Bacteroides* is typically much more dominant. A well-known example of this is the study by Yatsunenko et al. in which they compared healthy Amerindians from the Amazonas of Venezuela, residents of rural Malawian communities and inhabitants of metropolitan areas in the USA^1^. This study found that the most distinguishing difference between Americans and either pre-industrial group was the *Prevotella*/*Bacteroides* ratio. The same was also found when comparing children in West Africa (Burkina Faso) and Europe (Italy)^2^. Other studies from Malawi^3^ and Nigeria^4^ studying rural populations similarly confirmed the high abundance of *Prevotella*.

An interesting concept within the human gut microbiome is the enterotype hypothesis which clusters the gut microbiome compositions of individuals, from a simplified point of view, into those dominated by *Bacteroides*, Ruminococcaceae or *Prevotella*^5,6^. In industrialized countries, the *Bacteroides* and Ruminococcaceae enterotypes are typically common, whereas the *Prevotella* enterotype is more commonly detected in countries with pre-industrialized lifestyles^7,8^ or in people with a more plant-based diet (vegetarians). Although the concept of enterotypes remains controversial, most agree that the abundance of *Prevotella* is one of the most discriminative factors when comparing various microbial compositions.

In the current study, we utilized data from an iron intervention trial in The Gambia, West Africa^9^. Consisting of 616 children, this is the largest paediatric cohort studied thus far in a critical time window for gut microbiome development (7 to 37 months) in a non-industrialized environment. This exploratory study provides important insights into the development of trophic networks in individuals whose gut microbiome is dominated by *Prevotella*. Bacterial trophic networks can be understood as microbial populations that form clusters and, at the trophic level, constitute a food web of metabolically interdependent organisms^10,11^. To date, such analyses have been performed mostly on *Prevotella*-poor cohorts from industrialized countries using combinations of adults and children, or to some degree, on smaller paediatric groups in non-industrialized countries^12^. Information about the study setting for the iron intervention trial and additional nutritional and dietary information can be found in the Methods.

## Results

### Sampling framework and sample characteristic

We performed 16S ribosomal RNA amplification and sequencing on a total of 1,546 faecal samples from a secondary study outcome as a part of a double-blind, randomized iron intervention trial (NCT02941081) in The Gambia. A flow diagram (Supplementary Information) shows the samples taken for the secondary study outcome.

After quality filtering and applying exclusion criteria, 1,389 samples remained. A detailed description of the participants, exclusion criteria and sampling framework is available in the Methods. Children were enroled between the ages of 7 and 37 months, and samples were collected at three different time points (day 1, day 15 and day 85) during an iron intervention trial in The Gambia^9^ (see Extended Data Table 1 for additional details). The children were initially split into three age groups (7 to <12 months, 12 to <24 months and 24 to 37 months at the time of enrolment) and subsequently analysed in detail in 11 age groups.

### Justification for combining treatment and placebo groups

Multivariate and univariate analyses were conducted to identify whether children from the treatment arm (iron supplementation) and from the placebo group could be analysed together. Multidimensional scaling using principal coordinates analysis (PCoA) did not cluster children differently based on treatment at the individual time point (Supplementary Fig. 1a–c) or in the combined time point analysis (Supplementary Fig. 1a–d). Volcano plot analysis identified one species that was statistically different between the two sample groups (red dot in Supplementary Fig. 1e). The higher abundance in the treatment group of this single species, *Megamonas funiformis*, was subsequently confirmed by the Kruskal–Wallis rank test with a false discovery rate (FDR) corrected *P* value of 0.01 (Supplementary Table 1). ‘ALDEx2’ did not identify any other taxa as statistically significantly different (Supplementary Table 1). Because the bacterial compositions between both groups were highly similar, as is also shown in subsequent sections in regards to other aspects such as diversity, we subsequently analysed all samples together as one group. Thus, although this gut microbiome dataset does not inform on the controlled trial aspects of the original study, it does represent an excellent opportunity for understanding gut microbiome development over time in these African children.

### Alpha-diversity increase over time from 7 to 40 months of age

**Extended Data Fig. 1:**
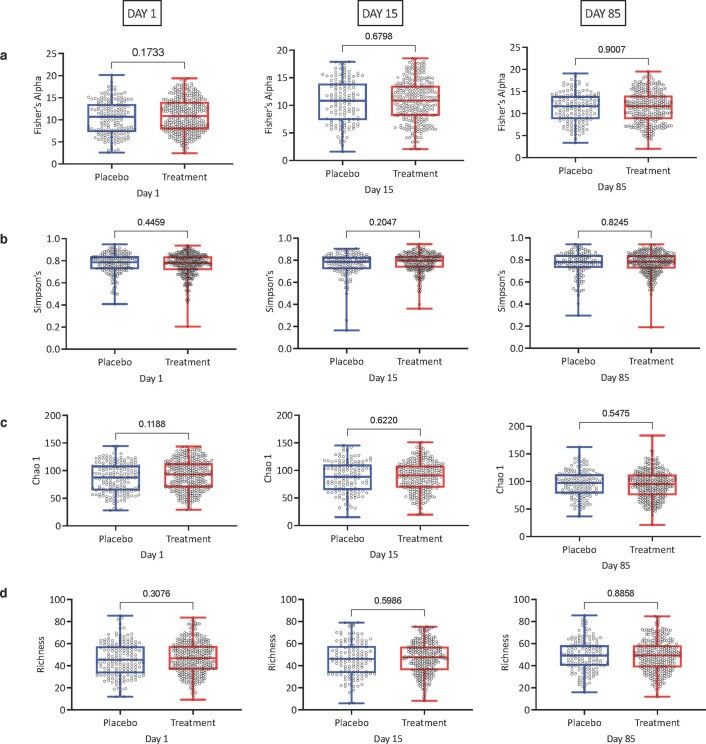
Alpha diversity indexes did not differ between placebo and treatment groups at the three individual sampling timepoints. The Fisher’s alpha diversity index (**row a**), the Simpson’s diversity index (**row b**), the Chao1 estimated richness (**row c**), and the observed richness (**row d**) did not differ between the placebo and treatment group at the three individual sampling timepoints DAY 1, DAY 15, and DAY 85. Data were tested for normality using the Anderson-Darling and the D’Agostino & Pearson test in Prism 9 for macOS. For Day 1 and Day 15 samples, the Fisher’s Alpha index, the Simpson’s index, and the Chao 1 index data were not normally distributed, whereas the Richness index data were normally distributed. For Day 85 samples the Fisher’s Alpha index and the Simpson’s index data were not normally distributed, whereas the Chao 1 index and the Richness index data were normally distributed. Not normally distributed data were tested with the two-tailed unpaired non-parametric Mann-Whitney test and normally distributed were tested with the two-tailed unpaired parametric t test. Data were considered to be statistically significant with a confidence level of 95%. Data were plotted using the Box and whiskers plot function in Prism 9 for macOS and whiskers showing all point from minimum to maximum values. The box always extends from the 25th to 75th percentiles. The line in the middle of the box is plotted at the median. In the day 1 dataset there were 520 samples, in the day 15 dataset there were 412 samples, in the day 85 dataset there were 457 samples.

**Extended Data Fig. 2:**
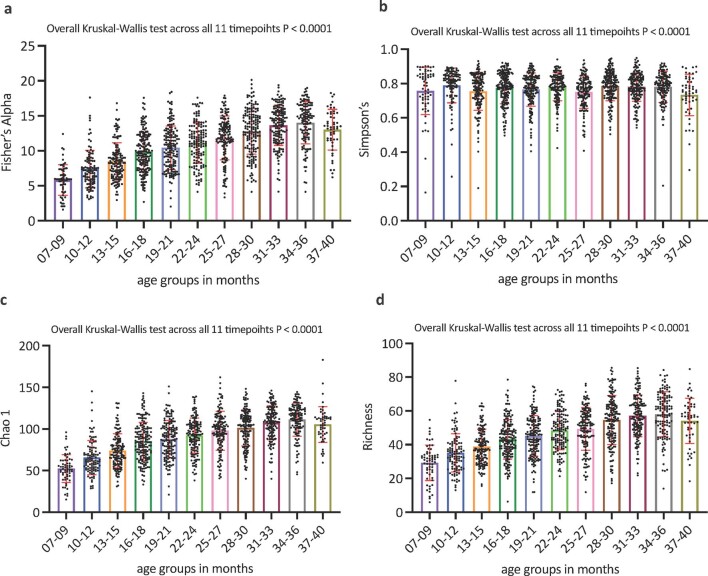
Alpha diversity indexes differs between the 11 3-month age groups interval datasets in the combined sampling timepoints data. The Fisher’s alpha diversity index (**a**), the Simpson’s diversity index (**b**), the Chao1 estimated richness (**c**), and the observed richness **(d**) did differ significantly across the 11 3-month age groups as analysed by the non-parametric Kruskal-Wallis test. Data were tested for normality using the Anderson-Darling and the D’Agostino & Pearson test in Prism 9 for macOS and were non-normally distributed. Data were considered to be statistically significant with a confidence level of 95%. Data were plotted using the Scatter dot plot function in Prism 9 for macOS with a line drawn at the mean with error bars extending to the standard deviation. In this overall general presentation of the alpha diversity indexes, all 1389 samples across the three sampling timepoints were combined. The same analysis was performed for the three different individual sampling timepoints Day 1, Day 15, and Day 85 which is shown in Extended Data Fig. 3.

**Extended Data Fig. 3:**
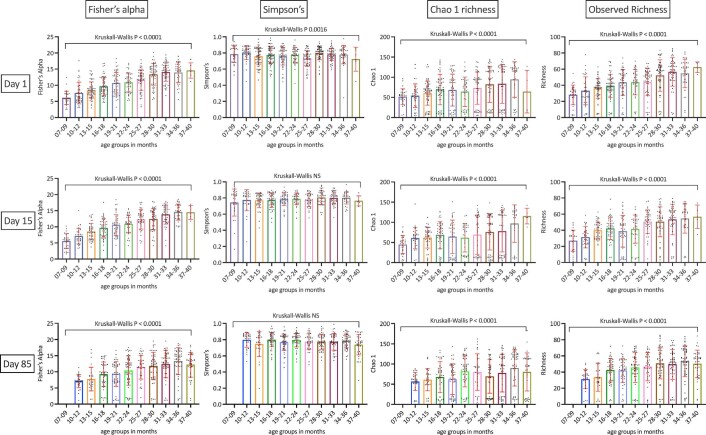
Alpha diversity indexes stratified by the three sampling time points (Day 1, Day 15, and Day 85). The Fisher’s alpha diversity index, the Chao1 estimated richness, and the observed richness were significantly different across the 11 3-month age groups as analysed by the non-parametric Kruskal-Wallis test. Data were tested for normality using the Anderson-Darling and the D’Agostino & Pearson test in Prism 9 for macOS and were non-normally distributed. The Simpson’s index was only significantly different in the day 1 dataset. Data were considered to be statistically significant with a confidence level of 95%. Data were plotted using the Scatter dot plot function in Prism 9 for macOS with a line drawn at the mean with error bars extending to the standard deviation. In the day 1 dataset there were 520 samples, in the day 15 dataset there were 412 samples, in the day 85 dataset there were 457 samples.

The alpha-diversity indexes for Fisher’s alpha parameter, Simpson’s index, Chao1 richness index and Richness index (observed richness) were not significantly different between the treatment and placebo group (Extended Data Fig. 1). To follow alpha-diversity changes over time in the whole data set, we split the data into 11 age groups, separated by 3-month intervals based on the age group at sampling. Fisher’s alpha parameter indicated a statistically significant increase in alpha-diversity from the youngest age group (7–9 months) to the oldest (37–40 months) age group (Kruskal–Wallis *P* < 0.0001) and a gradual increase in between (Extended Data Fig. 2). Individual time point analysis also highlights the higher robustness of Fisher’s alpha index (Extended Data Fig. 3). Additional information about other alpha-diversity indexes are presented in the Supplementary Information (alpha- and beta-diversity sections).

### Beta-diversity differs between age groups

**Extended Data Fig. 4:**
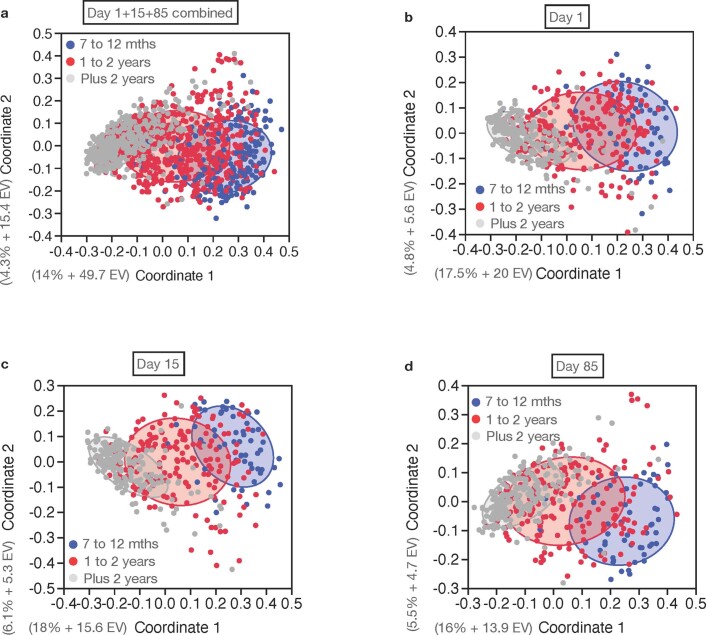
PCoA analysis for the three-age group comparison shows distinctive microbiome clusters. PCoA based on Bray-Curtis distance matrix performed on three age group categories showed that the structure of bacterial communities differs between the young (7 to 12 mths), the middle (1 to 2 years), and the old (plus 2 years) age groups for the combined time-point dataset (**a**), for the day 1 samples (**b**), for the day 15 samples (**c**), and for the day 85 samples (**d**). The explained variance and the Eigenvalue (EV) for the coordinates 1 and 2 are shown in brackets at the left hand size of the Coordinate axis label.

**Extended Data Fig. 5:**
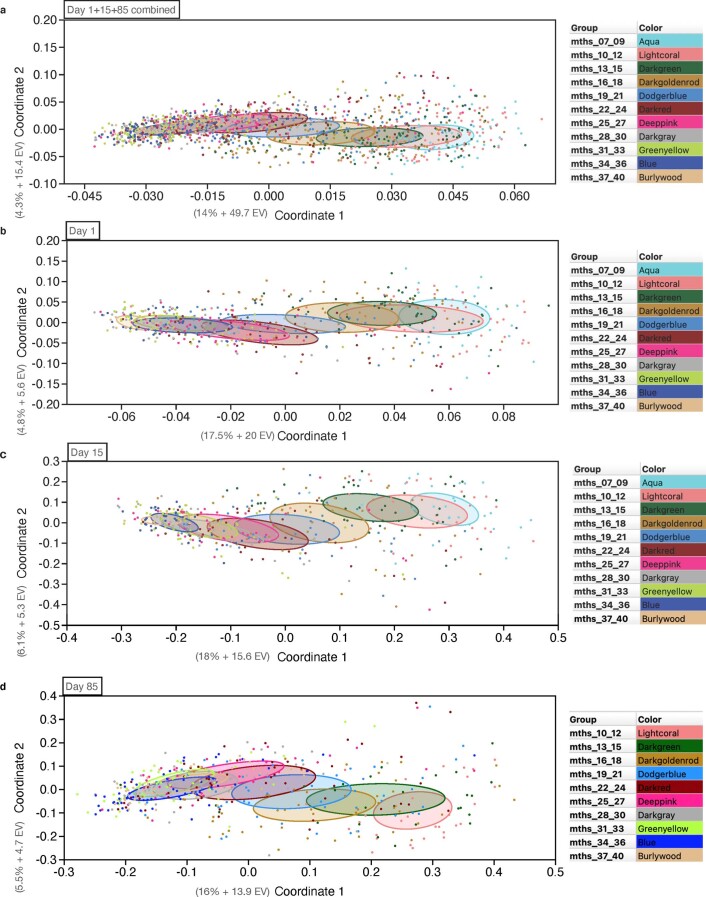
PCoA analysis for the 11-age group comparison shows distinctive bacterial clusters. PCoA based on Bray-Curtis distance matrix performed on 11 3-months age group categories showed that the structure of bacterial communities differs between the age groups for the combined time point dataset (**a**), for the day 1 samples (**b**), for the day 15 samples (**c**), and for the day 85 samples (**d**). The explained variance and the Eigenvalue (EV) for the coordinates 1 and 2 are shown in brackets at the left hand size of the Coordinate axis label. The colour code at the right hand side of each PCoA plot illustrates the different 3-months age groups.

The PCoA of the gut microbiome stratified into three age group (7–12 months, 1–2 years and 2+ years, age taken at time of sampling) showed distinctive clusters with the youngest and oldest groups separated most from each other (Extended Data Fig. 4a–d). The Bonferroni corrected *P* value from the permutational multivariate analysis of variance (PERMANOVA) test and analysis of similarities (ANOSIM) test between the three different age groups was 0.0003 (Extended Data Table 2). Additional PCoA and PERMANOVA and ANOSIM tests for the 11 age group comparisons (Extended Data Fig. 5a–d), gender, geographic locations and season are presented in the Supplementary Information (alpha- and beta-diversity sections).

### Multivariable analysis to identify taxa associated with age, season and iron treatment

The R package MaAsLin2 was used to find taxa significantly associated with age, season (wet or dry) and treatment group. In the combined time point analysis, the individual subject identifier was used as a random effect to account for repeat sampling of participants. We also analysed the three individual time points separately. All statistically significant taxa (*q* value <0.05) are shown in Supplementary Table 5. The top associated taxa (coloured in Supplementary Table 5) with a minimum abundance of 0.2% are further discussed below.

**Fig. 1:**
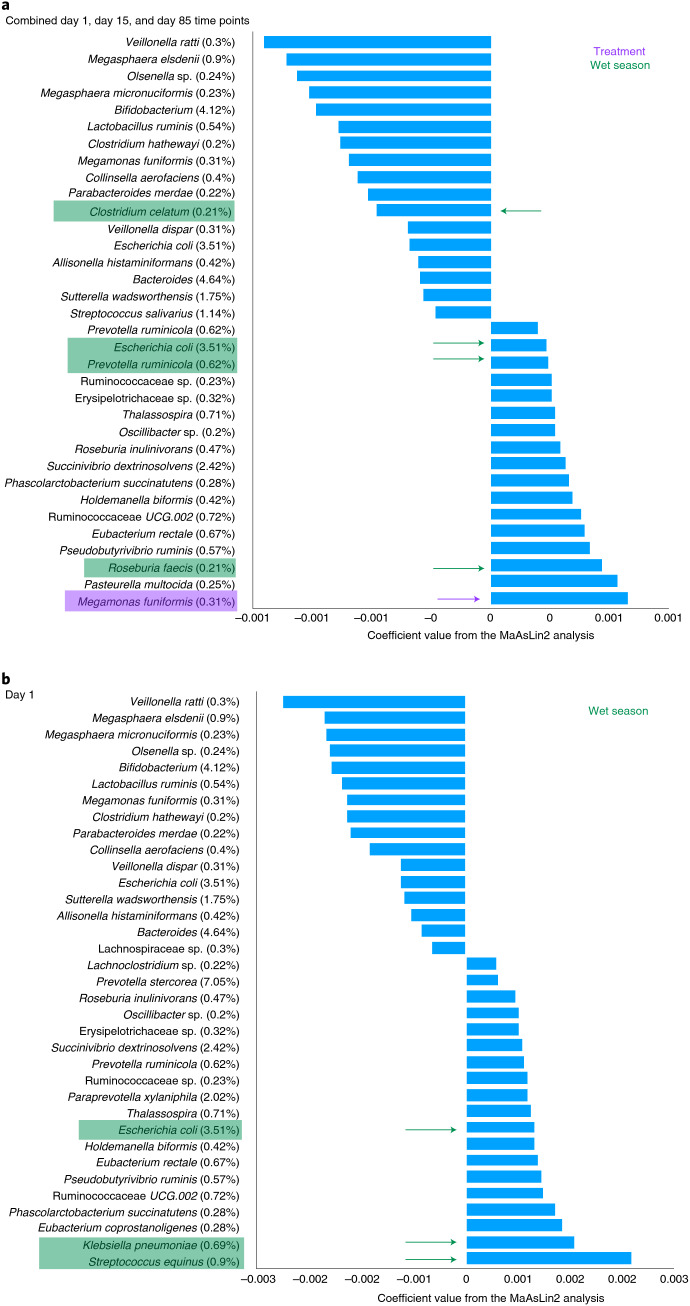
Multivariable statistical analysis to identify taxa associated with age, season and treatment group. **a**,**b** Taxa with a minimum abundance of 0.2% and FDR-corrected *P* value < 0.5 identified through the statistical MaAsLin2 R package in (**a**) the combined time point dataset and (**b**) the day 1 dataset. Taxa associated with treatment and season are highlighted in purple and green, respectively. Coefficient values shown on the *x* axis are taken from Supplementary Table 2. Values in parentheses next to the taxa name represent the relative abundance of the species.

In the combined time point analysis, 16 taxa were negatively associated with increasing age including *Bifidobacterium*, *Bacteroides*, *Escherichia coli*, *Sutterella wadsworthensis* and *Streptococcus salivarius*, which were all taxa in the top ten most abundant species with a minimum abundance of 1%. Thirteen taxa were positively associated with age, with *Succinivibrio dextrinosolvens, Ruminococcaceae* *UCG.002*, *Thalassospira*, *Eubacterium rectale* and *Prevotella ruminicola* being the most positively associated (Fig. 1a). The combined time point analysis also confirmed that *Megamonas funiformis* was associated with iron supplementation (purple arrow in Fig. 1a) yet this association disappeared when analysing day 1, day 15 or day 85 samples separately (Supplementary Table 5). One species (*Clostridium celatum*) was negatively and three species (*P. ruminicola*, *E. coli* and *Roseburia faecis*) were positively associated with the wet season (green arrows in Fig. 1a). In the day 15 and day 85 cohorts no taxa with a minimum abundance of 0.2% were associated with season or treatment (Supplementary Table 5), whereas in the day 1 cohort three taxa were associated with the wet season including *E. coli*, *Streptococcus equinus* and *Klebsiella pneumoniae* (Fig. 1b).

### Taxonomic differences between the young, middle and old age groups

To account for the association between age groups and time point, a mixed-effects linear regression model was utilized. These analyses were restricted to the top 50 taxa with a minimum abundance of 0.2% (91% of all 16S rRNA reads). Importantly, no adjustments for multiple comparisons were made to the 95% confidence intervals. First, the effect of time point on total sum scaling transformed and cumulative sum scaling) log_2_ normalized data (TSS + CSS) was assessed using a model that adjusted for season, site and age at enrolment. Repeated sampling was included as a random effect. These results indicated that no species showed notable changes at day 15 or day 85 when compared with day 1, either by including the three age-group variables (Supplementary Fig. 4) or by including the eleven 3-month age group variables (Supplementary Fig. 5).

The largest mean changes (just over one unit of TSS + CSS log_2_ data), were negligible in comparison with the changes between age groups (results below).

**Extended Data Fig. 6:**
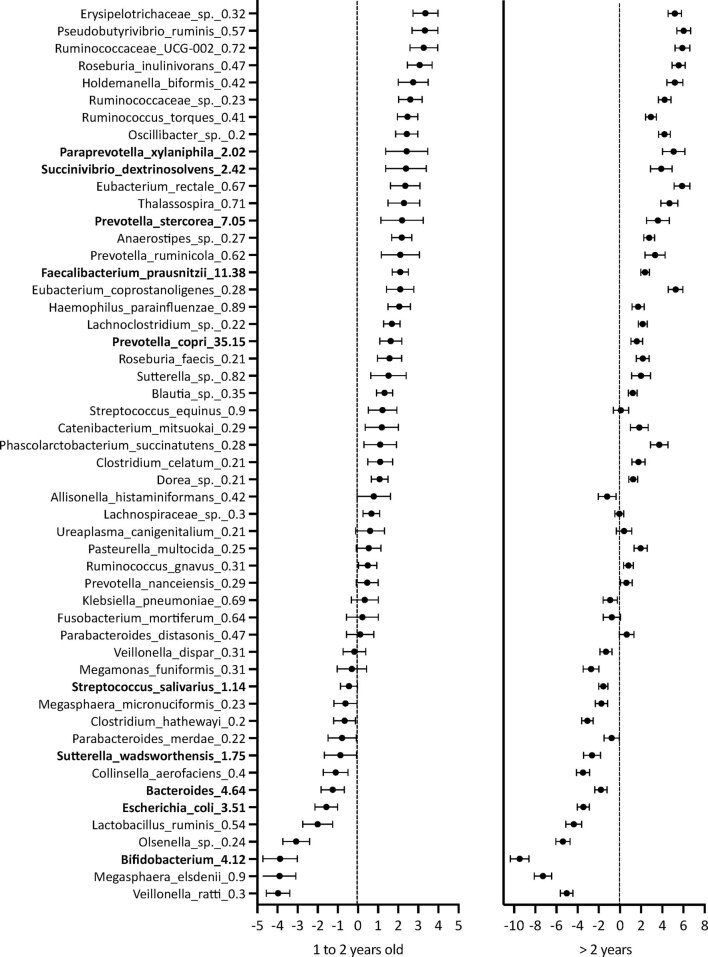
Mixed-effect linear regression to show bacterial changes across the three different age groups. Mixed-effect linear regression was conducted to examining the effect of the three different age groups. The mean changes (95% CI) in TSS + CSSlog2 transformed + normalized data of one to two years old samples and >2-year-old samples compared to seven months to 12 months samples are shown. The analysis was adjusted for season, site (fixed effect) and child ID (multiple sampling = random effect). The taxa are sorted from largest negative mean change to largest positive mean change on the x axis. The top ten taxa with a minimum abundance of 1% high highlighted in bold. Mixed-effects linear regression analysis was restricted to the top 50 taxa with a minimum abundance of 0.2%. Importantly, no adjustments for multiple comparisons were made to the 95% confidence intervals. Subject identifier (child ID) was included as a random-effect to account for repeated measures. In this analysis, all 1389 samples across the three sampling timepoints were combined. The black points shows the estimated change and the error bars show the 95% confidence interval.

After establishing that time point had a minimal effect on abundances, we also estimated the effect of age group at sampling using all available data, while simultaneously adjusting for season and site (geographic location) with repeated sampling included as a random effect. The mean changes for all 50 taxa were between −4 and 3.4 unit of TSS + CSS log_2_ data for the 1–2 years middle age group compared with the 7–12 months young age group, and between −9.5 and 6 for the >2 years old age group compared with the 7–12 months age group (Extended Data Fig. 6).

**Fig. 2:**
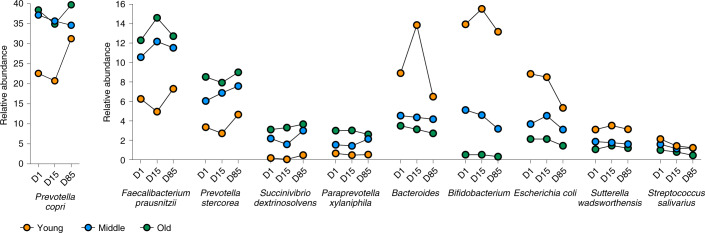
Top ten taxa with a minimum abundance of 1% across the young, middle and old groups. Top ten taxa with a minimum abundance of 1% identified through mixed-effect linear regression associated with the three age groups stratified by the three sampling time point. Relative abundances (%) are plotted on the *y* axis and the taxa across the three different sampling time points are plotted on the *x* axis. D, day.

A detailed presentation of the top ten taxa with a minimum abundance of 1% showing changes between day 1, day 15 and day 85 in the young, middle and old age groups are shown in Fig. 2. Of the top ten taxa, five increased in abundance over time including the top three most abundant taxa, *Prevotella* *copri* (35.2%), *Faecalibacterium prausnitzii* (11.4%) and *Prevotella* *stercorea* (7.1%), as well as *Succinivibrio dextrinosolvens* and *Paraprevotella xylaniphila*. Five taxa decreased over time including the next three most abundant, *Bacteroides* (4.6%), *Bifidobacterium* (4.1%) and *E. coli* (3.5%) as well as *Sutterella wadsworthensis* and *Streptococcus salivarius*.

### Visualization of the maturation of the gut microbiome

**Fig. 3:**
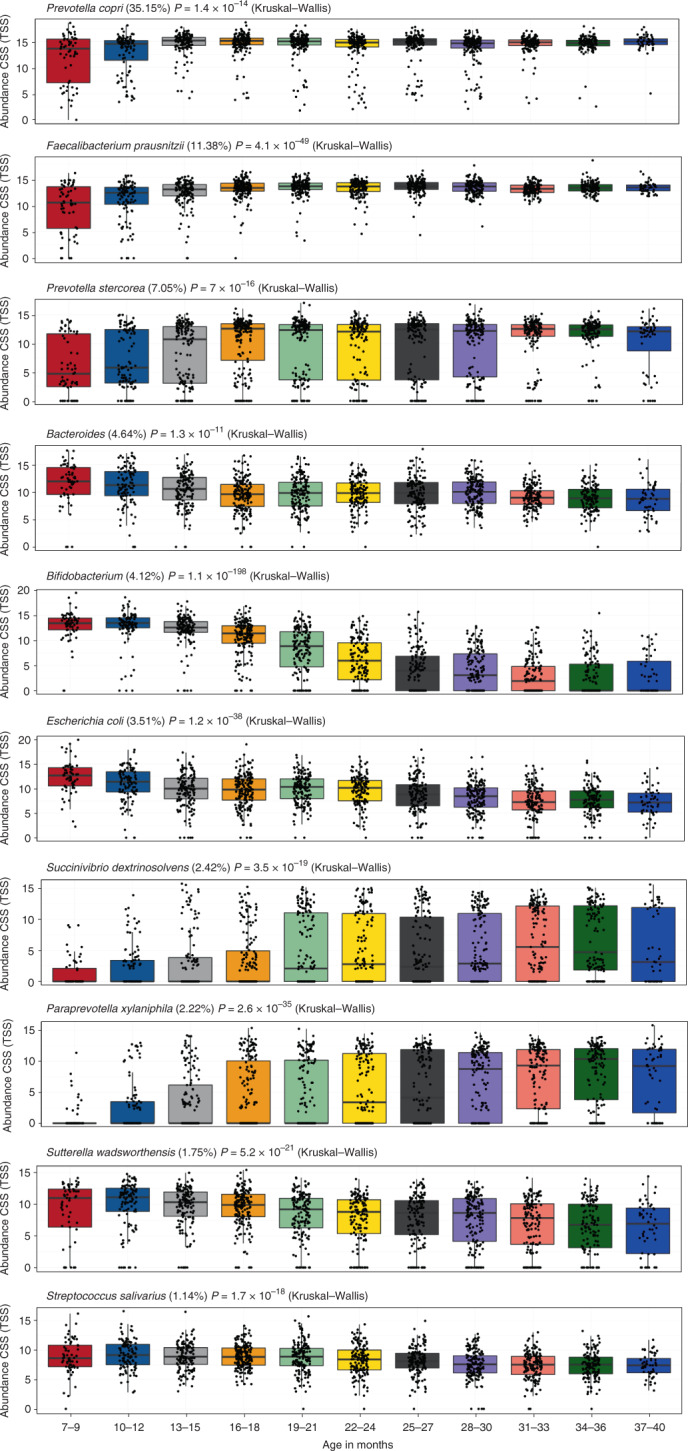
Top ten species with a minimum abundance of 1%, significantly associated in the 11 age groups. Significantly differentially abundant bacterial taxa on the TSS + CSS log2 data. The box plot shows the minimum, first quartile, median, third quartile and maximum values. The *P* value from the Kruskal–Wallis test shows the overall significance across all eleven 3-month age groups. In this overall general presentation, all three sampling time points were combined.

Scatterplots of the ten most abundant taxa across the 11 3-month age groups visualize how all of these taxa either increase or decrease significantly in abundance over time (Fig. 3). Five of these taxa (58% of all reads) increased significantly over time, including *P. copri* (35.2%), *F. prausnitzii* (11.4%), *P. stercorea* (7.1%), *Succinivibrio dextrinosolvens* (2.4%) and *Paraprevotella xylaniphila* (2%). The other five taxa (15% of all 16S reads) decreased significantly over time, including *Bacteroides* (4.6%), *Bifidobacterium* (4.1%), *E. coli* (3.5%), *Sutterella wadsworthensis* (1.8%) and *Streptococcus salivarius* (1.1%). *Prevotella copri*, the most abundant bacterium, reached a stable level shortly after 1 year. *Faecalibacterium* *prausnitzii*, the second most abundant bacterium, reached a stable level a few months later. The most significant decrease in abundance was observed for *Bifidobacterium*, which assumedly had already begun to decrease in abundance before the age of 7 months as a result of weaning, but continued to decrease rapidly reaching a steady low state at approximately 2 years.

**Fig. 4:**
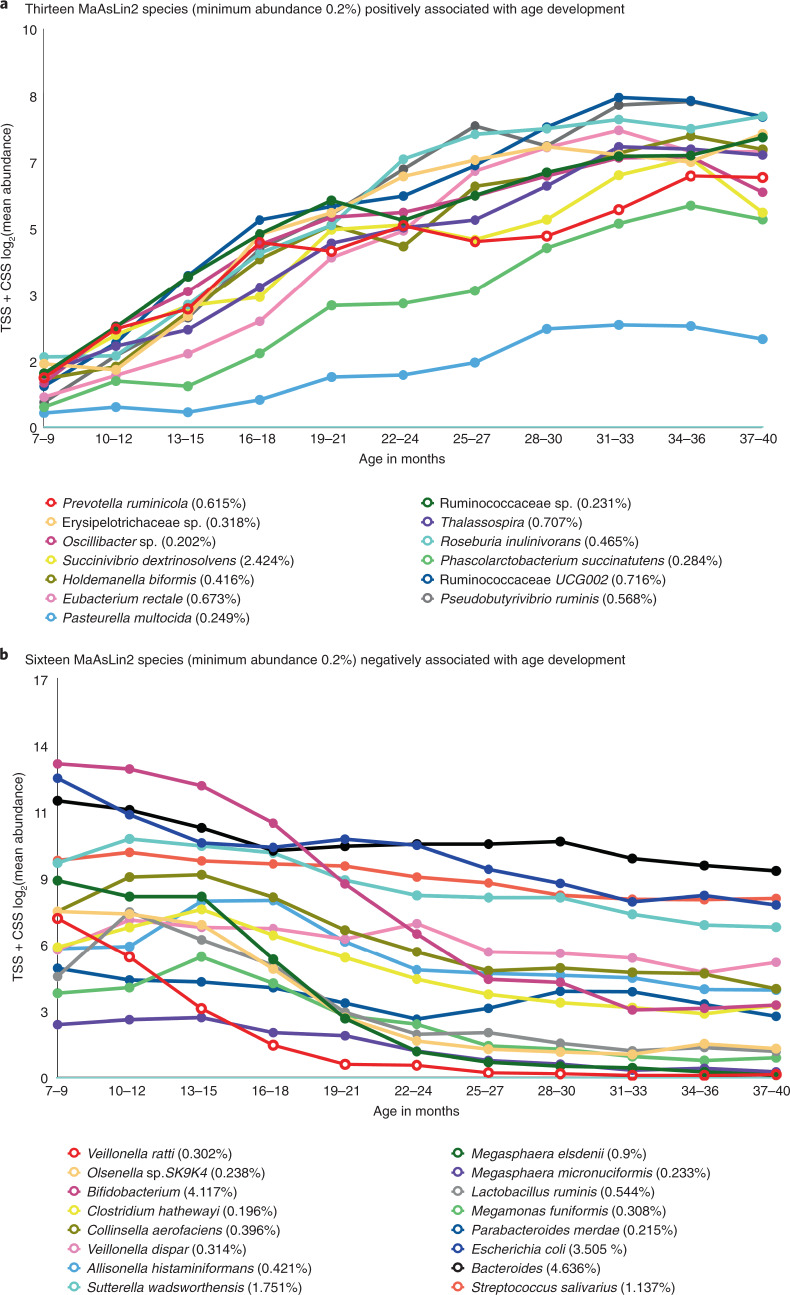
Bacterial taxa significantly associated with age development in the combined time points dataset. **a**,**b**, Taxa identified through the MaAsLin2 R package analysis that are statistically associated with age (Supplementary Table 2) were plotted in a stacked line chart for the eleven 3-month age group intervals, separated by (**a**) positive association with age maturation and (**b**) negative association with age maturation. Only taxa with a minimum abundance of 0.2% were plotted. Absolute abundant bacterial 16S reads transformed using TSS and normalized using CSS + log_2_ are shown on the *y* axis.

In a second analysis, we plotted the bacterial taxa most strongly associated with age according to MaAsLin2 analysis (Supplementary Table 5) with a minimum abundance of 0.2%, stratified by the eleven 3-month age groups. In the combined analysis, the individual sampling time points were used as a random effect (Fig. 4). Strikingly, many of the taxa positively associated with age (Fig. 4a) increase steadily in number at similar rates, at least during the first 2 years of life, whereas the rates of decrease in those species negatively associated with age (Fig. 4b) appear less concordant.

### Correlation analysis identifies distinct bacterial trophic networks

**Fig. 5:**
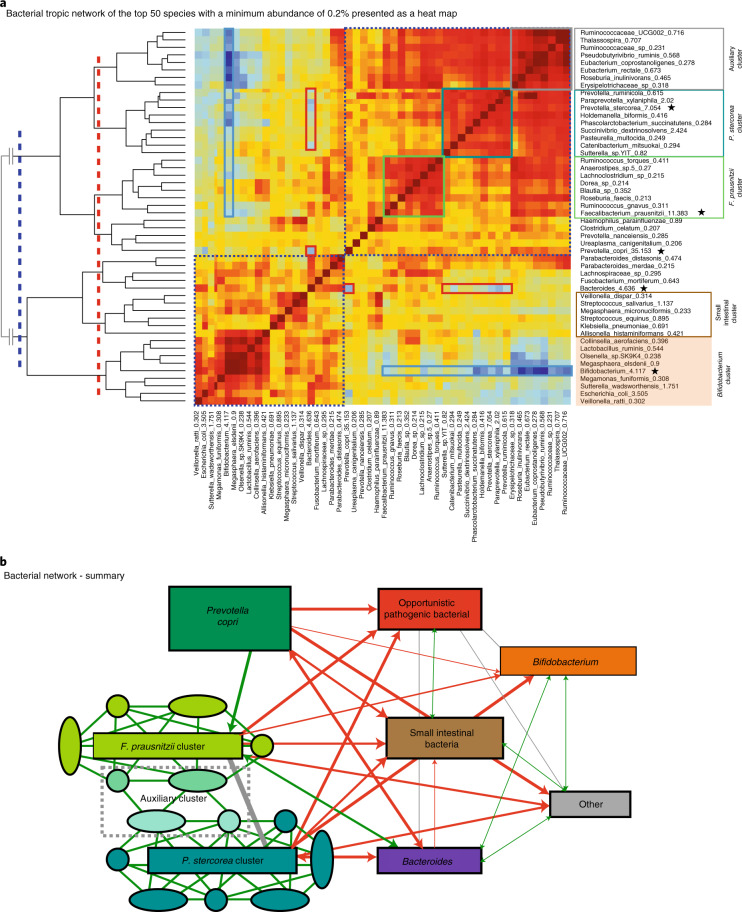
Taxa association and trophic networks with all three time points combined. **a**, A network heatmap was generated using the top 50 taxa with a minimum abundance of 0.2% in all samples. The most dominant clusters identified in the bacterial trophic network correlation analysis are highlighted by different coloured boxes and were confirmed by gap statistics. Two different settings in the gap statistical analysis identified two (blue dotted line) and seven clusters (red dotted line), respectively. The *P. stercorea* network is highlighted in teal, the *F. prausnitzii* network in light green, the *Bifidobacterium* network in yellow, an auxiliary group in grey and a small intestinal microbiome network in brown. The red heatmap colour indicates a strong positive correlation and the blue heatmap colour indicates a strong negative correlation. Red boxes denote a negative association between *Prevotella* species and *Bacteroides* and blue boxes denote a negative association between *Bifidobacterium* and other taxa. The main representative taxa for each cluster are marked by a star. **b**, Bacterial network summary. Spearman’s rho correlation coefficient analysis was used to identify a bacterial trophic network with the strongest self-correlations. The leading taxa in each network is highlighted. A positive correlation is highlighted by green lines, a negative correlation by red lines and an intermediate correlation by grey lines.

**Extended Data Fig. 7:**
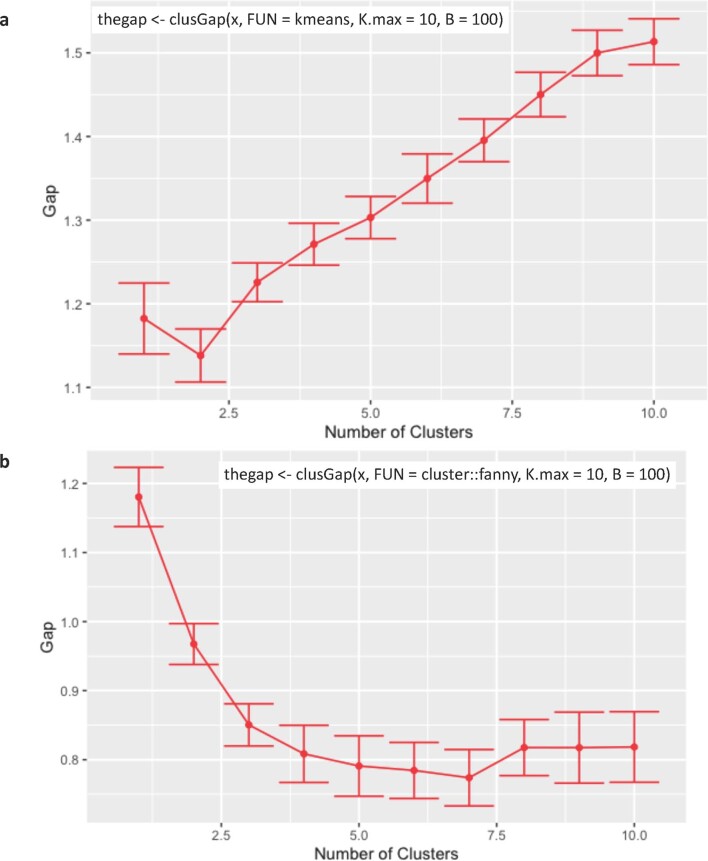
Using Gap Statistic to identify the best number of clusters for bacterial trophic network analysis. Gap statistic using the R function “clusGap” from the R package “cluster” version 2.1.2 was used to calculate a goodness of clustering measure, the “gap” statistic. The “k.max” parameter was set to 10, the bootstrap “B” parameter was set to 100, and the analysis was done with two different “FUNcluster” method including cluster:fanny and kmeans. The analysis was restricted to the top 50 TSS transformed taxa with a minimum abundance of 0.2%. **a**. The analysis using the “FUNcluster” method “kmeans”. **b**. the analysis using the “cluster::fanny”. The numbers of statistical identified clusters are characterized by a decrease in Gap value on the Y axis. Once there is not further decrease in the Gap value (line started to flattened out) on the y axis, indicates the optimal number of clusters. For this analysis all 1389 samples across the three sampling timepoints were combined. The error bars indicate one standard error.

Clusters of co-associated bacteria can be caused purely by temporal or, for example, medication-induced patterns, depending on the type of dataset, but they can also reflect food webs of metabolically interdependent organisms^10,11^. To identify these trophic networks and other clusters, we generated a heatmap of the Spearman’s rho correlation coefficients between the different top 50 taxa with a minimum abundance of 0.2% in all samples (Fig. 5a). For the overall presentation, we included samples from all time points and identified two main clusters according to the dendrogram on the left-hand side of the heatmap, indicated by a blue dotted line and blue dotted boxes. These two main clusters represent taxa that either increased in abundance over time (upper part, *P. copri* and everything above it) or those that decrease in abundance (lower part). Within the two main clusters seven subclusters can be further visualized, indicated by a red dotted line. These seven clusters were further confirmed by Gap Statistic analysis with a different ‘FUNcluster’ parameter in the analysis (Extended Data Fig. 7b).

**Fig. 6:**
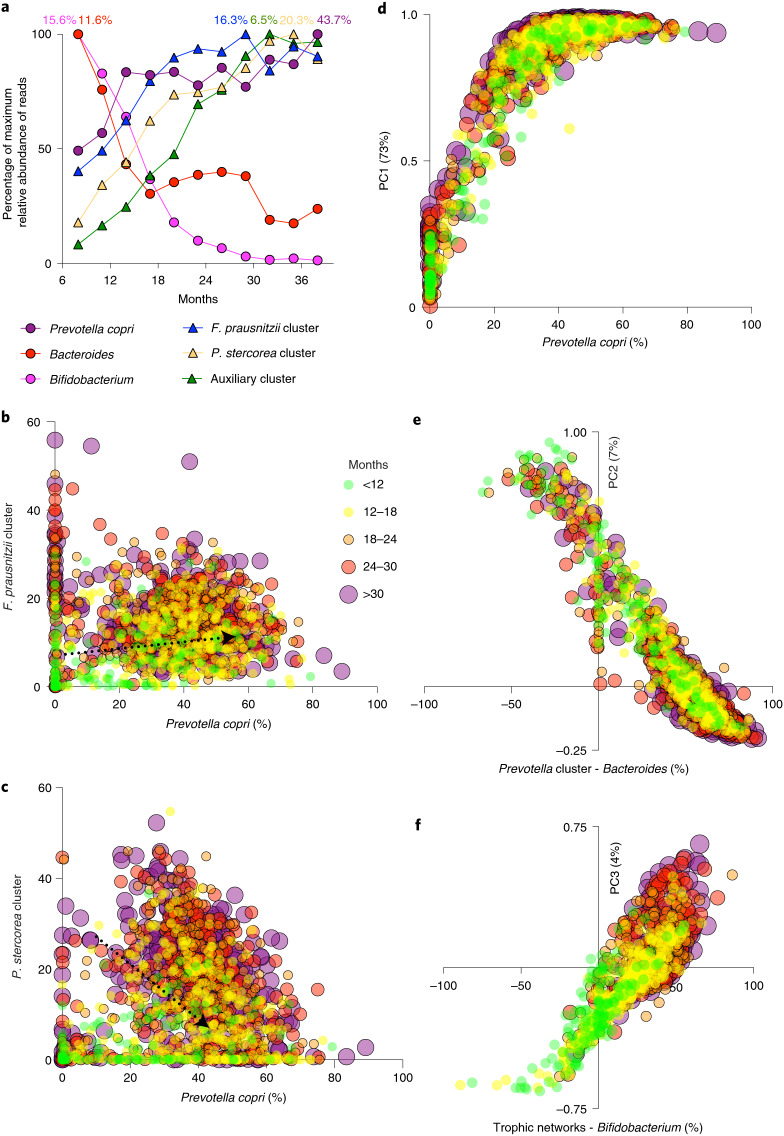
Maturation process analysed by correlation and principal component analysis. **a**, Abundances over time of the most relevant bacterial clusters depicted in Figs. 3 and 5 as important separate species/genera. Each bacterial group is normalized by its highest abundance at any time point. The highest abundance of each group (100% on the *y* axis) is depicted at the top as a percentage. *Bifidobacterium* and *Bacteroides* decrease over time, whereas the other groups increase over time but at different rates. **b**,**c**, Scatterplots of *P. copri* and the *F. prausnitzii* cluster (**b**) or the *P. stercorea* cluster (**c**). Because of the extremely high abundance of *P. copri* a numerically induced (weak) negative association is to be expected even between other clusters where no antagonistic interaction occurs, like with the *P. stercorea* cluster. The *F. prausnitzii* cluster, however, has a weak positive correlation with *P. copri*. **d**–**f**, Bacterial clusters and main species represent the main principal components within this dataset. **d**, PC1 is nearly fully described by the abundance of *P. copri*, the most abundant species at every point in time in this study. **e**, PC2 represents the antagonistic interaction between the *Prevotella* genus and the *Bacteroides* genus, where *Prevotella* is represented by the combined abundance of *P. copri* and the *P. stercorea* cluster. **f**, PC3 represents a shift away from a gut dominated by *Bifidobacterium*, as seen in young infants, towards the development of complex trophic networks as represented here by the combined abundance of the *F. prausnitzii* cluster, the *P. stercorea* cluster and the auxiliary cluster. PC3 is most strongly correlated with age. Percentages indicate the per cent variation explained by each principal component in the combined dataset.

The largest subcluster identified (teal box) encompassing 9 of the top 50 taxa, includes *P. stercorea* (7%) and consists of taxa generally not found in (high abundance) in the microbiota of individuals from industrialized countries. This cluster probably represents a trophic network of interdependent species that together increase steadily over time during the first 18 months and slowly afterwards reaching a maximum abundance just before 3 years (Fig. 6a). The second largest subcluster (green box) contains many taxa that are commonly also found (in high abundance) in industrialized microbiome compositions and includes the second most abundant species, *F. prausnitzii* (11%). This cluster takes only about 18 months to mature and was hence not represented in the MaAsLin2 age analysis, just like *P. copri*. The third most distinctive cluster (grey box) encompasses eight species that are positively correlated with each other and also with the *P. stercorea* and the *F. prausnitzii* cluster. This ‘shared auxiliary’ cluster is also seen to be increasing steadily during the first 2.5 to 3 years of life.

The clusters below the blue line in the middle of the heatmap are less coherent because they are mainly positively correlated with one another as they all decrease in abundance over time. In one of these subclusters, indicated with a brown box, various small intestinal species like *Veillonella dispar* and *Streptococcus salivarius* can be recognized. The other subcluster that contains the *Bifidobacterium* genus and *E. coli*, indicated with a yellow box, probably does not represent a trophic network, as such, but is mainly driven by an association with young age. An example of this is the association of *Bifidobacterium* with species various strains of which are known to be linked with diarrhoea. A likely exception to this is the interaction between *Bifidobacterium* (a lactate producer) and *Megasphaera elsdenii*, a lactate-consuming butyrate producer^13–15^.

The most dominant species, *P. copri* (* in heatmap Fig. 5a), is grouped with a few other species just above the blue line into a small cluster, but is most likely an independent entity capable of reaching stable dominance without the help of other taxa by the age 12 months (Figs. 3 and 6a). *P. copri* together with the combined abundance of the *P. stercorea* cluster is strongly negatively correlated with the abundance of *Bacteroides* (Spearman’s rho = −0.358 and −0.363, respectively), as indicated by red boxes. The *Bacteroides* genus also falls within a small subcluster but does not share many characteristics in terms of correlations with other species outside this cluster.

A summary of the bacterial network is shown in Fig. 5b. Individual heatmaps from day 1 and day 85 samples show slightly different taxa in the main clusters but the overall pattern is very similar (Supplementary Fig. 6).

### Principal component analysis of the maturation process

Principal component analysis (PCA) combined with Spearman’s rho correlation analysis using clusters from Fig. 5a and important taxa (*P. copri*, *Bacteroides* and *Bifidobacterium*) was performed to analyse the maturation process in more detail. The *Faecalibacterium* cluster appears to benefit from a high abundance of *P. copri* (Fig. 6b), whereas the *P. stercorea* cluster does not have this positive correlation with *P. copri* (Fig. 6c). Principal component 1 (PC1) represents the maturation process as the accumulation of *P. copri* in samples (Fig. 6d). The average abundance of *P. copri* was so high that it alone could be used to describe PC1. PC2, however, describes a more interesting pattern that captures the antagonistic relationship between *P. copri* and the *P. stercorea* cluster (combined) with *Bacteroides* (Fig. 6e). PC3, by contrast, describes the transition away from *Bifidobacterium* and the build up of trophic networks, which include the *P. stercorea* cluster, the *F. prausnitzii* cluster and the shared auxiliary cluster (Fig. 6f). This pattern was the most strongly associated with age as can be visually ascertained.

## Discussion

In this study, we utilized a large cohort of children from an iron intervention trial in a rural region of The Gambia. This large study gave us an excellent opportunity to analyse in detail the microbiome of children during the first 40 months of life, a critical time frame in the development of the gut microbiome of humans in both industrialized and non-industrialized societies^1^. Our analyses showed that age was the main factor that determined the overall bacterial composition in these samples and highlighted the particular importance of the *Prevotella* genus. It has previously been established that *Prevotella* is the most important discriminatory taxon that differentiates rural Africans (and other non-industrialized populations) and people from industrialized countries^1,2,16^.

This study does not aim to disentangle the discussion about enterotypes yet it does provide insights regarding what has thus far been regarded as the *Prevotella* enterotype. This study shows that the *Prevotella* genus should not be seen as a monolithic group, except in its antagonistic relationship with *Bacteroides*, but rather as consisting of at least two important and seemingly independent components. In these children, who can be said to develop a *Prevotella* enterotype composition, *P. copri* rises to dominance rapidly in the first 12 months and remains the most abundant species during the remainder of early childhood (~35% of all reads), irrespective of the abundance of other species. Other *Prevotella* species are, however, part of a network that increases in abundance much more gradually over time in what has been coined in this study as the *P. stercorea* trophic network that, in these children, develops alongside another trophic network in which *F. prausnitzii* is the most dominant species.

We have summarized several published and well-known studies on the importance of *Prevotella* in Extended Data Table 3.

*Faecalibacterium prausnitzii* is known to be of importance in industrialized settings and is often associated with health; it is typically absent before the age of 4–6 months but then appears and its numbers increase steadily over time once weaning commences^12,17^. The *F. prausnitzii* trophic network development seen in this study mirrors the that seen in a study with Finnish and Estonian children^12^, although perhaps reaching a stable level approximately 1 year earlier in the Gambian children. The reasons for this are that the *F. prausnitzii* trophic network in Finnish/Estonian children can be said to be slightly more complex and as a whole more abundant in terms of percentage reads, whereas the *F. prausnitzii* trophic network in children from The Gambia appears to utilize the metabolic activity of *P. copri*, a species that independently reaches a very high stable prevalence by the age of 1 year. Build-up of a trophic network of fibre degraders, acetate and lactate producers combined with butyrate producers (*F. prausnitzii*) appears to be essential for healthy immune system development in children in industrialized counties^12^. Addition of the *P. stercorea* trophic network in our African cohort (alongside *P. copri*) probably helps explain why higher levels of short-chain fatty acid production are seen in African people with non-industrialized gut microbiomes^18,19^.

The trophic network of which *P. stercorea* is the most abundant member is typically not found or is found in only in low numbers in the gut microbiomes of individuals in industrialized countries^1,2,12,16^. As a result, this trophic network has often not been studied sufficiently (or at all) which makes it difficult to understand or explain, with any degree of certainty, what its roles are. The *P. stercorea* trophic network, as described here in the Gambian context, appears more diverse and self-contained than the *F. prausnitzii* network because it does not depend on *P. copri*. Although not all species within this network have been studied in detail, it is clear from what is known about many of these species is that the assemblage should have a very wide assortment of metabolic capabilities, covering everything needed to form a self-contained trophic network, leading (for example) to enhanced production of short-chain fatty acids compared with microbiome compositions lacking this cluster.

Similar to infant microbiotas in industrialized countries, the genus *Bifidobacterium* is the most important and dominant group early in infancy^20,21^ (reviewed in ref. ^22^). As infants are weaned, the dominance of bifidobacteria wanes and is rapidly replaced by bacteria associated with a more adult-like ‘African (study from Burkina Faso and Malawi)’ microbiome composition^1,2^, especially in the case of these Gambian children. *Bifidobacterium* levels at 3 years of age are much lower in children from The Gambia than in European children, possibly because the lactate- and acetate-producing bifidobacteria become part of the trophic networks found in Europe, whereas they are replaced by other species with a similar function (acetate and lactate production) in The Gambia. The higher abundance of *Bifidobacterium* in European children may also be a result of earlier and longer formula-feeding in those children^20,23^.

The high abundance of *P. copri* emphasizes its importance and the importance of its metabolic pathways in fermenting complex polysaccharides, which is important for people with a high-fibre diet. It has been shown in other studies that *Prevotella* plays an important role in glucose metabolism^24^, encodes all the enzymes necessary for the Embden–Meyerhof–Parnas pathway^25^, can degrade pyruvate to acetate and formate^26^ and is important in multiple pathways involving drug, carbohydrate and cofactor and vitamin metabolism^27^. In recent years, *P. copri* has been the subject of contradictory reports about whether it is beneficial or not; in particular, *P. copri* has been implicated as a risk for rheumatoid arthritis^28,29^. However, the largest *P. copri* metagenomic analysis using over 6,500 metagenomes by Segata et al. reported notable differences in carbohydrate metabolism driven by dietary modifications resulting in a reduced prevalence in westernized populations^30^. The same group also reported four distinctive globally distributed *P. copri* clades of country-specific subtypes, and in their meta-analysis found no strong evidence that any of the four clades were associated with disease. In this study, the high *Prevotella*-to-*Bacteroides* ratio (~10:1) also highlights an interesting intestinal dynamic that changes through industrialization and shifts in diet^31^. A high *Prevotella*-to-*Bacteroides* ratio pretreatment has been associated with more effective loss of body weight when utilizing high-fibre diets^32^.

An additional interesting detail is the abundance of *E. coli*. Not only was *E. coli* found to be most abundant in young children and to decrease over time, but it was also associated with the rainy season. From the literature it is known that diarrhoea is mainly associated with rotavirus infections in the dry season but with opportunistic pathogens during the rainy season^33^, including diarrhoeagenic *E. coli* strains.

In conclusion, our study has provided insights into the development of a *Prevotella*-rich gut microbiome. We were able to describe the steady development of a trophic network centred around *P. stercorea* and the rapid rise to dominance of *P. copri*. The high abundance of *Prevotella* in non-industrialized countries makes this genus the most discriminating taxon between a (healthy) high-fibre diet and an unhealthy low-fibre diet seen in industrialized countries. In industrialized countries, the increase in various diseases of affluence, such as diabetes, is typically associated with the (partial) loss of the last trophic network (indicated by lower *F. prausnitzii* numbers) and an increase in *Bacteroides* and/or Proteobacteria. The parallel loss of *P. copri* and the *P. stercorea* trophic network, whose development is described here, indicates the likely importance of the metabolic pathways and capabilities of *Prevotella* and species associated with them, both for maintaining a higher fermentative capacity (short-chain fatty acid production) and a functionally more diverse and healthier gut microbiome environment in regards to keeping diseases of affluence at bay.

## Methods

### Participants

#### Study design and children cohort

The stool samples used in this study were taken from the IHAT-GUT ‘The Iron Hydroxide Adipate Tartrate’ trial (NCT02941081), which is a three-arm, parallel, randomized, placebo-controlled, double-blind study with iron supplementation in young children with mild to moderate iron-deficient anaemia^9,34^. The study population in IHAT-GUT comprised children under the age of 3 years living in the north bank rural communities in the Upper River Region (URR) of The Gambia in West Africa. Informed consent for a child to participate in the study was provided by their parent before enrolment in the study.

The study area included 45 villages in the Wuli and Sandu districts, situated approximately 400 km east of the capital Banjul, on the north bank of the Gambia River. All villages had access to borehole tap water at central places and are typical of rural sub-Saharan Africa. A detailed description of the study design, child cohort, recruitment, screening, intervention and ethnic statement are present in Gates Open Research^9^. Stool samples were collected at baseline (day 1), day 15 and day 85. Stool samples collected in an OMNIgene GUT tube contain a DNA-stabilizing agent that ensures samples can be kept at ambient temperature for 60 days. Total stool DNA is extracted from these samples using the Mo Bio PowerLyzer PowerSoil DNA Isolation Kit (Qiagen) within 6 weeks of sample collection.

The trial was conducted in accordance with the ethical principles that have their origin in the Declaration of Helsinki, and that are consistent with the International Conference on Harmonisation (ICH) requirements for Good Clinical Practice (GCP), and the applicable regulatory requirements. The study sponsor was the London School of Hygiene and Tropical Medicine (LSHTM) and the study was conducted at the Medical Research Council (MRC) Unit The Gambia at LSHTM (MRCG).

The URR from which the cohort was recruited has an approximate population of 200,000 and only one major town, Basse; it is otherwise typical of rural sub-Saharan Africa. The URR has the highest mortality rate of children under 5 years in the country (92 deaths per 1,000 livebirths), the highest percentage of severely malnourished children (7–11%), and the highest prevalence of malaria and anaemia in children under 5 years (4.5% and 82.5%, respectively)^35^. The 45 villages in the study area had a population of approximately 2,800 children aged 6–35 months. All communities have access to borehole tap water at central places. Study specimen samples were collected at one of the study health clinics: Yorrobawol Health Centre, Darsilami Community Health Post, Konkuba Community Health Post, Taibatu Health Post and Chamoi Health Centre.

For this large clinical trial, the Kato-Katz method was used to collect information about helminth parasite egg count and faecal calprotectin. Very few children had helminths because there is a national programme of anti-helmitic metronidazole every 6 months in The Gambia. Children were not given anti-helminths at the start of the study. Regarding gut inflammation, the Gambia team did measure faecal calprotectin. This was a secondary outcome of the trial and will be reported in the main trial outcome paper.

#### Sample sizes

The sample size was chosen so that the IHAT-GUT study was adequately powered for the first primary objective: determining whether IHAT was non-inferior to FeSO_4_ on the day 85 response outcome. It was assumed based on previous evidence that the proportion of children who were responders with FeSO_4_ at day 85 would be 0.3. The non-inferiority margin was an odds ratio of 0.583 (equivalent to a 0.1 absolute difference in response probability). Because any significant result would be tested in a subsequent pivotal (phase III) study, a 10% one-sided type I error rate was used. A sample size of 200 per arm provides 89% power to demonstrate non-inferiority when the two arms have the same response probability.

As described further in the protocol, the sample size of 200 per arm also provides: (1) 90% power (10% one-sided type I error rate) for testing the superiority of IHAT over FeSO_4_ for the prevalence of diarrhoea when prevalence is 0.15 in the IHAT arm and 0.25 in the FeSO_4_ arm; (2) 93% power (10% one-sided type I error rate) for testing the non-inferiority of IHAT versus placebo for diarrhoea prevalence when it is 0.15 in the IHAT and placebo arms with a 0.1 absolute non-inferiority margin; (3) 90% power (10% one-sided type I error rate) to find a reduction in the incidence density of diarrhoea in IHAT versus FeSO_4_ assuming 1.28 episodes per child over the 85 days in the FeSO_4_ arm and rate ratio of 0.8. For the secondary outcomes, the trial (*n* = 200 per arm) would have over 85% power to detect significant differences between all the arms in terms of enterobacteria, non-transferrin-bound iron and calprotectin. To account for an anticipated 15% non-completion rate, based on previous studies in The Gambia, the target sample size was set to 705. Because this was a phase II trial aiming to determine whether a phase III trial was warranted, no adjustment for multiple testing was made.

#### Randomization

Randomization was performed using a stratified block design to achieve group balance in terms of age (6–11 months, 12–23 months and 24–37 months) and baseline haemoglobin concentration (above and below median, calculated for each cohort separately) at pre-enrolment (day 0). Within each of the six resulting strata, children were randomly assigned to one of the three study treatment arms (1:1:1 ratio) using a computer program written by the trial statistician and a block randomization approach with fixed block size of six was used.

#### Nutritional and diet information

The Gambia is a low-income country in West Africa, where food availability and nutritional status in rural areas are poor, are strongly influenced by season, and a chronically marginal diet is exacerbated by a ‘hungry season’ (July to September), when food stocks from the previous harvest season are depleted. Infants in rural Gambia are breastfed to 2 years of age, with fewer than half of infants being exclusively breastfed to 6 months of age as per WHO recommendation^36^. The first foods introduced from 3 months of age are thin gruels made from only cereal, water (occasionally cow’s milk is added), salt and sugar, and are of a low energy and fat content. A thicker porridge made from rice and pounded groundnuts is sometimes administered. Cow’s milk alone is given infrequently to infants <1 year of age; only 57% of infants receive it more than once a week, although it is provided often to children in the second year of life. From 6 months, infants start to share the family food bowl, the most common meals consisting of boiled rice and a sauce made from groundnuts or leaves. Dried fish may be added to sauces in very small quantities, but fresh fish is not given to infants before 9 months^37^.

#### Sampling framework and sample characteristics

We performed 16S rRNA amplification and sequencing on a total of 1,546 samples (1,466 stool samples, 46 negative controls and 34 positive controls). In total, we generated 146,603,504 bacterial V1V2 16S rRNA gene reads. Fifty-six stool samples had fewer than 1,000 high-quality reads and were removed from further analysis; this was mainly due to watery samples containing very low biomass which did not provide sufficient DNA for amplification. Of the remaining 1,410 samples, 1,372 were subsampled to 20,000 high-quality reads per sample and for 38 samples (with 1,000–20,000 sequence reads) all reads were kept. Of the 1,410 samples left after quality filtering, five more samples were removed by the Oligotyping filtering step (reads <1,000), leaving 1,405 samples. These samples were collected at three different time points during an iron intervention trial in The Gambia^9^ and hence were available for detailed developmental and bacterial trophic network analysis.

#### Participant exclusion

Children with severe malnutrition were excluded from the trial (*n* = 88, 6% of children who were screened; *z*-scores for length/height-for-age, weight-or-age or weight-for-length/height of −3 s.d. or less). Mean *z*-scores for the included children were around −1. Data that failed the high-quality control procedure in the bioinformatics pipeline were also excluded, that is any samples with a low amount of DNA from which no reads >1,000 were obtained. This excluded 61 of 1,466 samples and an additional small number of 16 samples from 15 children who received antibiotics were also removed leaving 1,389 samples from 633 children for detailed analysis. Antibiotic treatment affected 15 of the day 85 samples and one day 15 sample. Therefore, not all children in the IHAT-GUT trial were included in this study.

### Nucleic acid extraction

Extraction of total genomic DNA was conducted on stool samples collected on visit days 1, 15 and 85, using the MO BIO Laboratories (now Qiagen) DNeasy PowerLyzer PowerSoil Kit (catalogue number: 12855-100). Each extraction was done with 24 samples (23 study samples and one reagent blank). About 250 μl of the OMNIGENE (OMNIgene•GUT|OM-200; DNA Genotek) sample mix (from a total of 2 ml of sample plus stabilizing liquid mix) was aliquoted into a labelled PowerLyzer glass bead tube (0.1 mm; catalogue number: 13118-100-GBT) and then mixed gently with 750 μl of PowerSoil Bead Solution (catalogue number: 12855-100-BS). About 60 μl of solution C1 (catalogue number: 12888-100-1) was then added and vortexed briefly. The samples were then homogenized for 45 s at 3000 r.p.m. using a Mo Bio PowerLyzer24 bead beater (catalogue number: 13155). About 400–500 μl of supernatant was transferred to a clean 2-ml collection tube (catalogue number: 12888-100-T) following centrifugation of the bead tubes at 10,000*g* for 30 s at room temperature. The supernatant was then subjected to several purification steps. To precipitate any non-DNA material, 250 μl of solution C2 (catalogue number: 12888-100-2) was added, the supernatant vortexed and then incubated at 4 °C for 5 min. The samples were centrifuged at room temperature for 1 min at 10,000*g* and up to 600 μl of supernatant was transferred into another clean 2-ml collection tube. About 200 μl of solution C3 (catalogue number: 12888-100-3) was then added and the sample vortexed briefly and incubated at 4 °C for 5 min. About 750 μl of supernatant was then collected into a clean 2-ml collection tube following centrifugation of the sample and solution C3 mix at room temperature for 1 min at 10,000*g*. This was followed by the addition of 1,200 μl of solution C4 (catalogue number: 12888-100-4) to the supernatant, which was then vortexed for 5 s. Using PowerSoil spin filter units in 2-ml tubes (catalogue number: 12888-100-SF), 675 μl of supernatant was loaded and filtered at 10,000*g* for 1 min at room temperature. The flow through was discarded and the step was repeated two more times. About 500 μl of ethanol-based solution C5 (catalogue number: 12888-100-5) was aliquoted into the spin filter and centrifuged at room temperature for 30 s at 10,000*g* and the flow through discarded. To remove any residual solution C5, the spin filter was again centrifuged at room temperature for 1 min at 10,000*g*. The spin filter was then carefully transferred into a clean 2-ml collection tube while avoiding splashing solution C5 onto the spin filter. Finally, about 110 μl of solution C6 is added to the centre of the white filter membrane before centrifugation at room temperature for 30 s at 10,000*g*. The spin filter is then discarded and the DNA solution aliquoted into two clean 2-ml collection tubes and stored at −80 °C for downstream processing. The DNA concentration was occasionally measured on random samples to assess sample concentration and purity using a NanoDrop ND-1000 UV-Vis spectrophotometer.

### Bacterial 16S rRNA gene library preparation and Illumina MiSeq sequencing

The bacterial 16S rRNA V1V2 variable region of extracted DNA was amplified with Illumina adapter and indexed PCR primers using a dual-index sequencing strategy to target the bacterial 16S rRNA gene^38^. Each PCR reaction was done in triplicate in a total reaction volume of 25 μl together with 200 μM deoxynucleotide triphosphates (dNTPs), 0.5 μM V1 forward primers (7f 5′-AATGATACGGCGACCACCGAGATCTACAC- XXXXXXXX-acactctttccctacacgacgctcttccgatct- NNNN-AGMGTTYGATYMTGGCTCAG-3′), 0.5 μM V2 reverse primer (r356 5′-CAAGCAGAAGACGGCATACGAGAT- XXXXXXXX-gtgactggagttcagacgtgtgctcttccgatct- NNNN-GCTGCCTCCCGTAGGAGT-3′), and 0.25 μl of Q5 Taq enzyme. The Illumina adapter primer sequence is built of Illumina adapter, 8 bp index sequences (8 Xs), binding side for Illumina sequencing primer (lower case letter), four maximally degenerated bases (NNNN) to maximize diversity during the first four bases of the run and a PCR target sequence. Cycling conditions were as follows: denaturation at 98 °C for 2 min, followed by 30 cycles of amplification (denaturation 98 °C for 30 s, annealing 50 °C for 30 s, extension 72 °C for 90 s) and a final extension at 72 °C for 5 min. All primers were purchased from Metabion International AL AG. Triplicate PCR reactions were pooled and purified with 75 μl of Agencourt AMPure XP (catalogue number: A63881) according to Illumina’s 16S metagenomic sequencing library preparation protocol, pages 8–9 (part number: 15044223 Rev. B; https://support.illumina.com). DNA concentrations were quantified using the Invitrogen Qubit 3.0 fluorometer (catalogue number: Q33216) and Qubit double-stranded DNA HS assay kit (catalogue number: Q32854). Samples were pooled in equimolar concentrations and gel purified using the Wizard SV Gel and PCR Clean-Up System (Promega). The library size was confirmed on a QIAxcel Advanced (Qiagen) and then MiSeq sequenced using the 600 cycle MiSeq reagent kit V3, which enables 300-bp end sequencing. The library was sequenced at the Wellcome Sanger Institute (Cambridge, UK). A total of 1,546 samples including negative (*n* = 45) and (*n* = 34) positive controls were sequenced in 18 MiSeq libraries.

### Bioinformatics and statistics

#### Bacterial 16S rRNA maker gene analysis

The forward and reverse fastq files of each sample were processed according to the MOTHUR MiSeq SOP with some modifications (MOTHUR wiki at http://www.mothur.org/wiki/MiSeq_SOP). The ‘make.contigs’ command was used with no extra parameters^39^. The assembled contigs were taken out from the MOTHUR pipeline and the four poly(NNNN)s present in the adapter/primer sequences were removed using the ‘-trim_left 4’ and ‘-trim_right 4’ parameters in the PRINSEQ program^40^. The PRINSEQ-trimmed sequences were used for the first ‘screen.seqs’ command to remove ambiguous sequences (maxambig = 0) and sequences containing homopolymers longer than 8 bp (maxhomop = 8). The quality-screened sequences were aligned using the Silva bacterial database ‘silva.nr_v123.align’ with the flip parameter set to true. Any sequences outside the expected alignment coordinates were further removed using the ‘screen.seqs’ command. The alignment coordinates were set with ‘optimize = start-end, criteria = 90’. In addition, any sequences longer than 400 bp were remove with ‘maxlength = 400’. The correct aligned sequences were filtered using the ‘filter.seqs’ command with ‘vertical = T’ and ‘trump = .’. The subsequent filtered sequences were de-noised by allowing three mismatches in the ‘pre.clustering’ step and chimeras were removed using Uchime with the dereplicate option set to ‘true’. The chimera-free sequences were classified using the Silva reference database ‘silva.nr_v123.align’ and the Silva taxonomy database ‘silva.nr_v123.tax’ and a cut-off value of 80%. Chloroplast, Mitochondria, unknown, Archaea, and Eukaryota sequences were removed. The high-quality, chimera-free and correct classified sequences were normalized using the ‘sub.sample’ command. Each sample was normalized to 20,000 reads. This removed 94 samples with reads below 20,000 per sample from a total of 1,546 samples. Thirty-eight of the 94 samples with reads between 1,000 and 20,000 per sample were added back to the dataset. One mislabelled sample (negative control outlier) was also removed from the dataset, leaving 1,489 samples available for Oligotyping.

#### Oligotyping and taxa identification

Oligotyping was used for clustering the high-quality filtered fasta sequences from the MOTHUR pipeline. Oligotyping is a computational method to investigate the diversity of closely related by distinct bacterial organisms in final operational taxonomic units identified in environmental data sets through 16S rRNA gene data by the canonical approaches. For oligotyping we used the ‘Minimum Entropy Decomposition’ (MED) option for sensitive partitioning of high-throughput marker gene sequences from the oligotyping pipeline^41^. The normalized high-quality fasta and name file from MOTHUR were renamed by appending the group name to the sequence name, using the ‘rename.seqs’ command. A redundant renamed-fasta file was then generated using the ‘deunique.seqs’ command, which creates a redundant fasta file from a fasta and name file. The redundant fasta file was subsequently used for oligotyping using the unsupervised MED. The command line was ‘decompose <fasta.file> –g –t - -M 100 -V 2’ The –t character which was set to a dash ‘-’ character. The dash character was used in the MOTHUR ‘rename.seqs’ command to separate the sample name from the unique info in the defline of the sequence name. The -M integer defines the minimum substantive abundance of an oligotype and the -V integer defines the maximum variation allowed in each node. This MED settings generated 10,152 oligotypes. The node representative sequence of each oligotype was used for species profiling using the ARB program (v.5.5-org-9167)^42^. For ARB analysis we used a customized version of the SILVA SSU Ref database (NR99, release 123) that was generated by removing environmental and uncultured taxa.

ARB-generated short species abbreviations were then correlated with the full taxonomic path from species to phyla. The 10,152 redundant ARB species were then consolidated to non-redundant 524 species which were present in the 1,410 samples with a minimum substance abundance of an oligotype per node of 100 (-M setting from above). Consolidation was performed using the ‘Consolidate’ option in Excel for Mac v.16.16.14; Microsoft). In cases in which a species could not be classified, we reported the genus name and in few cases the family name. For some obvious beneficial or pathogenic genera, we combined all species within the same genus, for example, for the purposes of ecological functionality, pattern recognition and visualisation of associations we combined all *Bifidobacterium* species together and the same was done for all *Bacteroides* species.

#### Alpha-diversity analysis

Alpha-diversity indexes for Fisher’s alpha parameter, Simpson’s index, Chao1 richness index, and Richness index (observed richness) were calculated using the online web portal Calypso v.8.84 (ref. ^1^) using TSS transformation followed by CSS normalization, a widely used method for normalizing microbial community compositional data. CSS correct for biases introduced by TSS^2^. The CSS selection automatically performs a log_2_ transformation to account for the non-normal distribution of taxonomic counts data.

#### Beta-diversity analysis

Multivariate beta-diversity analysis was performed using PERMANOVA, and one- and two-way ANOSIM in the statistical software package PAST4 (v.3.20)^5^. The similarity index was set to Bray–Curtis and for the permutation N we used the default of 9,999. For the pairwise PERMANOVA test, we reported the Bonferroni-adjusted *P* value and the *F* statistic value, and from the ANOSIM test we reported the Bonferroni-adjusted *P* value and the *R* value.

#### Principal coordinate analysis

For the visualization of microbial compositional differences between environmental variables (age group, time point, treatment, season and geographic location) we plotted the microbial variances using a multivariate method called PCoA for taxon level ‘species’. For the visualization of microbial differences between the three young, middle and old age groups, we analysed the datasets at the species, genus and family levels using PCoA. The PCoA was done using the online web portal Calypso v.8.84 (ref. ^43^). The PCoA was done on the Bray–Curtis matrix (default settings in Calypso). For PCoA, absolute abundances were transformed to relative abundance using TSS. The TSS dataset was then normalized using CSS, a widely used method for normalizing microbial community compositional data. CSS correct for biases introduced by TSS^44^. The CSS selection automatically performs a log_2_ transformation to account for the non-normal distribution of taxonomic counts data.

#### Determination what species changes over time using MaAsLin2

To determine which taxa are associated with age, season and treatment groups, we use “a multivariable statistical framework for finding associations between clinical metadata and potentially high-dimensional microbial multi-omics data” through the R package MaAsLin2 v.1.5.1 (from The Huttenhower Lab: https://huttenhower.sph.harvard.edu/tools)^45^

Before analysis in MaAsLin2, absolute taxa abundancies were normalized to proportional abundance (TSS) using the R function ‘make_relative’ from the ‘funrar’ R package. TheTSS normalized data were then used for a mixed-effect modelling using ‘Maaslin2’ function from the ‘MaAsLin2’ R package. Age, treatment group (iron supplement or placebo) and season (wet or try) were used as ‘fixed effects’ and subject (for repeated sampling) was used as a random effect. Subject is unique to each child from which we had either one, two, or three samples. The minimum abundance was set to 0.01 and the minimum prevalence set to 0. Please note that the ‘subject’ variable (individual identifier for longitudinal models) was included only as a ‘random_effects’ parameter when all time point samples were analysed together. We also analysed samples from day 1, day 15 and day 85 collections separately. The R code is: fit_data = Maaslin2(input_data = countdata_TSS, input_metadata = metadata, output = ‘output’, fixed_effects = c(‘group’,‘age”,‘season’), random_effects = c(‘subject’), min_abundance = 0.01, min_prevalence = 0, normalization = ‘none’, transform = ‘none’)

From MaAsLin2 analysis, we report all taxa that were statistically significantly different with a *q* value.

#### Data plotting and statistical analysis

Data were plotted in GraphPad Prism 9 for macOS and by using macOS Keynote 11.1.

For statistical analysis, data were tested to check whether they follow a Gaussian distribution using the available tests including Anderson-Darling and the D’Agostino & Pearson test in GraphPad Prism 9. The non-parametric Kruskal–Wallis test was used for three or more groups when not normally distributed. For two-group comparison, the two-tailed unpaired non-parametric Mann–Whitney test was used for non-normally distributed data and normally distributed data were tested with a two-tailed unpaired parametric *t* test.

Statistical analysis was performed in R, PAST4 or in Calypso Web portal for the ALDEx2 test. The *P* value was corrected using the FDR method.

Mixed-effect linear regression was done in R using the lmer function from R package lme4 v.1.1-27.1. The model estimates the mean difference between categories for each taxon; for example, the difference between day 15 and day 1 as well as between day 85 and day 1 in the three time point analysis. Units are TSS + CSS log_2_(transformed and normalized abundances).

#### Bacterial tropic network analysis

The heatmap used to visualize bacterial trophic networks was generated using the online web portal Calypso v.8.84 (ref. ^9^) using TSS transformed abundances. For easier visualization we used the top 50 species with a minimum abundance of 0.2% in all samples. Blocks representing apparent trophic networks or other types of associations were colour coded on the heatmap. Gap statistic using the R function ‘clusGap’ from the R package ‘cluster’ v.2.1.2 was used to calculate a goodness of clustering measure, the ‘gap’ statistic. The ‘k.max’ parameter was set to 10, the bootstrap ‘B’ parameter was set to 100, and the analysis was done with two different ‘FUNcluster’ method including cluster:fanny and kmeans. The bacterial tropic network summary was generated from Spearman’s rho correlation coefficient data.

### Reporting Summary

Further information on research design is available in the Nature Research Reporting Summary linked to this article.

### Supplementary information

Supplementary Information Flow diagram showing the samples taken for the secondary study outcome (microbiome analysis). Alpha-diversity analysis. Beta-diversity analysis. Supplementary Figs. 1–7. Supplementary Fig. 1 PCoA for treatment and placebo. Supplementary Fig. 2 PCoA for gender and location. Supplementary Fig. 3 PCoA for wet and try season. Supplementary Fig. 4 Mixed effect linear regression enrolment 3 age groups time effect. Supplementary Fig. 5 Mixed effect linear regression enrolment 11 age groups time effect. Supplementary Fig. 6 Heatmap bacterial trophic network day 1 top 50 taxa. Supplementary Fig. 7 Heatmap bacterial trophic network day 85 top 50 taxa.

Reporting Summary

Peer Review Information

Supplementary Tables 1–6 Supplementary Table 1 Statistical analysis to identify discriminant taxa between the treatment and placebo groups. Supplementary Table 2 Alpha diversity multiple group comparison with Kruskal–Wallis Dunn’s test. Supplementary Table 3 Beta-diversity analysis (PERMANOVA and ANOSIM) for 11 age group comparison. Supplementary Table 4 Beta-diversity analysis (PERMANOVA and ANOSIM) for the wet and try season groups. Supplementary Table 5 Statistically significant associated bacterial taxa with age, season (wet or try), and treatment group (iron supplement or placebo). Supplementary Table 6 Metadata and count data for all 1,389 samples.

## Figures and Tables

**Extended Data Table 1:**
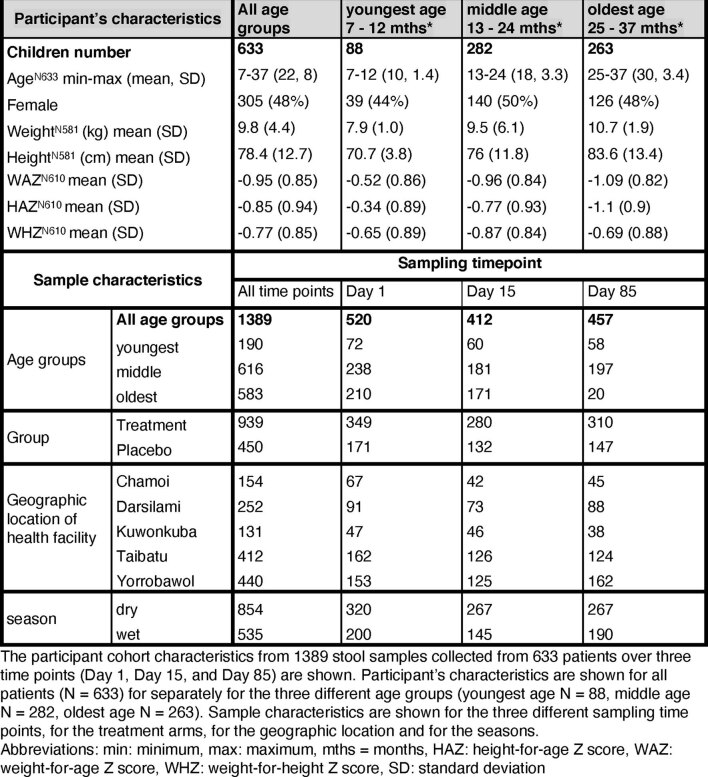
Child cohort characteristics available for microbiome analysis

The participant cohort characteristics from 1389 stool samples collected from 633 children over three time points (Day 1, Day 15, and Day 85) are shown. Participant’s characteristics are shown for all patients (N = 633) and separated for the three different age groups (youngest age N = 88, middle age N = 282, oldest age N = 263). Sample characteristics are shown for the three different sampling time points, for the treatment arms, for the geographic location and for the seasons. Abbreviations: min: minimum, max: maximum, mths = months, HAZ: height-for-age Z score, WAZ: weight-for-age Z score, WHZ: weight-for-height Z score, SD: standard deviation.

**Extended Data Table 2:**
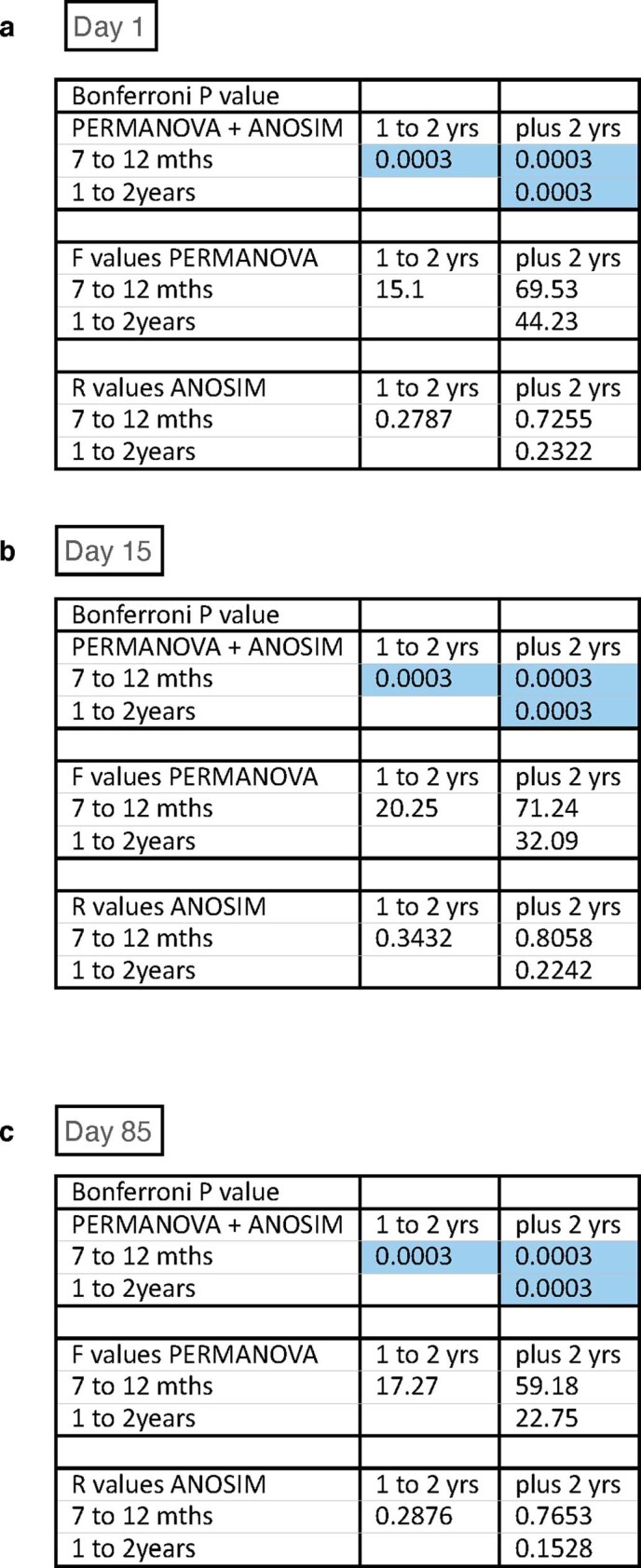
Beta diversity analysis (PERMANOVA and ANOSIM) for the three age group comparison

Beta diversity analysis (PERMANOVA and ANOSIM) for the three age group comparison.

PERMANOVA test and ANOSIM test were performed in PAST (Paleontological Statistics software package for education and data analysis), version 4.04. From the PERMANOVA test the Bonferroni corrected P value and F value is reported. From the ANOSIM test, the Bonferroni corrected P value and the R value is reported. Both tests were done separately for the Day 1 (N = 520), Day 15 (N = 412) and Day 85 (N = 457) samples. All Bonferroni corrected P value < 0.05 are highlighted in blue.

**Extended Data Table 3:**
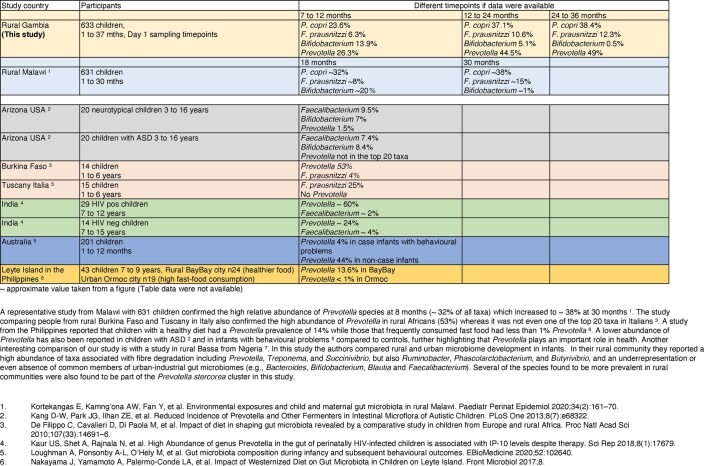
Selected studies with *Prevotella* Importance mentioned and discussed in the manuscript

A representative study from Malawi with 631 children confirmed the high relative abundance of *Prevotella* species at 8 months (~ 32% of all taxa) which increased to ~ 38% at 30 months^3^. The study comparing people from rural Burkina Faso and Tuscany in Italy also confirmed the high abundance of *Prevotella* in rural Africans (53%) whereas it was not even one of the top 20 taxa in Italians^2^. A study from the Philippines reported that children with a healthy diet had a *Prevotella* prevalence of 14% while those that frequently consumed fast food had less than 1% *Prevotella*^46^. A lower abundance of *Prevotella* has also been reported in children with ASD^47^ and in infants with behavioural problems^48^ compared to controls, further highlighting that *Prevotella* plays an important role in health^49^. Another interesting comparison of our study is with a study in rural Bassa from Nigeria^7^. In this study the authors compared rural and urban microbiome development in infants. In their rural community they reported a high abundance of taxa associated with fibre degradation including *Prevotella*, *Treponema*, and *Succinivibrio*, but also *Ruminobacter*, *Phascolarctobacterium*, and *Butyrivibrio*, and an underrepresentation or even absence of common members of urban-industrial gut microbiomes (for example, *Bacteroides*, *Bifidobacterium*, *Blautia* and *Faecalibacterium*). Several of the species found to be more prevalent in rural communities were also found to be part of the *Prevotella stercorea* cluster in this study.

## Data Availability

The metadata and bacterial 16S count data used for the analysis are available in Supplementary Table 6. The raw bacterial 16S sequence data are available from the European Nucleotide Archive (ENA) with the following accession number “ERP110905”.

## Code availability

For this analysis no custom code was used.
