# Supplementary material for: Gut microbiomes from Gambian infants reveal the development of a non-industrialized Prevotella-based trophic network
Source: Nat Microbiol. 2021 Dec 31;7(1):132–44. doi: 10.1038/s41564-021-01023-6 (PMC8727306; doi:10.1038/s41564-021-01023-6)
Supplement: Supplementary file 1 — Flow diagram showing the samples taken for the secondary study outcome (microbiome analysis). Alpha-diversity analysis. Beta-diversity analysis. Supplementary Figs. 1–7. Supplementary Fig. 1 PCoA for treatment and placebo. Supplementary Fig. 2 PCoA for gender and location. Supplementary Fig. 3 PCoA for wet and try season. Supplementary Fig. 4 Mixed effect linear regression enrolment 3 age groups time effect. Supplementary Fig. 5 Mixed effect linear regression enrolment 11 age groups time effect. Supplementary Fig. 6 Heatmap bacterial trophic network day 1 top 50 taxa. Supplementary Fig. 7 Heatmap bacterial trophic network day 85 top 50 taxa. [file 41564_2021_1023_MOESM1_ESM.pdf]

nature portfolio

https://doi.org/10.1038/s41564-021-01023-6

Supplementary information

Gut microbiomes from Gambian infants reveal the development of a non-industrialized *Prevotella*-based trophic network

In the format provided by the authors and unedited

Supplementary information for

Gut microbiomes from Gambian infants reveals the development of a non-industrialised Prevotella-based trophic network

Contains:

Flow diagram showing the samples taken for the secondary study outcome (microbiome analysis)

Alpha diversity analysis

Beta diversity analysis

Supplementary figures 1 – 7

Supplementary Figure 1 PCoA Treatment and Placebo

Supplementary Figure 2 PCoA for Gender and Location

Supplementary Figure 3 PCoA for wet and try season

Supplementary Figure 4 Mixed Effect Linear Regression Enrolment 3 age groups Time Effect

Supplementary Figure 5 Mixed Effect Linear Regression Enrolment 11 age groups Time Effect

Supplementary Figure 6 Heatmap Bacterial Trophic network Day 1 top 50 taxa

Supplementary Figure 7 Heatmap Bacterial Trophic network Day 85 top 50 taxa

Flow Diagram 1. Showing samples taken for microbiome analysis as secondary outcome from the IHAT iron intervention study in The Gambia

Stool samples were taken at assessment day 1, day 15, and day 85, from the IHAT, FeSO4, and Placebo arm.

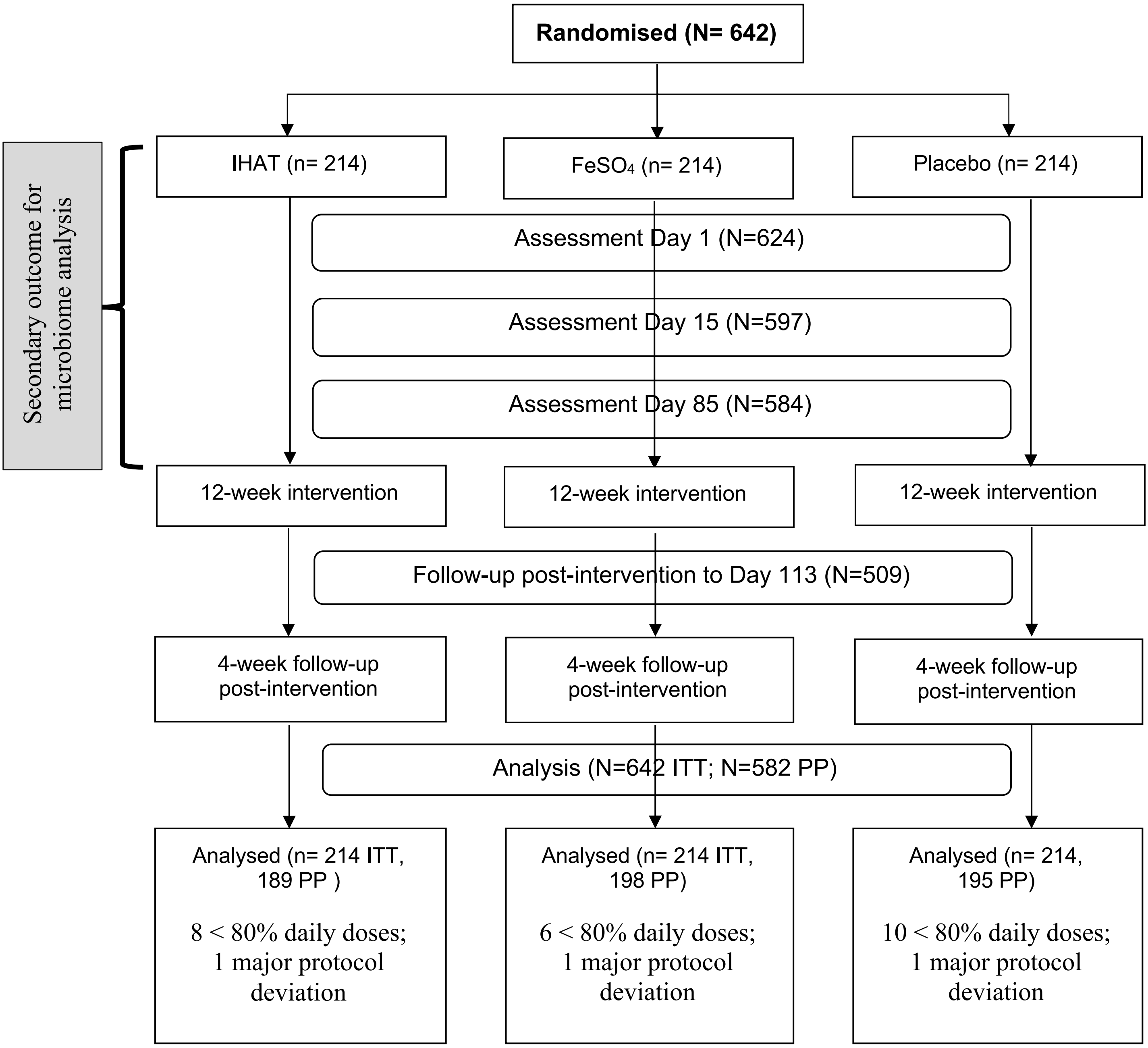

Alpha diversity analysis

The Fisher's alpha parameter was first descripted as a logarithmic series model in 1943 [3] and attempt to describe mathematically the relationship between the number of species and the number of individuals in those species. The Fisher's alpha parameter description was adapted from the University of Camerion, Italy, School of Biosciences and Veterinary at URL http://groundvegetationdb-web.com/ground_veg/home/diversity_index (accessed 24th and 25th Oct 2019). The Fisher's alpha parameter was originally used as an appropriate fit to empirical data, its wide application, especially in entomological research, has led to a thorough examination of its properties [4]. The small number of abundant species and the large proportion of 'rare' species (the class containing one individual is always the largest) predicted by the log series model suggest that, like the geometric series, it will be most applicable in situations where one or a few factors dominate the ecology of a community. Thus, we consider the Fisher's alpha parameter as a perfect additional alpha diversity index to the commonly report Simpson and Shannon index for any bacterial 16S datasets which are always dominated by few dominant species and many dominant species. The alpha diversity graphs and statistical analysis for two group comparison and for more than two group comparisons was done in Graph Pad Prism 9 for macOS using the non-parametric Mann-Whitney U test and the non-parametric Kruskall-Wallis test, respectively or non-normally distributed data with the parametric t test for normally distributed data for two groups comparison.

Significant changes in alpha diversity over time from 7 to 40 months of age

Additional justification to combine treatment and placebo groups came from the analysis of commonly reported alpha diversity indexes. The Alpha diversity indexes for Fisher's Alpha Simpson's, Chao 1, and Richness were non-significantly different between the treatment and

placebo group in the individual three different sampling timepoints i.e., Day 1, Day 15 and DAY 85 (**Extended Data Fig. 1**).

To follow Alpha diversity changes over time in the whole data set, we split the data into 11 age groups, separated by three-month intervals based on age group at sampling. The Fisher's alpha parameter indicated a statistically significant increase in alpha diversity from the youngest age group (7-9 months) to the oldest (37-40 months) age group (Kruskal-Wallis P <0.0001) and a gradual increase in between (**Extended Data Fig. 2a**). The Simpson's index was also statistically significantly different between the 11 age groups, but a gradual upwards trend as seen by the Fisher's alpha was not observed (Kruskal-Wallis P = 0.0002) (**Extended Data Fig. 2b**), largely because *Prevotella copri* reached a dominant and stable level (35-40% of all reads) already after 12 months thus Simpson's index performs somewhat poorly because of the high prevalence of *Prevotella copri*. Both the richness estimator index Chao1 (**Extended Data Fig. 2c** and the observed richness (**Extended Data Fig. 2d**) increased over time and this increase was highly significant for both tests (Kruskal-Wallis P <0.0001). 39 out of 55 pairwise comparisons of 3-months age groups were significant, with a FDR corrected P-value <0.05, for the Fisher's alpha, Chao 1, and Richness test. For the Simpson's test only three pair wise comparisons were significant (**Sup Table 2**).

The Alpha diversity shown in **Extended Data Fig 2** and **Sup Table 2** was done on the combined timepoints (day 1, day 15, and day 85) dataset. We also analysed the Alpha diversity indexes separated by the three different timepoints (**Extended Data Fig 3)**. The Fisher's alpha diversity increased continuously across the 3 months age groups in a similar smooth fashion as for the combined timepoint dataset with the overall Kruskall-Wallis test being highly significant (P <0.0001). The Simpson index again performed poorly in comparison but the increase of the Chao 1 and observed Richness indexes were also again

significant for all three individual timepoints (P <0.001) yet the pattern of gradual increase was more erratic due to having a smaller number of samples.

Beta diversity analysis

<u>Beta diversity between age groups</u>

PCoA together with PERMANOVA and ANOSIM was applied for Beta diversity analyses to elucidate compositional microbial differences/similarities between additional participant-related and environmental variables apart for the analysis between treatment and placebo groups. The PCoA of the gut microbiome stratified into three age group (7 to 12 mths, 1 to 2 years, and plus 2 years, age taken at time of sampling) showed distinctive clusters with the youngest and oldest group separated most from each other. This was true for the combined time point analysis (**Extended Data Fig. 4a)** and for the individual timepoints (**Extended Data Fig 4b for day 1 samples, 4c for day 15 samples, and 4.d for day 85 samples**). The Bonferroni corrected P-value from the PERMANOVA test and ANOSIM test between the three different age groups was 0.0003 and the calculated F statistic from the PERMANOVA and the R2 from the ANOSIMI test was always larger between the young and oldest age group (**Extended Data Table 2**). We also performed Beta diversity test for the 11 age group comparisons, age taken at time of sampling. The PCoA analysis for the combined timepoint analysis (**Extended Data Fig. 5a**) and for the individual timepoints (**Extended Data Fig. 5b for day 1 samples, 5c for day 15 samples, and 5d for day 85 samples**) shows a clear chronological step wise clustering for the 11 different age groups. Pair-wise PERMANOVA and ANOSIM show that the microbiome composition was significantly different between most of the different three months age groups (highlighted in blue in the PERMANOVA and ANOSIM in **Supplementary Table 3**).

Beta diversity between gender and geographic locations

The microbial composition between gender and the five different geographic locations clustered very much together in the PCoA. This was confirmed in the combine timepoint datasets and for the individual timepoints datasets **(Supplementary Figure 2**).

Beta diversity between wet and try season

PCoA analysis between wet season and dry season samples revealed a shift in the 60% concentration ellipse (**Supplementary Figure 3**). One-way PERMANOVA and ANOSIM reported a significant difference between wet and try season for all three different timepoints (day 1, day 15 and day 85 samples (**Supplementary Table 4**).

We tested whether age had an effect on the observed microbial differences between wet and dry season by a two-way ANOSIM test and included the 11-age group variable as a second factor in the analysis. Age did not seem to be a confounding factor in day 1 and day 15 samples. However, age appeared to have a confounding effect in day 85 samples because the addition of the 11-age group factor changed the significant season one-way ANOSIM P-value of 0.0001 to 0.3 in the two-way ANOSIM test (**Supplementary Table 4, Day 85 table**).

References:

1. Zakrzewski M, Proietti C, Ellis JJ, et al. Calypso: a user-friendly web-server for mining and visualizing microbiome–environment interactions. Bioinformatics 2017;33(5):782–3.
2. Paulson JN, Talukder H, Pop M, Bravo HC. Robust methods for differential abundance analysis in marker gene surveys. Nat Methods 2013;10(12):1200–2.
3. Fisher RA, Corbet AS, Williams CB. The Relation Between the Number of Species and the Number of Individuals in a Random Sample of an Animal Population. J Anim Ecol

1943;12(1):42.

4. TAYLOR LR. Diversity of insect faunas (Symposia of the Royal Entomological Society of London). illustrate. Balckwell, Oxford, Royal Entomological Society, 1978; 1978.

5. Hammer Ø, Harper DAT, Ryan PD. PAST: Paleontological statistics sofware package for education and data analysis. Palaeontol Electron 2001;4(1):1–9.

Supplementary Figure 1. PCoA volcano plot analysis for the treatment and placebo groups

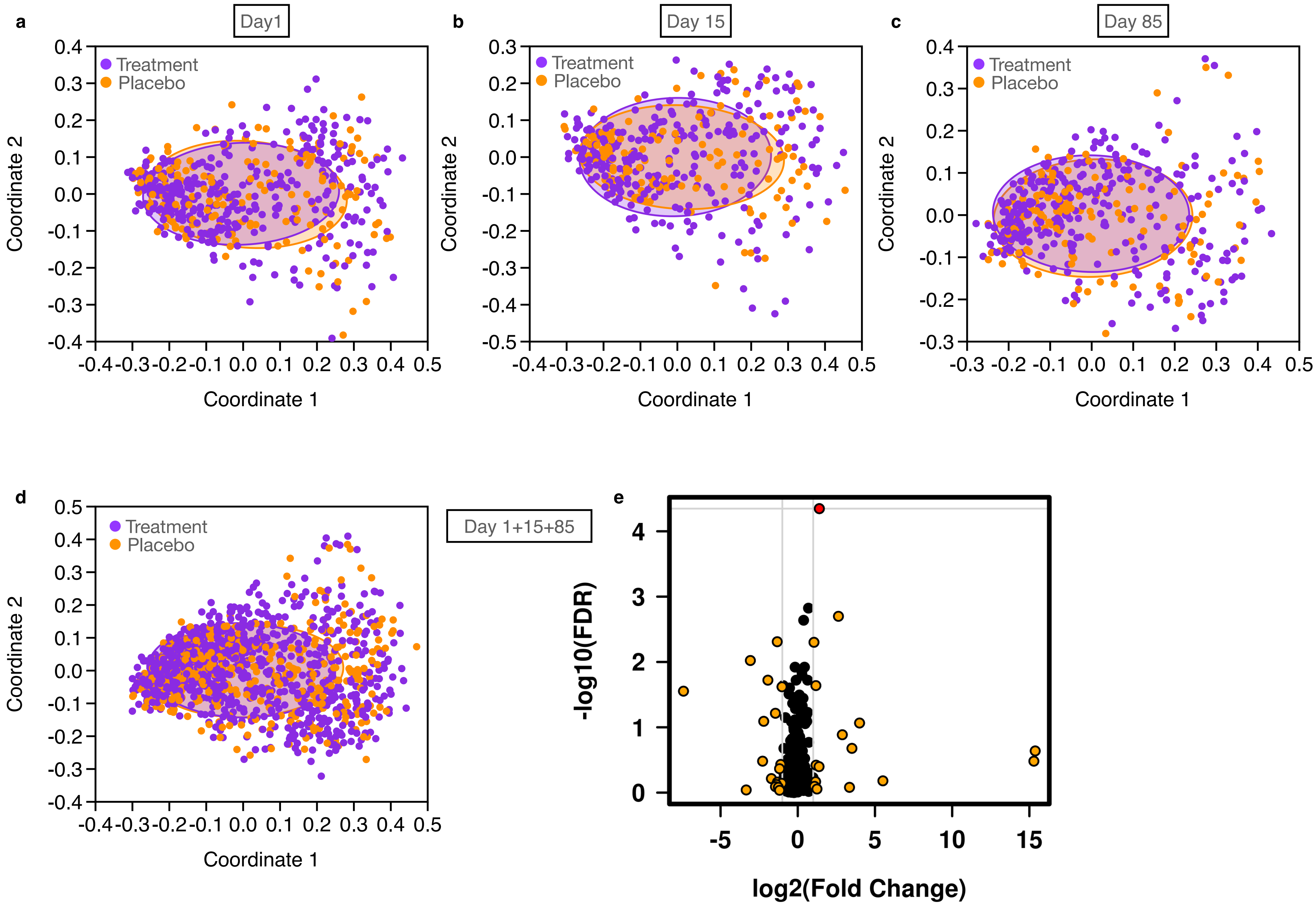

Supplementary Fig. 1 I PCoA and Volcano plot analysis for the treatment and placebo groups.
Multidimensional scaling using PCoA based on Bray-Curtis distance matrix performed on the iron supplemented (Treatment group) and placebo group did not show that the structure of bacterial communities differs from each other, either for the individual timepoints day 1 (**a**), day 15 (**b**), day 85 (**c**) or for the combined timepoints (**d**). **e.** Volcano Plot using the combined timepoints data identified one species which was statistically different between the two sample groups with a false discovery rate corrected P value < 0.05 (red dot in **e, -log10 FDR < 4**). The black dots indicate species which were not significantly different between the treatment and placebo group. The orange dots indicate species which were different between the treatment (right side) and placebo group (left side) with a minimum log2 fold change of 1 but not with a FDR corrected P value < 0.05. In the day 1 dataset there were 520 samples, in the day 15 dataset there were 412 samples, and in the day 85 dataset there were 457 samples. The combined day 1, day 5, and day 15 dataset contained all 1389 samples across the three sampling time points.

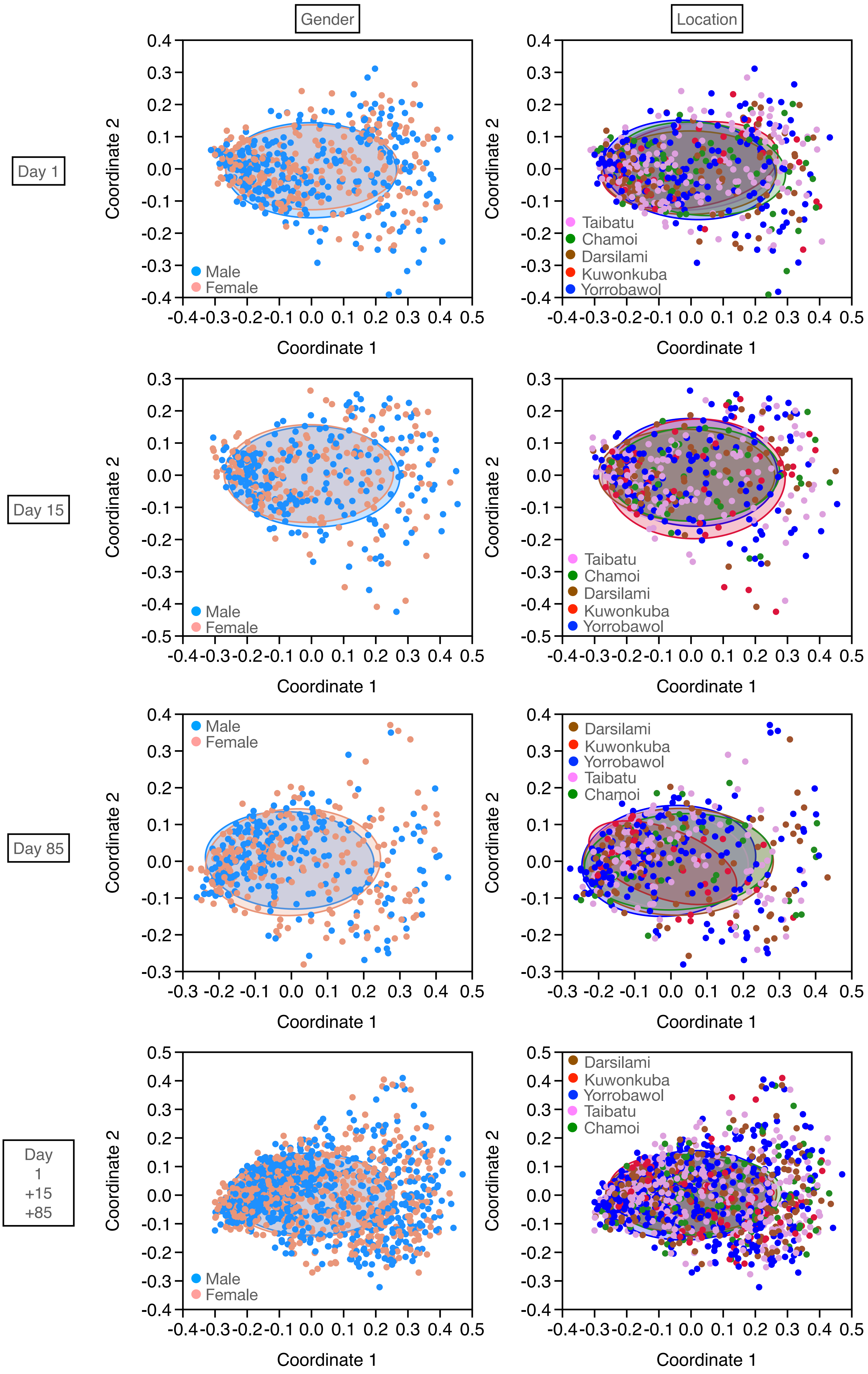

Supplementary Fig. 5 | PCoA for gender and geographic locations.
PCoA based on Bray-Curtis distance matrix performed on gender and geographic locations did not show that the structure of bacterial communities differs between male and female participants and between the five different locations from the samples were collected. In the day 1 dataset there were 520 samples, in the day 15 dataset there were 412 samples, and in the day 85 dataset there were 457 samples. The combined day 1, day 5, and day 15 dataset contained all 1389 samples across the three sampling time points.

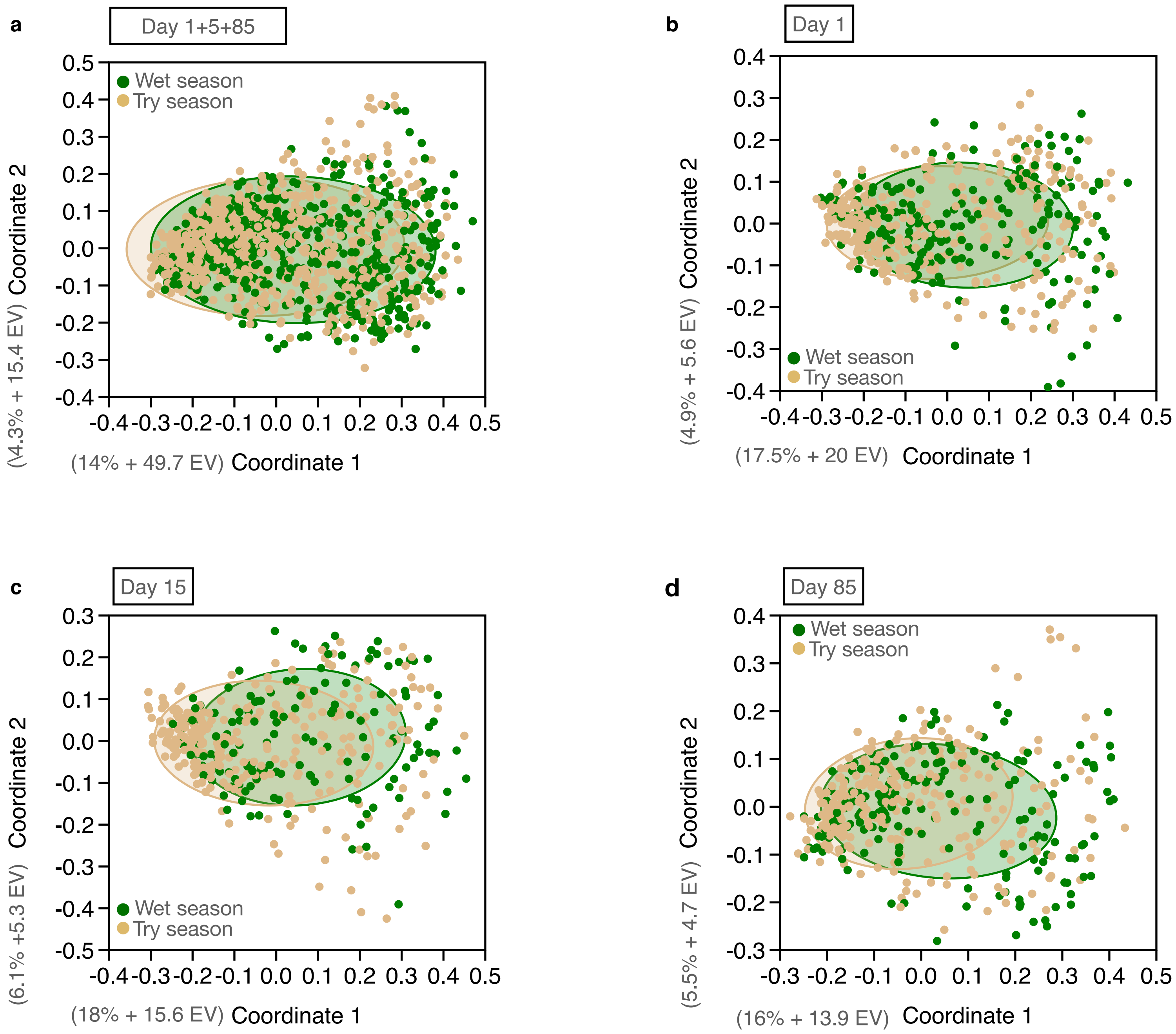

Supplementary Fig. 3 l PCoA analysis for the wet and try season.
Multidimensional scaling using PCoA on Bray-Curtis distance matrix performed on season differences showed some shift in the 60% concentration ellipse between wet and try season for the combined time point dataset (**a**), for day 1 samples (**b**), for day 15 samples (**c**), and for day 85 samples (**d**). In the day 1 dataset there were 520 samples, in the day 15 dataset there were 412 samples, and in the day 85 dataset there were 457 samples. The combined day 1, day 5, and day 15 dataset contained all 1389 samples across the three sampling time points.

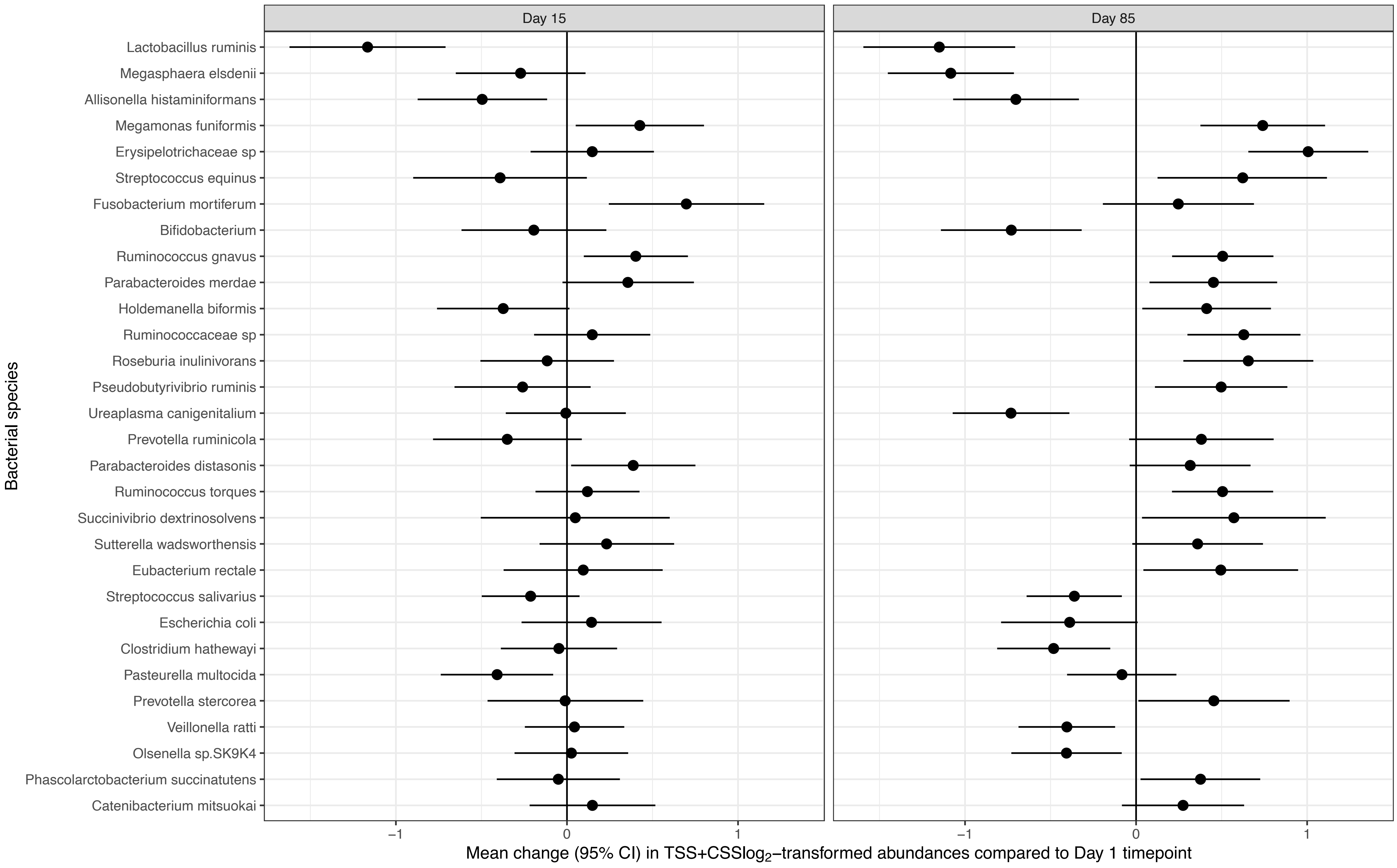

Supplementary Fig. 4 l Mixed-effect linear regression to show bacterial changes across the three different sampling time points. Estimated mean change (points) and 95% confidence interval (lines) in abundances at Day 15 and Day 85 compared to Day 1, obtained from a mixed effects linear regression model accounting for repeat measurements, site and age at enrolment (split into 3 age groups). The top 50 taxa with minimum abundance of 0.2% were used for analysis. Bacterial species with the largest estimated changes are shown. Importantly, no adjustments for multiple comparisons were made to the 95% confidence intervals. In this analysis all 1389 samples from all three time points were used.

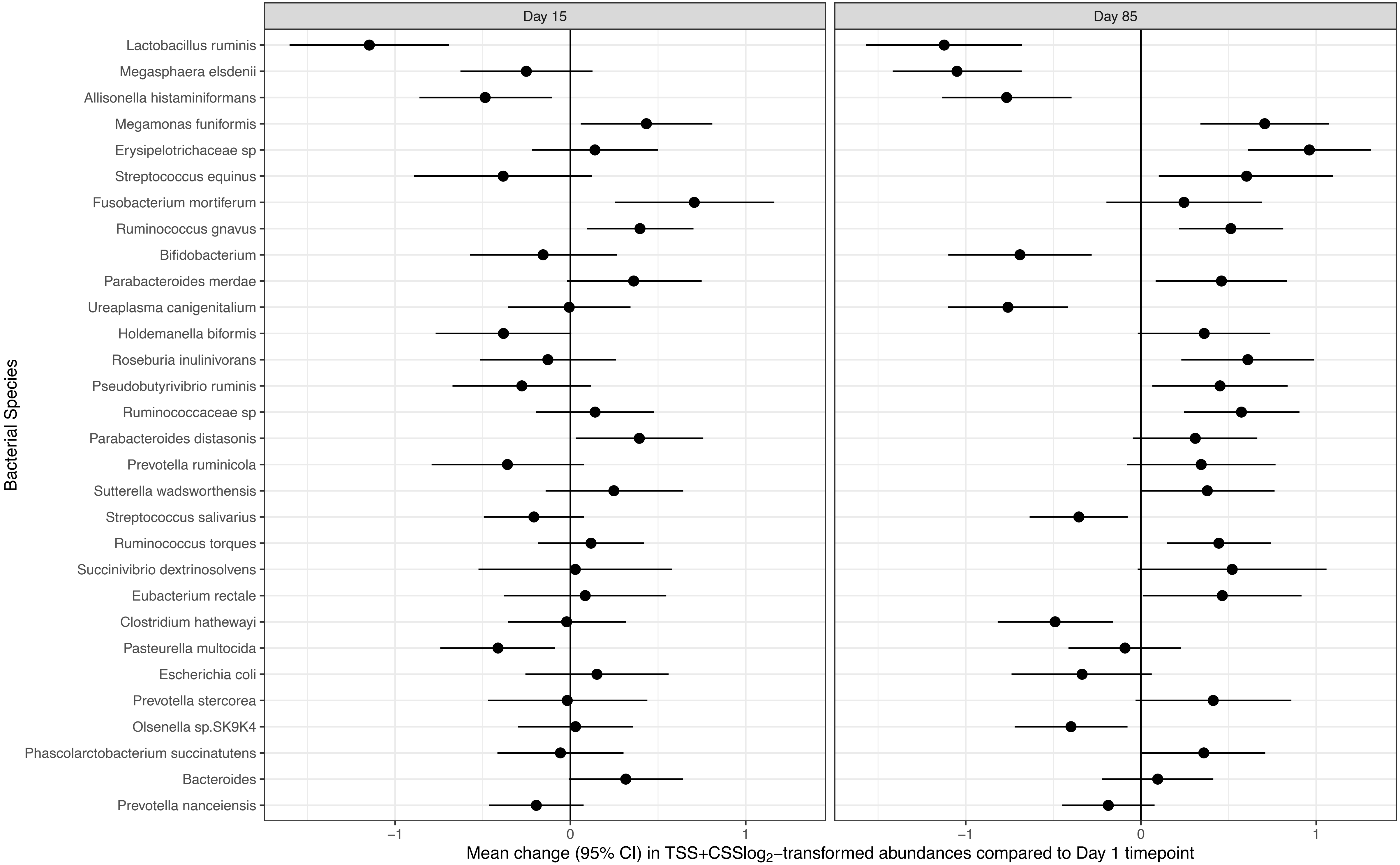

Supplementary Fig. 5 l Mixed-effect linear regression to show bacterial changes across the three different sampling time points.
Estimated mean change (points) and 95% confidence interval (lines) in abundances at Day 15 and Day 85 compared to Day 1, obtained from a mixed effects linear regression model accounting for repeat measurements, site and age at enrolment (split into 11 3-months age groups). The top 50 taxa with minimum abundance of 0.2% were used for analysis. 30 bacterial species with the largest estimated changes are shown. Importantly, no adjustments for multiple comparisons were made to the 95% confidence intervals. In this analysis all 1389 samples from all three time points were used.

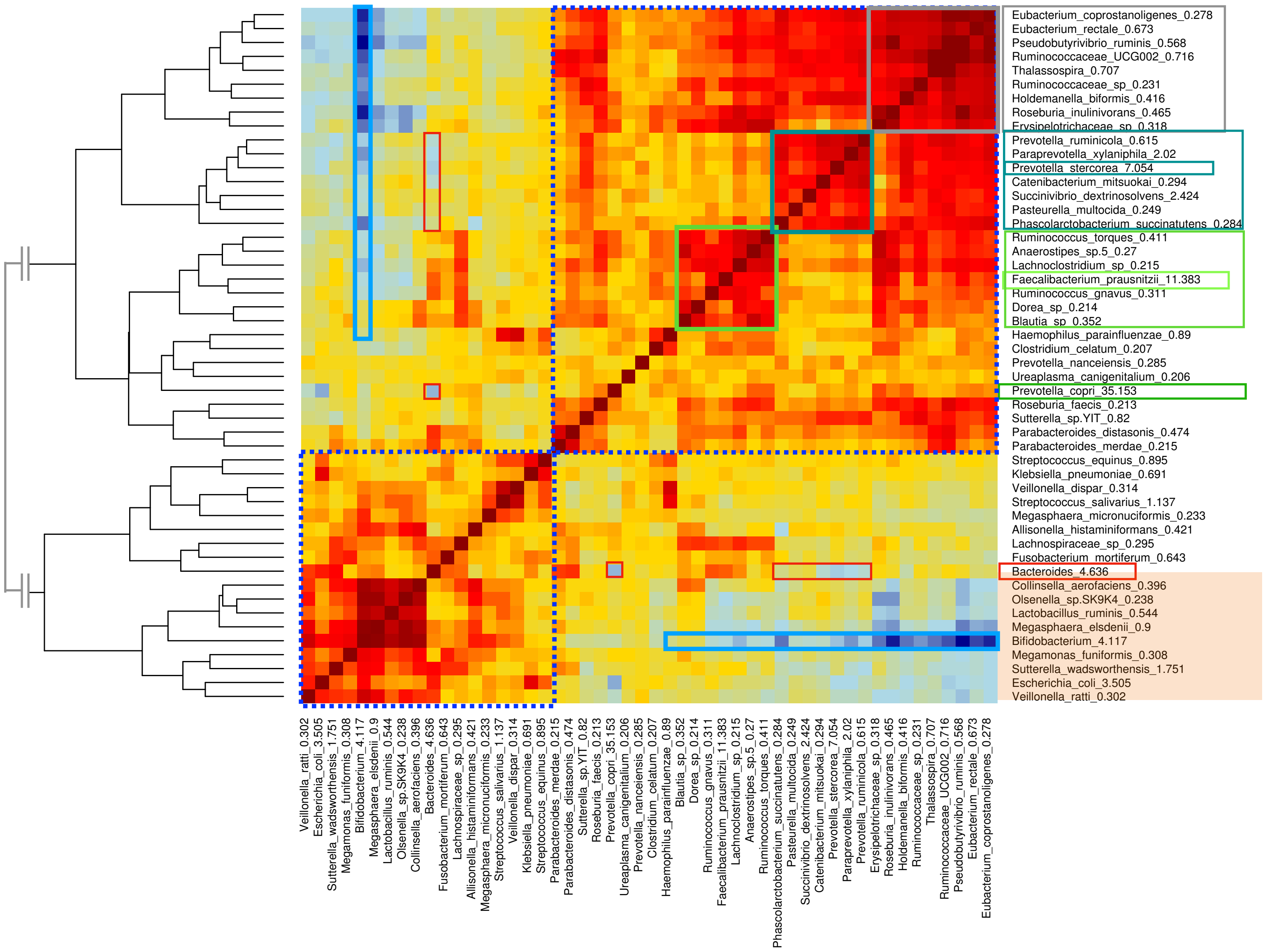

Supplementary Fig. 6 | Taxa association and bacterial trophic networks for Day 1 samples.

A network heat map was generated using the top 50 species with a minimum abundance of 0.2% in all samples. The most dominant clusters identified in the bacterial trophic network correlation analysis are highlighted by different coloured boxes. The *Prevotella stercorea* network is highlighted in teal colour, the *Faecalibacterium prausnitzii* network in light green colour, the *Bifidobacterium* network in orange colour, and an auxiliary group in grey colour. The red heat map colour indicates a strong positive correlation and the blue heat map colour indicates a strong negative correlation. Red boxes denote a negative association between *Prevotella* species and *Bacteroides* and blue boxes denote a negative association between *Bifidobacterium* with other taxa. In the day 1 dataset there were 520 samples.

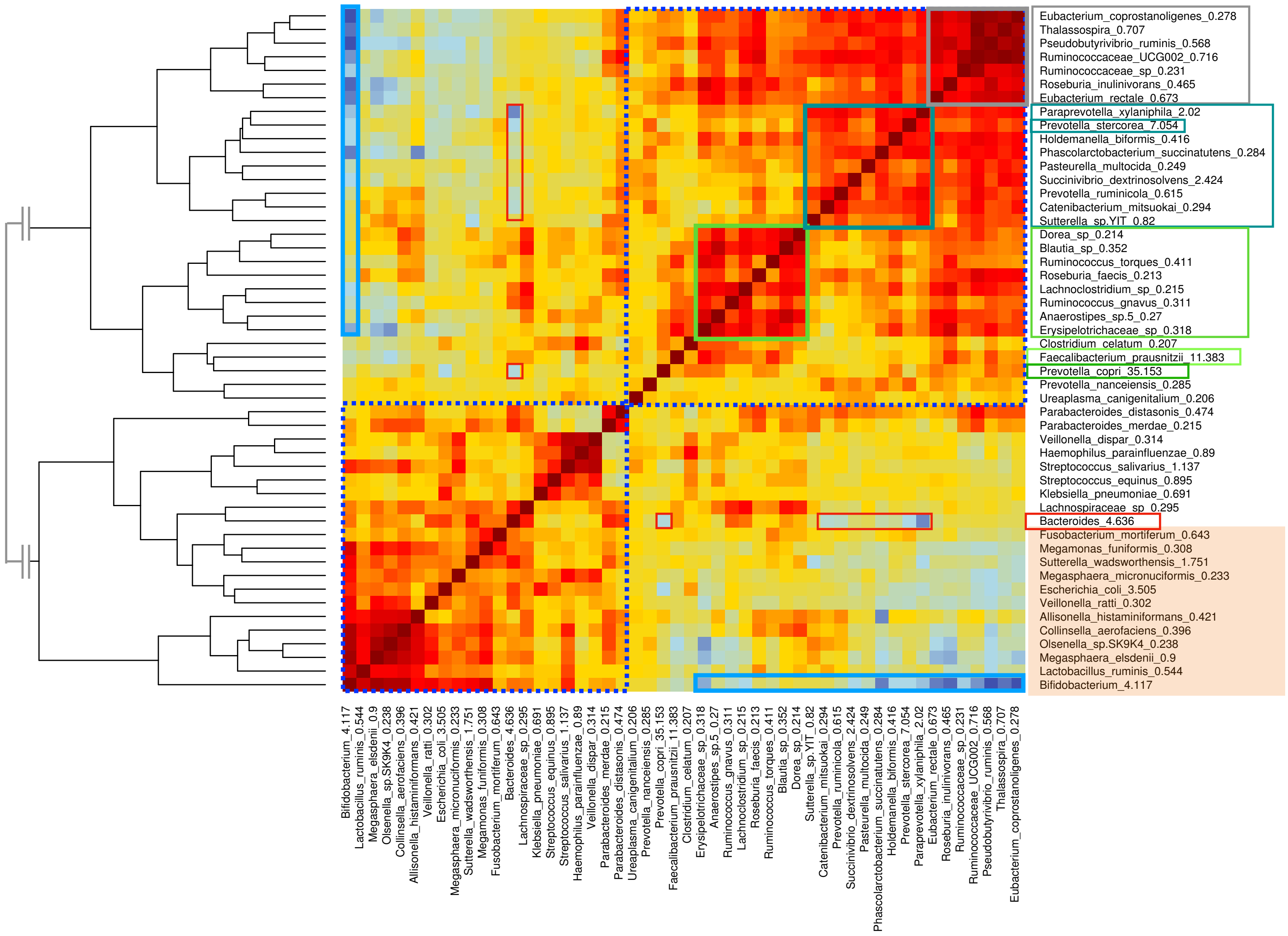

Supplementary Fig. 7 | Taxa association and bacterial trophic networks for Day 85 samples.
A network heat map was generated using the top 50 species with a minimum abundance of 0.2% in all samples. The most dominant clusters identified in the bacterial trophic network correlation analysis are highlighted by different coloured boxes. The *Prevotella stercorea* network is highlighted in teal colour, the *Faecalibacterium prausnitzii* network in light green colour, the *Bifidobacterium* network in orange colour, and an auxiliary group in grey colour. The red heat map colour indicates a strong positive correlation and the blue heat map colour indicates a strong negative correlation. Red boxes denote a negative association between *Prevotella* species and *Bacteroides* and blue boxes denote a negative association between *Bifidobacterium* with other taxa. In the day 85 dataset there were 457 samples.
