# Supplementary material for: Gut microbiomes from Gambian infants reveal the development of a non-industrialized Prevotella-based trophic network
Source: Nat Microbiol. 2021 Dec 31;7(1):132–44. doi: 10.1038/s41564-021-01023-6 (PMC8727306; doi:10.1038/s41564-021-01023-6)
Supplement: Supplementary file 2 — Reporting Summary [file 41564_2021_1023_MOESM2_ESM.pdf]

nature portfolio

Corresponding author(s): Josef Wagner

Last updated by author(s): 2021/11/10

Reporting Summary

Nature Portfolio wishes to improve the reproducibility of the work that we publish. This form provides structure for consistency and transparency in reporting. For further information on Nature Portfolio policies, see our Editorial Policies and the Editorial Policy Checklist.

Statistics

For all statistical analyses, confirm that the following items are present in the figure legend, table legend, main text, or Methods section.

n/a	Confirmed	
☐	☒	The exact sample size (*n*) for each experimental group/condition, given as a discrete number and unit of measurement
☐	☒	A statement on whether measurements were taken from distinct samples or whether the same sample was measured repeatedly
☐	☒	The statistical test(s) used AND whether they are one- or two-sided<br>*Only common tests should be described solely by name; describe more complex techniques in the Methods section.*
☒	☐	A description of all covariates tested
☐	☒	A description of any assumptions or corrections, such as tests of normality and adjustment for multiple comparisons
☐	☒	A full description of the statistical parameters including central tendency (e.g. means) or other basic estimates (e.g. regression coefficient) AND variation (e.g. standard deviation) or associated estimates of uncertainty (e.g. confidence intervals)
☐	☒	For null hypothesis testing, the test statistic (e.g. *F*, *t*, *r*) with confidence intervals, effect sizes, degrees of freedom and *P* value noted<br>*Give P values as exact values whenever suitable.*
☒	☐	For Bayesian analysis, information on the choice of priors and Markov chain Monte Carlo settings
☒	☐	For hierarchical and complex designs, identification of the appropriate level for tests and full reporting of outcomes
☒	☐	Estimates of effect sizes (e.g. Cohen's *d*, Pearson's *r*), indicating how they were calculated

Our web collection on statistics for biologists contains articles on many of the points above.

Software and code

Policy information about availability of computer code

Data collection

The stool samples used in this study were taken from the IHAT-GUT "The Iron Hydroxide Adipate Tartrate" trial (NCT02941081) which is a three-arm, parallel, randomised, placebo-controlled, double-blind study with iron supplementation in young children with mild to moderate iron deficient anaemia. The study population in the IHAT-GUT study were children 6-37 months of age living in the north bank rural communities in the Upper River Region (URR) of The Gambia in West Africa. The study area included 45 villages in the Wuli and Sandu districts, situated approximately 400 km east of the capital Banjul, on the north bank of the river Gambia. All villages had access to borehole tap water at central places and are typical of rural sub-Saharan Africa. A detailed description of the study design, children cohort, recruitment, screening, intervention and ethnic statement are present in Gates open research (Pereira, D. I. A. et al. A novel nano-iron supplement to safely combat iron deficiency and anaemia in young children: The IHAT-GUT double-blind, randomised, placebo-controlled trial protocol. Gates Open Res. 2, 48 (2018)). Stool samples were transferred from lined toilet pot into sterile Omnigene-GUT tubes as soon as possible after the stool was passed. Stool samples were collected at baseline (Day1), at day 15 and at day 85. Stool samples collected in the OMNIgene® GUT tube contain a DNA stabilizing agent that ensures that samples can be kept at ambient temperature for several days. Total stool DNA is extracted from these samples using the Mo Bio PowerLyzer® PowerSoil® DNA Isolation Kit (Qiagen) within 6 weeks of sample collection. All trial data was stored and managed within a clinical database built on the REDCap platform (version 8.9.2), an application specifically designed to collect and store clinical trial data and customised for Electronic Data Capture (EDC) in the field.

Data analysis

1) PAST3 statistical software package, version 3.20
2) Online web portal Calypso version: 8.84
3) Microsoft Excel for Mac version 16.16.14)
4) MOTHUR version between 1.40 and 1.44
5) PRINSEQ version 0.20.4
6) For bacterial 16S alignment we used Silva bacterial database "silva.nr_v123.align" supplied through the Mothur homepage
7) For data plotting we used GraphPad Prism 7.00 for Mac or Mac Numbers (v 6.2.1), and Mac Keynote (v 9.2.1)
8) The ANCOM and ALDEX2 test was conducted in Calypso version 8.84

9) Heatmap for bacterial heatmap analysis was drawn in Calypso version 8.84
10) R studio version 1.2.5042

For manuscripts utilizing custom algorithms or software that are central to the research but not yet described in published literature, software must be made available to editors and reviewers. We strongly encourage code deposition in a community repository (e.g. GitHub). See the Nature Portfolio guidelines for submitting code & software for further information.

Data

Policy information about availability of data

All manuscripts must include a data availability statement. This statement should provide the following information, where applicable:

- Accession codes, unique identifiers, or web links for publicly available datasets
- A description of any restrictions on data availability
- For clinical datasets or third party data, please ensure that the statement adheres to our policy

The metadata and bacterial 16S count data used for the analysis are available in Supplementary Table 6. The raw bacterial 16S sequence data are available from the European Nucleotide Archive (ENA) with the following accession number "ERP110905"

Field-specific reporting

Please select the one below that is the best fit for your research. If you are not sure, read the appropriate sections before making your selection.

☒ Life sciences ☐ Behavioural & social sciences ☐ Ecological, evolutionary & environmental sciences

For a reference copy of the document with all sections, see nature.com/documents/nr-reporting-summary-flat.pdf

Life sciences study design

All studies must disclose on these points even when the disclosure is negative.

Sample size

The sample size was chosen so that the IHAT-GUT study was adequately powered for the first primary objective: determining whether there IHAT was non-inferior to FeSO4 on the day 85 response outcome. It was assumed based on prior evidence that the proportion of children who were responders with FeSO4 at day 85 would be 0.3. The non-inferiority margin was an odds ratio of 0.583 (equivalent to a 0.1 absolute difference in response probability). As any significant result would be tested in a subsequent pivotal (Phase III) study, a 10% one-sided type I error rate was used. A sample size of 200 per arm provides 89% power to demonstrate non-inferiority when the two arms have the same response probability.
As described further in protocol, the sample size of 200 per arm also provides: 1) 90% power (10% one-sided type I error rate) for testing superiority of IHAT over FeSO4 for prevalence of diarrhoea when prevalence is 0.15 in IHAT arm and 0.25 in FeSO4 arm; 2) 93% power (10% one-sided type I error rate) for testing non-inferiority of IHAT vs placebo for diarrhoea prevalence when it is 0.15 in the IHAT and placebo arms with a 0.1 absolute non-inferiority margin; 3) 90% power (10% one-sided type I error rate) to find reduction in incidence density of diarrhoea in IHAT vs FeSO4 assuming 1.28 episodes per child over the 85 days in the FeSO4 arm and rate ratio of 0.8.
For the secondary outcomes, the trial (n=200 per arm) would have over 85% power to detect significant differences between all the arms in terms of enterobacteria, NTBI and calprotectin.
To account for an anticipated 15% non-completion rate, based on previous studies in The Gambia, the target sample size was set to 705.

Data exclusions

Children included in the study had to meet all the inclusion criteria and none of the exclusion criteria at the time of screening to take part in the study.
Inclusion criteria were:
Apparently healthy; 6–35 months old; free of malaria; with IDA defined as 7≤ Hb <11 g/dl and ferritin <30 μg/l; resident in the study area (and planning to remain in the study area for the duration of the trial); able and willing to comply with the study protocol; informed consent given by parent.
Exclusion criteria for the study were:
Congenital anomalies/birth defects (except minor external congenital malformation); severe malnutrition (z-scores for length/height-for-age (HAZ), weight-for-age (WAZ), weight-for-length/height (WHZ) <-3 standard deviations (SD); shock syndrome; chronic conditions; sickle cell and thalassaemia; currently participating in another study; currently taking iron supplements/multiple micronutrient supplements; currently experiencing moderate-severe diarrhoea, defined as those diarrhoea episodes where (i) the child passes more than five loose or watery stools per day, (ii) there is blood in the stool (dysentery), or (iii) the child shows signs of clinical dehydration (assessed by the study nurse based on physical signs such as little or no urination, sunken eyes, and skin that lacks its normal elasticity).

For the bacterial 16S analysis following additonal data exclusion criteria are reported in the method section: Participant exclusion
Children with severe malnutrition (N=88, 6% of children who were screened) (z-scores for length/height-for-age (HAZ), weight-or-age (WAZ), weight-for-length/height (WHZ) -3 standard deviations (SD) were excluded from the trial. Mean z-scores for the included children were around -1. Data which failed the high-quality control procedure in the bioinformatics pipeline were also excluded so any samples with low amount of DNA from which no reads >1000 were obtained. This excluded 61/1466 samples and an additional small number of 16 samples from 15 patients who received antibiotics were also removed leaving 1389 samples from 633 patients for detailed analysis. Antibiotic treatment affected 15 Day 85 samples and one Day 15 sample. Therefore, not all children in the IHAT-GUT trial were used for this study.

Replication

The stool samples and associated bacterial 16S data were obtained from a large paediatric clinical trial conducted in rural The Gambia, West Africa. This is a double-blinded randomised placebo-controlled study adequately powered for all primary and secondary endpoints. All procedures were carried out following study specific SOPs and all staff were trained appropriately. All deviations were recorded. Analysis of endpoints followed a Statistical Analysis Plan finalised before unblinding the trial. Quality control and assurance was applied throughout the

	trial. The trial will be conducted in accordance with the principles of GCP as laid down in the Consolidated Guideline for Good Clinical Practice published by the International Conference on Harmonization in 1996 (ICH GCP Guideline) and the MRC Unit The Gambia at the London School of Hygiene & Tropical Medicine will Sponsor the research. The trial has not been replicated.
Randomization	Randomisation was performed using a stratified block design to achieve group balance in terms of age (6-11 months, 12-23 months and 24-37 months) and baseline haemoglobin concentration (above and below median, calculated for each cohort separately) at the pre-enrolment day (Day 0). Within each of the 6 resulting strata, children were randomly assigned to one of the three study treatment arms (1:1:1 ratio) using a computer program written by the trial statistician and a block randomisation approach with fixed block size of six was used.
Blinding	All participants and the entire study team, including lab staff, outcome assessors and database analysts were blinded to which treatment group participants belonged to. Each treatment dose (iron compounds and placebo) was encapsulated in identical capsules (also containing powders of identical colour) by Capsugel-Lonza (Ploermel, France) and the supply of capsules for each child was packed in one bottle, individually labelled with the randomisation number/study ID for each child. If emergency unblinding was required, only the particular study subject in question would have been unblinded, since each participant had a unique treatment code. For bacterial 16S analysis the data were not blinded because the group informations were necessary for analysis.

Reporting for specific materials, systems and methods

We require information from authors about some types of materials, experimental systems and methods used in many studies. Here, indicate whether each material, system or method listed is relevant to your study. If you are not sure if a list item applies to your research, read the appropriate section before selecting a response.

Materials & experimental systems

n/a	Involved in the study
☒	☐ Antibodies
☒	☐ Eukaryotic cell lines
☒	☐ Palaeontology and archaeology
☒	☐ Animals and other organisms
☐	☒ Human research participants
☐	☒ Clinical data
☒	☐ Dual use research of concern

Methods

n/a	Involved in the study
☒	☐ ChIP-seq
☒	☐ Flow cytometry
☒	☐ MRI-based neuroimaging

Human research participants

Policy information about studies involving human research participants

Population characteristics	The study population in IHAT-GUT was children under the age of 3 years inhabiting the north bank rural communities in the Upper River Region (URR) of The Gambia in West Africa. The URR has a population of approximately 200,000, with only one major town, Basse; it is otherwise typical of rural sub-Saharan Africa. The study area included 45 villages in the Wuli and Sandu districts, situated approximately 400 km east of the capital Banjul, on the north bank of the river Gambia. All villages had access to borehole tap water at central places.<br>Prospective participants (children 6-35 months of age) were identified through data collected by the study field team in the 45 study villages.<br>Inclusion criteria:<br>- Age 6-37 months<br>- Apparently healthy with no signs of acute infection<br>- Free of malaria (RDT negative)<br>- Height-for-age (HAZ), weight-for-age (WAZ) and weight-for-height (WHZ) Z scores greater than -3 standard deviations (SD).<br>- IDA defined as 7≤ Hb <11 g/dl AND ferritin<30 µg/L, as per WHO recommendation on assessing iron status for children under 5y that live in regions with high infection burden<br>- Resident in the study area (and planning to remain in the study area for the duration of the trial)<br>- Ability and willingness to comply with the study protocol (daily intake of supplement and daily study visits with weekly finger prick)<br>- Informed consent given by parent or guardian.<br>48% of the participants were female.
Recruitment	Prospective participants (children 6-37 months of age) were identified through data collected by the study field team in the 45 study villages.<br>The field team visited the parents of all young children identified as prospective participants to explain the study and answer any questions they may have. Those interested in taking part in the study were then invited to attend a screening visit at one of the five study health facilities.<br>Yorrobawol health center, Darsilami community health post, Konkuba community health post, Taibatu health post and Chamoi Health Center.<br><br>At screening, the child was examined by a study nurse or clinician. To be eligible for the study, participants had to meet all the following inclusion criteria and none of the exclusion criteria.<br>We enrolled children in 3 cohorts (target n=235 children each) that were run sequentially.<br>Children were allocated to one of the study health facilities according to the child's home proximity to each of the 5 study health facilities (target max. 60 children seen at each health facility). For logistical reasons, children allocated to one health facility had all their study visits (including weekly check-ups) on the same day of the week (for example, Yorrobawol health

centre had all study visits on Mondays).
The recruitment period for each of the three cohorts was planned to be approximately 1 month prior to enrolment into each of the sequential cohorts, with expectations to screen 50 children/day for 2 weeks, and the following 2 weeks to get all screening results back from the lab. Eligible children were invited for a pre-enrolment day back at the clinic (Day 0), for a finger prick to confirm absence of malaria and that haemoglobin was still within the inclusion range. Those confirmed eligible were then randomised and enrolled in the study.

A total of 1494 participants were screened and 642 participants met the eligibility criteria for entry into the trial and were randomised after the day 0 visit (n=214 per study group). No bias were identified that could impact on the results.

Ethics oversight

The trial was conducted in accordance with the ethical principles that have their origin in the Declaration of Helsinki, and that are consistent with the International Conference on Harmonisation (ICH) requirements for Good Clinical Practice (GCP), and the applicable regulatory requirements. The study sponsor was the London School of Hygiene and Tropical Medicine (LSHTM) and the study was conducted at the Medical Research Council (MRC) Unit The Gambia at LSHTM (MRCG). Scientific advice on the study protocol has been given by the UK Medicines and Healthcare products Regulatory Agency (MHRA 1400, 21/12/2016). The study protocol and any subsequent amendments have been reviewed and approved by The Gambia Government/MRC Joint Ethics Committee (reference SCC1489). Clinical Trials Authorisation has been granted by the Medicines Control Agency, The Gambia (HP373/347/16/MJK(80)).

Note that full information on the approval of the study protocol must also be provided in the manuscript.

Clinical data

Policy information about clinical studies

All manuscripts should comply with the ICMJE guidelines for publication of clinical research and a completed CONSORT checklist must be included with all submissions.

Clinical trial registration

This trial is registered at clinicaltrials.gov (NCT02941081).

Study protocol

Pereira, D. I. A. et al. A novel nano-iron supplement to safely combat iron deficiency and anaemia in young children: The IHAT-GUT double-blind, randomised, placebo-controlled trial protocol. Gates Open Res. 2, 48 (2018).

Data collection

The trial was conducted from November 2017 to November 2018 in The Gambia
Study samples are collected at one of the 5 study clinical facilities, Yorrobawol health center, Darsilami community health post, Konkuba community health post, Taibatu health post and Chamoi Health Center, and transported to the study laboratory in Basse for sample processing and analysis, and from there to other laboratories in Fajara, Banjul for further processing and analysis.

Outcomes

The study was a phase II trial designed to investigate for the first time the efficacy and tolerability of daily supplementation with IHAT in young children. This involved investigation of four primary objectives:
1) non-inferiority of IHAT compared to ferrous sulphate in terms of iron deficiency anemia correction: this involved comparing treatment response, defined as correction of iron deficiency;
with achievement of either a normal Hb (>=11 g/dL) or an increase of at least 1 g/dL from baseline (day 1) to day 85 of iron supplementation;
2) superiority of IHAT compared to ferrous sulphate in terms of incidence density of moderate-severe diarrhoea;
3) superiority of IHAT compared to ferrous sulphate in terms of prevalence of moderate-severe diarrhoea;
4) non-inferiority of IHAT compared to placebo in terms of prevalence of moderate-severe diarrhoea.
Thus there were four primary endpoints: composite of iron deficiency at day 85 and haemoglobin level at day 85 (combined into the treatment efficacy response endpoint), incidence density of diarrhoea (i.e. number of new moderate-severe diarrhoea episodes per child over the 85 days intervention) and prevalence of diarrhoea (proportion of children with at least one episode of moderatesevere diarrhoea over the 85 days intervention).
Secondary endpoints were faecal microbiome diversity and profile, abundance of enteric pathogens, faecal calprotectin, hospitalisation and morbidity, malaria infection, treatment failures (i.e. the number of children who have to stop the study because their Hb falls below 7 g/dL), the proportion of days a child has diarrhoea over the 85 days intervention period ('longitudinal prevalence' of diarrhoea), the proportion of days a child has moderate-severe diarrhoea over the 85 days period ('longitudinal prevalence' of moderate-severe diarrhoea), incidence density of bloody diarrhoea (i.e. the number of bloody diarrhoea episodes per child-month of observation), markers of systemic inflammation (serum CRP and AGP), and systemic markers of iron handling (hepcidin, sTfR, transferrin saturation and circulating non-transferrin bound iron - NTBI).
sTfR and hepcidin were assessed at days 1 and 85 and all other outcome measures were assessed at days 1, 15 and 85.
