# Supplementary material for: Gut microbiomes from Gambian infants reveal the development of a non-industrialized Prevotella-based trophic network
Source: Nat Microbiol. 2021 Dec 31;7(1):132–44. doi: 10.1038/s41564-021-01023-6 (PMC8727306; doi:10.1038/s41564-021-01023-6)
Supplement: Supplementary file 3 — Peer Review Information [file 41564_2021_1023_MOESM3_ESM.pdf]

natureresearch

Peer Review Information

Journal: Nature Microbiology
Manuscript Title: Gut microbiomes from Gambian infants reveals the development of a non-industrialised Prevotella-based trophic network
Corresponding author name(s): Josef Wagner

Reviewer Comments & Decisions:

Decision Letter, initial version:

Dear Josef,

Thank you for your patience while your manuscript "The gut microbiome of 7-37 month old children from The Gambia shows the development of a distinct non-industrial Prevotella-based trophic network" was under peer-review at Nature Microbiology. It has now been seen by 4 referees, whose expertise and comments you will find at the end of this email. Although they find your work of some potential interest, they have raised a number of concerns that will need to be addressed before we can consider publication of the work in Nature Microbiology.

In particular, all referees have concerns with the bioinformatics and statistical analyses currently being used including the need to account for repeated measures and multiple comparisons (referees #2 and #4), using alternative approaches for the trophic network and cluster analysis (referees #1 and #2), provide statistical support for clusters and associations (referees #2 and #4) and referee #4 suggests using MaAsLin2 or the random forest approach to look at what species change over time. Referee #4 suggests stratifying samples by age group and looking at other factors, other than age, that impact the gut microbiome, referee #1 suggests identifying potential pathogens, and referee #3 suggests looking at Prevotella subspecies if the dataset allows. Referees #3 and #4 both suggest comparing the current dataset to existing datasets from industrialised populations and we feel that this would strengthen the manuscript.

Should further experimental data allow you to address these criticisms, we would be happy to look at a revised manuscript.

We are committed to providing a fair and constructive peer-review process. Please do not hesitate to contact us if there are specific requests from the reviewers that you believe are technically impossible or unlikely to yield a meaningful outcome.

We strongly support public availability of data. Please place the data used in your paper into a public data repository, if one exists, or alternatively, present the data as Source Data or Supplementary Information. If data can only be shared on request, please explain why in your Data Availability Statement, and also in the correspondence with your editor. For some data types, deposition in a public repository is mandatory - more information on our data deposition policies and available repositories can be found at https://www.nature.com/nature-research/editorial-policies/reporting-standards#availability-of-data.

Please include a data availability statement as a separate section after Methods but before references, under the heading "Data Availability”. This section should inform readers about the availability of the data used to support the conclusions of your study. This information includes accession codes to public repositories (data banks for protein, DNA or RNA sequences, microarray, proteomics data etc…), references to source data published alongside the paper, unique identifiers such as URLs to data repository entries, or data set DOIs, and any other statement about data availability. At a minimum, you should include the following statement: “The data that support the findings of this study are available from the corresponding author upon request”, mentioning any restrictions on availability. If DOIs are provided, we also strongly encourage including these in the Reference list (authors, title, publisher (repository name), identifier, year). For more guidance on how to write this section please see:
http://www.nature.com/authors/policies/data/data-availability-statements-data-citations.pdf

If revising your manuscript:

* Include a “Response to referees” document detailing, point-by-point, how you addressed each referee comment. If no action was taken to address a point, you must provide a compelling argument. This response will be sent back to the referees along with the revised manuscript.

* If you have not done so already we suggest that you begin to revise your manuscript so that it conforms to our Article format instructions at http://www.nature.com/nmicrobiol/info/final-submission. Refer also to any guidelines provided in this letter.

* Include a revised version of any required reporting checklist. It will be available to referees (and, potentially, statisticians) to aid in their evaluation if the manuscript goes back for peer review. A revised checklist is essential for re-review of the paper.

When submitting the revised version of your manuscript, please pay close attention to our href="https://www.nature.com/nature-research/editorial-policies/image-integrity">Digital Image Integrity Guidelines. and to the following points below:

-- that unprocessed scans are clearly labelled and match the gels and western blots presented in figures.
-- that control panels for gels and western blots are appropriately described as loading on sample processing controls
-- all images in the paper are checked for duplication of panels and for splicing of gel lanes.

Finally, please ensure that you retain unprocessed data and metadata files after publication, ideally

archiving data in perpetuity, as these may be requested during the peer review and production process or after publication if any issues arise.

Please use the link below to submit a revised paper:

{redacted}

Note: This url links to your confidential homepage and associated information about manuscripts you may have submitted or be reviewing for us. If you wish to forward this e-mail to co-authors, please delete this link to your homepage first.

Nature Microbiology is committed to improving transparency in authorship. As part of our efforts in this direction, we are now requesting that all authors identified as 'corresponding author' on published papers create and link their Open Researcher and Contributor Identifier (ORCID) with their account on the Manuscript Tracking System (MTS), prior to acceptance. This applies to primary research papers only. ORCID helps the scientific community achieve unambiguous attribution of all scholarly contributions. You can create and link your ORCID from the home page of the MTS by clicking on 'Modify my Springer Nature account'. For more information please visit please visit www.springernature.com/orcid.

If you wish to submit a suitably revised manuscript we would hope to receive it within 6 months. If you cannot send it within this time, please let us know. We will be happy to consider your revision, even if a similar study has been accepted for publication at Nature Microbiology or published elsewhere (up to a maximum of 6 months).

In the meantime we hope that you find our referees' comments helpful.

{redacted}

****
Reviewer Expertise:

Referee #1: tropical gastroenterology, infant gut infections and microbiota, clinical trials
Referee #2: gut microbiome, industrialisation
Referee #3: gut microbiome, Prevotella, bioinformatics
Referee #4: infant microbiome, bioinformatics

Reviewer Comments:

Reviewer #1 (Remarks to the Author):

The gut microbiome of 7-37 month old children from The Gambia shows the development of a distinct non-industrial Prevotella-based trophic network
De Goffau et al

This manuscript presents data from a large substudy of an iron supplementation trial in The Gambia, which characterise the development of the microbiome in young children. The study provides

normative profiles of maturation of the gut microbiota in rural West Africa, and as such will be very valuable to many researchers over the coming years. The main conclusion is that age is the dominant influence on microbiota composition, with no evidence of an effect of nutritional status (though very malnourished children were excluded, so the power of this statement is somewhat dimnished). The microbial communities present were divided into four groups: Prevotella copri, the Prevotella stercorea group, the Faecalibacterium prausnitzii group, and unclassified/other. The authors identify the Prevotella stercorea group as a group rarely found in microbiomes from industrialised countries and conclude that this group is the archetype "good" microbiome cluster present in rural African children. This is an important study, well-powered and robustly analysed, which puts forward a very interesting way of looking at geographical and diet-related microbiome variation.

Major comments
1 The title is a bit clumsy; "non-industrial" does not make grammatical sense unless referring to non-industrial society or non-industrial diet. Please re-phrase.

2 In the Introduction the authors introduce the "enterotype" hypothesis, but then ignore this intriguing question and leave the reader somewhat dissatisfied that they do not in the end come off the fence and decide whether their data do or do not support this hypothesis. In my view their data could be used to refine the enterotype hypothesis into a more sophisticated classification. Do they agree?

2 I was left wondering what criteria were used to define "trophic network clusters". In lines 410 ff there is a claim that the Bifidobacterium group is not a real "trophic cluster" but no evidence is provided to refute its definition as such. This term needs to be defined and its application justified when used to decide which are "real" trophic communities.

3 Pathogens were not specifically identified as the analysis used only 16S sequencing. Is it fair to comment on "potential" pathogens without data?

4 Figures 5 and 6 are central to the argument of this paper. However, Figure 6e does not directly show the inverse correlation between the P. stercorea group and Bacteroides which is prominent in the Discussion (lines 533 ff). This should be shown directly rather than just as the subtraction for PC2.

Minor comments
5 Abstract: percentages shown are % of bacterial sequences, or % of children carrying them? Please clarify.

6 Presumably length was measured in children under 2.

7 How was antibiotic use ascertained? Online methods section 4.8 suggests (but does not state explicitly) that this was just from clinic records. How can you be sure that over-the-counter antibiotics did not confound the analysis?

8 Spelling errors: funiformis (line 189); PERMANOVA (several instances read PERMANOA)

9 P=0.0099 should be shortened to P=0.01

10 Table 2 has no legend; the different measures (eg Chao1) should be explained. Some explanation/justification of Fisher's is included in Methods, but it would be helpful to explain the

measures used in the main text so that their significance is apparent to the reader.

11 Figure 3 legend has (a) and (b) but only one panel is shown.

12 Figure 5 should go before Supp Fig 4

13 What were the criteria for inclusion in the boxes in Figure 5? Why were Megamonas or Sutterella not included in the Bifido box (variously described as black or grey)?

14 Online methods (Line 290) refers to the heatmap in Figure 4a; this should be 5a.

Reviewer #2 (Remarks to the Author):

Overall, I found this study to be of extreme interest and well carried out. However, there are a few issues that should be addressed:

1. Using Spearman or Pearson correlations to build trophic networks based on 16S abundances is problematic, since these methods result in many falsely positive erroneous network links (see Friedman & Alm 2012; Weiss et al. 2016). Filtering out lowly abundant sparse taxa, as the authors do, is good, but the analysis is still problematic. Considering this is time series data, Local Similarity Analysis might be more appropriate. (See Figure 7 of Weiss et al. 2016). Other programs, such as FastSpar (fast implication of SparCC) would be admissible. Further, determining groups of correlated taxa by drawing boxes by observation is fine, but a more statistically robust approach would be to estimate the number of clusters using an algorithm such as the gap statistic.

2. Authors demonstrate that b-diversity between iron supplementation and placebo was not different. What about a-diversity? Similar question for the abx treated children. Since these samples are then all lumped together for subsequent analysis, which includes looking at a-diversity, it is important to establish how these factors (iron supplementation and abx treatment) affected microbiome a-diversity.

3. I didn't see how authors dealt with longitudinal samples. Are they correcting for resampling of the same individuals in any way, specifically in their PERMANOVA and Spearman tests (for PCo correlations)?

4. Is the Simpson's index shown in Fig 2b significant between each pairwise comparison? Not clear from text which comparisons are significant.

5. Similarly, in other diversity tests demonstrating significance, is this only between first and last time point groups? Between each group? Not clear from text.

6. These statements seem to contradict one another:
"P. copri and the P. stercorea network are negatively correlated with Bacteroides
whilst the F. prausnitzii network is positively correlated with Bacteroides."

“PC4 in Figure 6.f indicates that P.copri and the F. prausnitzii network are positively correlated with one another”
Given the negative correlation between prevotella and bacteroides, how can both be positively correlated with F. prausnitzii?

Minor issues:

- Line 153: Given that age is an important feature in the PCoA, how was uneven group sizes accounted for the age bins?

- It would be helpful if the authors described their definition of species. Are ASVs/OTUs agglomerated at the species level? If so, it could present a major issue, since all non-assigned taxa will be assigned to NA or thrown out (depending on parameters chosen). Either way, they will contribute in a statistically inadmissible way or not at all to multivariate analyses. Also, given that a population like that in rural Gambia is relatively unexplored, by binning NAs or removing them, diversity could be missed.

- The text for Fig 3 states that a Kruskal Wallis test on ranks (line 294) was used but the figure says ANOVA in the panels.

- I don’t see an A and B in figure 3. Is the legend correct?

- Line 545 referring to an “African” microbiome is very general, which cohort and reference are being referred to here?

Reviewer #3 (Remarks to the Author):

De Goffau and Jallow et al., Investigate the microbiome structures of 616 rural infants from Gambia. The topic of the developing infant microbiome has been an area of intense research but mainly focused on industrialized populations. Pre-industrial populations, and how the infant gut microbiota develops is therefore of interest. The authors confirm that in pre-industrial populations Prevotella are likely a key component of the microbial communities in the gut.

Major points
In the introduction the concept of gut enterotypes is introduced and the authors note that the concept is debated in the field. Adding to this debate, it has been suggested that the Prevotella/bacteroides divide is an artefact of relative abundancies: DOI: 10.1038/nature24460. For completeness this could also be added to the introduction. In addition, such findings will have implications on the correlative associations observed (figure 5) by the authors and would be worth discussing in this context also.

Lines: 135-138. In the description of the cohort, while you mention malnutrition and as exclusion criteria. Regarding health of participants there is no mention of intestinal parasites. With rural pre-industrial populations, it is likely that infection is common and thus intestinal inflammation, which could be relevant when looking at the microbiota. Was any information regarding intestinal parasitism

and inflammatory status collected?

Additionally, and sorry if I missed this, but I do not see the metadata associated with each participant in the study as supplementary material. Only a summary of the cohort in Table 1. Such information would be useful for other researchers in meta-analysis investigations.

Development of the early life microbiome has been the focus of research attention for a number of years. The opportunity from a pre-industrial population is to find parallels and contrast with that of industrialized populations. There is a limited comparison to other cohorts, the manuscript would be strengthened by placing the Gambia cohort in the context of existing literature, both from industrialized and pre-industrialized populations. For instance pre-industrialized doi.org/10.1038/s41598-019-49274-y; doi.org/10.1111/ppe.12623; DOI: 10.1016/j.celrep.2018.05.018

Line 556-569: The authors mention the P. copri abundance in their cohort emphasizes its importance in fermenting complex carbohydrate. While it is true P. copri utilizes carbohydrates, the benefit of P. copri, particularly in association with glucose metabolism, has received contradictory reports as to whether it is beneficial or not. Also, Bacteroides which are believed to be excluded by Prevotella and dominate industrialized microbiomes are also known fermenters and have a large number of carbohydrate utilization pathways. This highlights that the intestinal dynamics, industrialization and shifts in diet are complex and could be mentioned in the discussion.

A finding of this study is the abundance of Prevotella and particularly P. copri in the infant microbiome. The substantial sub-species diversity of P. copri over the past few years has become increasingly evident. The resolving power of 16S is limited but did you look for and identify sub species types for the Prevotella species?

Probably a simple oversight, but certain statements in the manuscript should be backed-up by references. This should be checked throughout the manuscript. For example

Line 410: "These positive associations do not seem to represent a trophic network except possibly for the association of Bifidobacterium (which produces lactate)" reference(s) needed

Line 411 "Megasphaera elsdenii (which metabolizes lactate and produces butyrate)." Reference(s) needed

Line 542: "Similar to infant microbiotas in industrialized countries, the Bifidobacterium genus is the most important and dominant group early in infancy" Reference(s) needed.

Minor points
Lines 151: At the opening of the Results section effort has been taken to demonstrate that antibiotic usage has little to no consequences on the samples from those participants. Based on the relatively small number of participants receiving antibiotics (2.4%), I wonder if it is not simpler and cleaner to remove those subjects?

Alpha diversity: Figure 2 is dedicated to the changes in alpha diversity. Other than presenting the changes in the results these analyses, and significance thereof are not commented on in the discussion?

Line 215: Reference to the cohort as patients. Wouldn’t participants be a more fitting term?

Figure 1: Check Plot axes for formatting errors

Reviewer #4 (Remarks to the Author):

The manuscript by De Goffau & Jallow and colleagues present faecal microbiome data collected from month 7-37 of life from subjects in the The Gambia. The original study aimed to investigate whether children receiving iron supplementation differed from the placebo group. However, as no differences between groups were observed, the authors instead conducted a more descriptive analysis of gut microbiome development over time in African children. Given the unique cohort, this is still a worthy pursuit and I was pleased to see authors from The Gambia included on the publication. My major concerns with the analysis relate to the way the different statistical tests have been performed, which have an inconsistent angle of age/time, and in many cases an apparent failure to account to repeated measures. I also feel the manuscript lacks a clear narrative and would greatly benefit from a critical rewrite and focusing of the main messages. I expand on these and other points below which I hope the authors will find helpful.

At 27 pages, the results section is very long and at times lacks a clear narrative. This is further complicated as throughout the results the authors split the data into various different time groupings, such as “age at enrolment”, “timepoint”, and “age in months”. It can be difficult to follow why samples have been split as they have and what the different way of presenting the data adds. The manuscript would improve drastically with a critical rewrite of the results to make it more focused and with a constant narrative throughout.

The introduction reads more like a discussion in parts and could be improved to give a broader overview that is relevant to the current study. Currently it focuses primarily on 1) one study by Yatsunenko and 2) the concept of enterotypes, neither of which set the scene for why the current study is important.

How did the authors account for repeated measures in their analysis? For example, in Figure 1 box plots and the supporting PERMANOVA test, would more than 1 sample from a given subject appear within the same age group? If so, a PERMANOVA test, which assumes cross-sectional study design, would not be a valid approach. Similarly for analysis in Figures 3, 4, and throughout, how were multiple comparisons dealt with?

Only P values adjusted for multiple comparisons should be presented. Mention of “significant” features before adjusting for multiple comparisons should be avoided.

In the analysis looking at the different time points (e.g., lines 212 – 232) the authors include all subjects, regardless of age at enrolment. I am not sure this is the best approach, as one can expect a vastly different gut microbiome between the three age categories based on many factors, most notably diet (specifically whether they are breastfed or not). The amalgamation of the different age ranges might explain why so few species were found to differ between the different time points? Do

the authors find a consistent result if the analysis is stratified by the previously used “age at enrolment” subgroups?

The authors state “age was the only contributing factor which differentiated the gut microbiomes of samples in this cohort”. However, it is not clear what this is based on and what other “factors” were tested. As this paper is largely descriptive, it would be beneficial to perform an analysis of all factors of interest and determine what explains the most variance / what is most significant (e.g., using adonis or envfit). Given the well reported impact of factors such as subjectID, birth mode, and diet on gut microbiome development during infancy/childhood of industrialised infants, similar analysis from the current non-industrialised population would serve as a nice comparator.

It is not clear why the 11 age groups were created and why the Kruskal-Wallis rank test was used to determine significant species. This test will only inform if there is a significant difference between two of the groups, and will not show which taxa change over time. For example, it might be the case group 3 happens to be different from group 7, but groups 1 and 11 are comparable. If the authors are trying to determine what species change over time there are established tools for this, such as MaAsLin2 or the random forest approach that forms for the basis for the microbiota by age analysis (doi: 10.1038/nature13421).

It is also not clear how samples from the same subject are distributed over the time intervals. Presumably the earlier time intervals contain mostly subjects from the “youngest age at enrolment” and the later time intervals containing “oldest age at enrolment”? Thus, how the authors accounted for this important bias – that the repeated measures are restricted to very specific time frames?

The title and abstract are focused on the “distinct non-industrial Prevotella-based trophic network”, yet this analysis represents only a minor portion of the results and is not presented until very late in the results. This relates to my previous comment of a need to reword the results and not dilute the main messages.

Regarding the trophic network analysis, the authors use phrases like “The most distinct and visually striking cluster”. It is not clear what is significant or biologically meaningful. I also found it difficult to follow the analysis in Figure 6 and how this relates to that in Figure 5. I suggest combining into a single cohesive or moving elements (e.g., Figure 6) to the supplement.

The WAZ, HAZ, and WHZ analysis does not add anything to the manuscript and could be removed to help focus the results, or at least condensed.

I suggest the authors consider including publicly available data from industrialised populations as a comparison. Given the paper is highly descriptive, this would significantly improve the impact of the work and highlight how different the development of the gut microbiome is in non-industrialised infants.

At 5 pages, the discussion is also on the longer side and would benefit from condensing and focusing on the main messages.

Minor comments

I cannot see a word count, but the manuscript may exceed the 3,500 suggest word limit for Nature Microbiology.

WAZ, HAZ, and WHZ need to be defined in the abstract

The "study setting" and "nutritional and diet" sections are helpful but could be provided in the supplementary material.

Did antibiotics have any significant impact on alpha diversity (e.g., richness or Shannon diversity)?

Check English vs American spelling – e.g., duel use of industrialised and industrialized

I am not sure what the genus and family level plots (Figure 1b and 1c) add beyond what is observed in the species plot (Figure 1a).

Figure 1 legend states "All Bacteroides species and all Bifidobacterium species were combined" but I am not sure what this means exactly or why it was done

I am not sure what is gained by doing numerous alpha-diversity metrics in Figure 2.

Lines 377 – 384 reference Supplementary figure 5, but I am not sure if this is the figure the authors are referring to.

The authors could explain upfront what is a "trophic network"

Scientific language could be improved. For example, "the Faecalibacterium & co. cluster does seem to benefit". I don't follow what "seem" means.

Author Rebuttal to Initial comments

Reviewer Comments:

Reviewer #1 (Remarks to the Author):

The gut microbiome of 7-37 month old children from The Gambia shows the development of a distinct non-industrial Prevotella-based trophic network
De Goffau et al

This manuscript presents data from a large substudy of an iron supplementation trial in The Gambia, which characterise the development of the microbiome in young children. The study provides normative profiles of maturation of the gut microbiota in rural West Africa, and as such will be very valuable to many researchers over the coming years. The main conclusion is that age is the dominant influence on microbiota composition, with no evidence of an effect of nutritional

status (though very malnourished children were excluded, so the power of this statement is somewhat dimnished). The microbial communities present were divided into four groups: Prevotella copri, the Prevotella stercorea group, the Faecalibacterium prausnitzii group, and unclassified/other. The authors identify the Prevotella stercorea group as a group rarely found in microbiomes from industrialised countries and conclude that this group is the archetype "good" microbiome cluster present in rural African
children. This is an important study, well-powered and robustly analysed, which puts forward a very interesting way of looking at geographical and diet-related microbiome variation.

Major comments
1 The title is a bit clumsy; "non-industrial" does not make grammatical sense unless referring to non-industrial society or non-industrial diet. Please re-phrase.

We have now used a term mentioned by reviewer 3:

"non-industrial" was changed to "rural pre-industrial"

2 In the Introduction the authors introduce the "enterotype" hypothesis, but then ignore this intriguing question and leave the reader somewhat dissatisfied that they do not in the end come off the fence and decide whether their data do or do not support this hypothesis. In my view their data could be used to refine the enterotype hypothesis into a more sophisticated classification. Do they agree?

We agree with the reviewer's comment specifically indeed that this data allows for a refinement of the enterotype hypothesis. Enterotypes are in a way a simplified representation of specific bacterial trophic networks. In reality it is btw much more of a gradient (with specific stable configurations along the way). This study in particular sheds more detail on (the build-up of) what is commonly seen as the *Prevotella* enterotype, namely that *Prevotella copri* should be seen as a somewhat distinct separate entity, capable of rising to dominance rapidly whilst not being very dependent on other species, and that various other gut *Prevotella* species steadily over time build up a complex trophic network. This study however does not increase our understanding about the *Bacteroides* or *Ruminococcaceae* enterotypes much (a European cohort would be more suitable) except that *P. copri* and *Faecalibacterium* appear to have a positive interaction. The negative interaction of *Bacteroides* with *Prevotella* was already well established. We've expanded upon these concepts in the discussion.

3 I was left wondering what criteria were used to define "trophic network clusters". In lines 410 ff there is a claim that the Bifidobacterium group is not a real "trophic cluster" but no evidence is provided to refute its definition as such. This term needs to be defined and its application justified when used to decide which are "real" trophic communities.

This part of the manuscript (clusters) needed quite a bit of additional work but has been improved quite a bit by directly linking up the heatmap with the PCA analyses as was suggested by one of the other reviewers.

In regards to the *Bifidobacterium* cluster, many of the positive associations of *Bifidobacterium* with other species are likely indirect as they are a result of these species simply steadily together disappearing/decreasing over time as the gut microbiomes composition matures. This is for example in particular true for several opportunistic pathogens and *Bifidobacteria*. Additionally, there are several lactate producers whom likely compete for the same breastmilk derived oligosaccharides. However, there is certainly also a real trophic network cluster present of which *Bifidobacterium* and *Megasphaera elsdenii* are a nice example. *Bifidobacterium* produces lactate and *Megasphaera elsdenii* converts this into butyrate (ref: https://pubmed.ncbi.nlm.nih.gov/29174148/ and https://journals.asm.org/doi/full/10.1128/AEM.02327-18). However, to achieve a better understanding of the "*Bifidobacterium*-trophic-network" within a rural pre-industrial environment one would need a large cohort of samples focussed on the age of 0-1 years old.

4 Pathogens were not specifically identified as the analysis used only 16S sequencing. Is it fair to comment on "potential" pathogens without data?

This is indeed a fair point from the reviewer.

We have now changed pathogens to "opportunistic pathogens" where appropriate.

We for example now also mention *E. coli* in regards to its association with the wet season. Typically, during the dry season diarrhoea is associated with the rotavirus whilst diarrhoea becomes more associated with bacterial opportunistic pathogens, like *E. coli*, during the rainy season (https://journals.plos.org/plosntds/article?id=10.1371/journal.pntd.0007211). This is now briefly mentioned in the discussion.

We have added to following paragraph to the discussion section

An additional interesting detail is the abundance of E. coli. Not only was E. coli found to be most abundant in young children and to decline in time but it was also associated with the rainy season. From literature it is known that diarrhoea is mainly associated with rotavirus infections in the dry season but with opportunistic pathogens during the rainy season.

"The higher abundance of Bifidobacterium in European children may also be a result of earlier and longer formula-feeding in those children."

5 Figures 5 and 6 are central to the argument of this paper. However, Figure 6e does not directly show the inverse correlation between the P. stercorea group and Bacteroides which is prominent in the Discussion (lines 533 ff). This should be shown directly rather than just as the subtraction for PC2.

The *Prevotella stercorea* trophic network more or less encompasses 2 clusters of bacteria (see heatmap) of which one cluster (the top one) has many positive correlations with the species associated with the *F. prausnitzii* cluster, encompasses many butyrate producers (which are quite promiscuous in regards to their bacterial partners which provide them with acetate/lactate and other things) and which are strongly negatively correlated with the abundance of *Bifidobacterium*. The core of the *Prevotella stercorea* trophic network however consists of (see heatmap):

Prevotella_stercorea_7.05	Succinivibrio_dextrinosolvens_2.42
Paraprevotella_xylaniphila_2.02	Sutterella_sp._0.82
Prevotella_ruminicola_0.62	Holdemanella_biformis_0.42
Catenibacterium_mitsuokai_0.29	Phascolarctobacterium_succinatutens_0.28
Pasteurella_multocida_0.25	And several smaller players not mentioned.

This core of the *Prevotella stercorea* network (9 species summed up) is strongly negatively correlated with *Bacteroides*, Spearman rho coefficient of -0.363, which is as strongly negatively correlated as *Prevotella copri* is with *Bacteroides* (Spearman rho coefficient of -0.358). We've added additional details to the heatmap and clarifications in the results section to better make the argument that both *P. copri* and the core of the *Prevotella stercorea* trophic network are strongly negatively correlated with *Bacteroides*.

Minor comments
6 Abstract: percentages shown are % of bacterial sequences, or % of children carrying them? Please clarify.
This is now fixed. We added *"across all bacterial sequences in all 1389 samples"* in line 57/58.

7 Presumably length was measured in children under 2.

Yes, that is correct. The height of all children in cm and mean (SD) is shown in Table 1.

8 How was antibiotic use ascertained? Online methods section 4.8 suggests (but does not state explicitly) that this was just from clinic records. How can you be sure that over-the-counter antibiotics did not confound the analysis?

Prof Andrew Prentice from The Nutrition Theme at MRC Unit The Gambia at LSHTM provided following answer to your question:

“Although antibiotics are commonly available from shops in many low-income settings this is not the case for this area of rural Gambia and we have never come across cases of local purchase.”

We have also reanalysed the entire study by taking out all 16 samples from 15 children who have officially received antibiotics to make the study dataset even clearer.

9 Spelling errors: funiformis (line 189); PERMANOVA (several instances read PERMANOA)

Thanks for pointing them out. The funiformis and the two PERMANOA spelling errors were corrected.

10 P=0.0099 should be shortened to P=0.01

P value of 0.0099 was corrected to P value of 0.01

11 Table 2 has no legend; the different measures (e.g., Chao1) should be explained. Some explanation/justification of Fisher’s is included in Methods, but it would be helpful to explain the measures used in the main text so that their significance is apparent to the reader.

The analysis shown in Table 2 has been now removed from the manuscript since we now also include individual alpha diversity analysis for the different timepoints "Day1, Day 15, and Day85", now shown in **Sup Fig 4** and explained in "2) Result – Alpha diversity analysis"

We have added a summary explaining the Fisher's alpha in the Alpha diversity analysis section" in the mean text.

12 Figure 3 legend has (a) and (b) but only one panel is shown.

The whole set of Figures has been changed, corrected, and updated.

13 Figure 5 should go before Supp Fig 4

The whole set of Figures has been changed, corrected, and updated.

14 What were the criteria for inclusion in the boxes in Figure 5? Why were *Megamonas* or *Sutterella* not included in the Bifido box (variously described as black or grey)?
This was an oversight from our side. We have generated new heatmap network figures with a slightly smaller dataset from which 16 samples from 15 patients were excluded (Antibiotic samples).

15 Online methods (Line 290) refer to the heatmap in Figure 4a; this should be 5a.

The whole set of Figures has been changed, corrected, and updated.

Reviewer #2 (Remarks to the Author):

Overall, I found this study to be of extreme interest and well carried out. However, there are a few issues that should be addressed:

1. Using Spearman or Pearson correlations to build trophic networks based on 16S abundances is problematic, since these methods result in many falsely positive erroneous network links (see Friedman & Alm 2012; Weiss et al. 2016). Filtering out lowly abundant sparse taxa, as the authors do, is good, but the analysis is still problematic. Considering this is time series data, Local Similarity Analysis might be more appropriate. (See Figure 7 of Weiss et al. 2016). Other programs, such as FastSpar (fast implication of SparCC) would be admissible. Further, determining groups of

correlated taxa by drawing boxes by observation is fine, but a more statistically robust approach would be to estimate the number of clusters using an algorithm such as the gap statistic.

We completely agree with the reviewer that a robust test should be employed to confirm whether optically identified clusters are statistically sound. For this we have now used gap statistic using the R function “clusGap” from the package “cluster”.

Supplementary Figure 13 shows the outcome from the gap statistical analysis.

We also agree with the reviewer that other methods for drawing the network heatmap should be tested. In this rebuttal we included a very comprehensive comparison network analysis between ELSA, SparCC, and Spearman correlation and came to the conclusion that Spearman correlation provided more meaningful/correct/logical network then the other two methods. Please, find detailed explanations below:

We have extensively investigated different approaches for network generation including ELSA (extended local similarity analysis) and FastSpar. **Weiss at et al. 2016 also investigated different methods for network generation including ESLA and FastSpar with the conclusion that “Current tools have significant limitations that must be accounted for when performing correlation analysis”**.

We have been in contact with the developer of ELSA to discuss the use of ELSA for our dataset. The ELSA developer was not sure whether ELSA is the appropriate tool for our dataset since ELSA was specifically developed for one sample datasets with many timepoints and several repeats. Our dataset has many samples, only three timepoints and no repeats.

We have nevertheless extensively tried to generate meaningful network data using ELSA but came to the conclusion that the ELSA data are indeed not useable for generating meaningful network clusters. For, example, ELSA would generate a very strong negative correlation between *P. copri* and *F. prausnitzii*, which is wrong as we show in several of our figures that this correlation is positive. We also concluded that all ELSA values appear rather extreme in our eyes (irrespective of parameters used) leading us to conclude that it is not suitable for making heatmaps with our dataset.

We also extensively validated the usefulness of SparCC. Unfortunately, however, SparCC also generated non logical correlation values. E.g. In regards to SparCC, this appears to generate positive correlations between *Prevotella copri* and *Bacteroides* (day 15 and day 85).

This is wrong and thus SparCC also does not seem fit for purpose. *P. copri* and *Bacteroides* are negatively correlated (spearman rho correlation coefficient of -0.36) and there is a large body of literature out there that would also argue that these 2 should be correlated negatively. The plotting of *P. copri* vs *F. prausnitzii* however generated a nice positive correlation. What we think is happening here is that SparCC cannot handle that *P. copri* is positively correlated with *F. prausnitzii* and negatively with *Bacteroides* whilst *F. prausnitzii* is also positively correlated with *Bacteroides*.

In Summary:

We are therefore convinced, that Spearman correlation, whilst probably imperfect, is still the best approach for our dataset which is also supported by another statement made by Weiss et al (2016) "All tools are sensitive to several distribution shapes, except for LSA, MIC, Spearman and SparCC" and "Rank-based measures such as MIC and Spearman, as well as Bray-Curtis, are less affected by compositional data"

Weiss et al (2016), also mentioned that "SparCC is particularly designed to deal with compositional data, as it is based on Aitchison's log-ratio analysis". However, our filtered dataset (less compositional*) performed extremely badly with SparCC and all the logical network associations identified by Spearman correlation diminished with SparrCC.

In addition, for our network generation, we have removed low abundant sparse taxa by only including the top 50 taxa with a minimum abundance of 0.2%. This is in concordance furthermore with Weiss et al. 2021, who reported an extensive comparison between the different methods for network generation recommended "filtering out extremely rare OTUs prior to network construction. Tool performance degraded significantly for OTUs containing > 50% of zeroes". In our dataset using the top 50 most abundant taxa, 38 taxa had less than 50% zeroes.

We hope that we have convinced the reviewer, after thoroughly testing of ELSA and SparCC, that the Spearman correlation approach appears to perform the most adequate as it provided the best logical network data.

2. Authors demonstrate that b-diversity between iron supplementation and placebo was not different. What about a-diversity? Similar question for the abx treated children. Since these samples are then all lumped together for subsequent analysis, which includes looking at a-diversity, it is important to establish how these factors (iron supplementation and abx treatment) affected microbiome a-diversity.
To answer the question, whether the placebo group and iron-treated group can be grouped

together for Alpha diversity analysis, we have now compared our four selected alpha diversity indexes between the iron-treated group and placebo group as recommend by the reviewer.

The alpha diversity indexes for the placebo and treatment group are shown in a Box and Whiskers plot with Whiskers showing the Min to Max value **in Sub Fig 2.** Figures and statistical calculation were done in Prism 9 for macOS. In summary, the four alpha diversity indexes were not significantly different between the iron-treated group and the placebo which justified analysing both groups together. Also, the figure outline was changed from strip column chart to Scatter dot plot with line at Mean with SD for the 11 age group comparisons.

Regarding antibiotics, we followed reviewer 3's recommendation and have removed 16 samples from 15 patients who received antibiotics leaving 1389 samples from 633 patients for detailed analysis. Thus, the first result section "Sampling framework and sample characterisation" was changed accordingly. The removal of abx made the dataset even cleaner and thus no analysis between iron supplementation and abx treatment was conducted. The antibiotic free dataset was completely re-analysed and "Table 1. Child cohort available for microbiome analysis" was changed accordingly"

3. I didn't see how authors dealt with longitudinal samples. Are they correcting for resampling of the same individuals in any way, specifically in their PERMANOVA and Spearman tests (for PCo correlations)?

We re-analysed the antibiotic free samples datasets with PERMANOVA and PCoA by further separating the three time points (7-12 mths "young", 1 to 2 years "middle", and plus 2 years "old") into day1, day15, and day 85 sampling timepoint.

The individual Beta diversity analyses (PCoA plot) for the combined timepoint (TP) samples and separate TP samples for iron-treated samples and placebo are presented in **Supp Fig 1.**

The individual Beta diversity analyses (PCoA plot and PERMANOVA and ANOSIM) for the combined TP samples and separate TP samples for the three age groups are presented in **Supp Fig 5.**

The individual Beta diversity analyses (PCoA plot and PERMANOVA and ANOSIM) for the combined TP samples and separate TP samples for the 11 3-months age groups are presented in **Supp Fig 6.**

The individual Beta diversity analyses (PCoA plot) for the combined TP samples and separate TP samples for gender and geographic are presented in **Supp Fig 7.**

The individual Beta diversity analyses (PCoA plot, PERMANOVA and ANOSIM) for the combined TP samples and separate TP samples for wet and try season are presented in **Supp Fig 8.**

In summary, all individual TP beta diversity analysis provided the same results as for the combined TP analysis, except for the DAY 85 samples in the wet and dry season analysis which seemed to be influenced by age.

4. Is the Simpson's index shown in Fig 2b significant between each pairwise comparison? Not clear from text which comparisons are significant.

The alpha diversity index is now shown in Figure 1. The alpha diversity section was completely rewritten and the alpha diversity section was also placed forward in the Result section.

To answer the reviewer's question:

We now included **Supplementary Table 1** which shows the Alpha diversity multiple group comparison using the Kruskal-Wallis Dun's test.

5. Similarly, in other diversity tests demonstrating significance, is this only between first and last time point groups? Between each group? Not clear from text.
Please see answer from above (Question 4)

6. These statements seem to contradict one another:
"P. copri and the P. stercorea network are negatively correlated with Bacteroides whilst the F. prausnitzii network is positively correlated with Bacteroides."

"PC4 in Figure 6.f indicates that P. copri and the F. prausnitzii network are positively correlated with one another"
Given the negative correlation between Prevotella and Bacteroides, how can both be positively correlated with F. prausnitzii?

This apparent contradiction is likely also the reason why certain clustering methods appear to have trouble with this dataset (ELSA and SparrCC). We're dealing here with a triangular situation where, in particular *F. prausnitzii*, is quite promiscuous in regards to which bacterial partners it can benefit from whilst *P. copri* and *Bacteroides* are known to be strongly mutually exclusive. *P. copri* and *Bacteroides* are STRONGLY negatively correlated with one another (rho: -0.36), *P. copri* and *F. prausnitzii* are when taking all samples together somewhat positively correlated with one another (rho: 0.10) and *F. prausnitzii* and *Bacteroides* are somewhat positively correlated with one another (rho: 0.13). Visually however (using an x-y scatterplot), *P. copri* and *F. prausnitzii* seem more strongly correlated with one another in samples with at least 10% *P. copri* (**Figure 6b**). If one

excludes samples with more than 5% *Bacteroides* the correlation coefficient between *P. copri* and *F. prausnitzii* rises from 0.10 to 0.15 and if one for example excludes all samples with more than 20% *P. copri* the spearman rho coefficient between *F. prausnitzii* and *Bacteroides* rises from 0.13 to 0.27. In short, gut microbiome interactions/compositions are not binary but rather complex.

Minor issues:

- Line 153: Given that age is an important feature in the PCoA, how was uneven group sizes accounted for the age bins?

Here the reviewer refers to the PCoA between small numbers of patients who have received antibiotics - 15/633 patients. Since we have now excluded those 15 patients, we

have subsequently removed the PCoA analysis the reviewer has referred. However, as other PCoA results remain in the paper, we have included a response to the question below.

As PCoA is a map-based visualisation of the maximised distance between items, the plotted points in each overall PCoA are based on all observed data, which are then coloured by age group. Because of this, the positioning of the points on the plot are not influenced by how many individuals are in each age group. For this reason, we do not anticipate that the differences in sample size would influence the PCoA plots, despite the discrepancy between the sample size of the youngest and oldest age groups (190 and 616 respectively).

- It would be helpful if the authors described their definition of species. Are ASVs/OTUs agglomerated at the species level? If so, it could present a major issue, since all non-assigned taxa will be assigned to NA or thrown out (depending on parameters chosen). Either way, they will contribute in a statistically inadmissible way or not at all to multivariate analyses. Also, given that a population like that in rural Gambia is relatively unexplored, by binning NAs or removing them, diversity could be missed.

We have not generated NA assigned species. The description for the species identification is described in the online supplement section "4.2 Oligotyping and species/taxa identification.

We have added additional information at the end of this section to make it clearer.

In cases where a species could not be classified, we reported the genus name and in few cases the family name. For 2 well known important groups of commensals with (largely) the same function (niche), we however combined all species within the same genus. E.g., All *Bifidobacterium* species were combined to *Bifidobacterium* and all *Bacteroides* species into *Bacteroides*. This was done to get

a stronger signal for these important taxa in the analysis. Analysis on the species-level for these genera would mainly just have generated more noise (including many zero's) and a biologically less interpretable signal.

- The text for Fig 3 states that a Kruskal Wallis test on ranks (line 294) was used but the figure says ANOVA in the panels.

Thank you for pointing this out.

This was a miss-labelling from the program. The heading in the Fig 4 (former Fig 3) was changed.

- I don't see an A and B in figure 3. Is the legend correct?

The whole set of Figures has been changed, corrected, and updated.

- Line 545 referring to an "African" microbiome is very general, which cohort and reference are being referred to here?
We have added two references referencing the Burkina Faso and the Malawi study and have added the two country names into the text at line 527.

Reviewer #3 (Remarks to the Author):

De Goffau and Jallow et al., Investigate the microbiome structures of 616 rural infants from Gambia. The topic of the developing infant microbiome has been an area of intense research but mainly focused on industrialized populations. Pre-industrial populations, and how the infant gut microbiota develops is therefore of interest. The authors confirm that in pre-industrial populations Prevotella are likely a key component of the microbial communities in the gut.

Major points
In the introduction the concept of gut enterotypes is introduced and the authors note that the concept is debated in the field. Adding to this debate, it has been suggested that the Prevotella/bacteroides divide is an artefact of relative abundancies: DOI: 10.1038/nature24460. For completeness this could also be added to the introduction. In addition, such findings will have implications on the correlative associations observed (figure 5) by the authors and would be worth discussing in this context also.

We appreciate the concept of a combination between relative bacterial abundance analysis and the use of flow cytometric enumeration of microbial cells as discussed in the Nature paper mentioned by the reviewer. To undertake bacterial cell count in faecal material for such a large number of samples (in the 1000s) shortly after stool collection is however not feasible in many non-industrial countries including many countries in Africa. It would even difficult in many industrial countries. In addition, most likely, repeated stool sample from the same individual may contain different number of bacterial cell counts. This would make the highly informative flow cytometric enumeration of microbial cells even more challenging if for example the average of several stool collections from one individual must be enumerated.

But it is indeed a very interesting and important concept and it highlights that stool microbiome research still has several facets of uncertainty. We have included the following paragraph into the introduction section to highlight this paper (Line 109 to 112)

Others not only retain these 3 enterotypes but also split up the Bacteroides enterotype based (amongst others) on the actual bacterial load per gram of faecal matter[17] resulting in 4 enterotypes yet at the same time call into question the Prevotella/Bacteroides divide as it (partially) might be an artefact of the use of relative abundances further complicating the concept of Enterotypes.

We have also included a paragraph into the discussion section (line 515 to 522) to highlight this complexity:

Our correlation heatmap was based on relative bacterial abundance data. The use of relative bacterial 16S sequence data is currently the most common normalization in field of microbiome research. However, evidence is coming forward that that correction of the microbial load by enumeration of microbial cells in faecal matter through flow cytometric counting would provide a more realistic picture of bacterial abundances [17]. However, to undertake bacterial cell counting in faecal material for large numbers of samples (in the 1000s) and in particular in non-industrial countries such as many countries in Africa shortly after stool collection is simply not feasible.

Lines: 135-138. In the description of the cohort, while you mention malnutrition and as exclusion criteria. Regarding health of participants there is no mention of intestinal parasites. With rural pre-industrial populations, it is likely that infection is common and thus intestinal inflammation, which could be relevant when looking at the microbiota. Was any information regarding intestinal parasitism and inflammatory status collected?

Prof Andrew Prentice from The Nutrition Theme at MRC Unit The Gambia at LSHTM provided following answer to your question.

"For this large Gate's founded clinical trial, the Kato-Katz method was used to collect information about Helminth parasite egg count and faecal calprotectin.

We received information from the Gambia that very few children had helminths because there is a national program of anti-helmitic metronidazole every 6 months in the Gambia.

Children were not given anti-helminths at the start of the study. Regarding gut inflammation, the Gambia team did measure faecal calprotectin. This was a secondary outcome of the trial and will be reported in the main trial outcome paper. We agree that microbial association analysis with calprotectin could be another interesting outcome measure of this study, but we also believe that it would expand the manuscript beyond its current intended scope."

Additionally, and sorry if I missed this, but I do not see the metadata associated with each participant in the study as supplementary material. Only a summary of the cohort in Table 1. Such information would be useful for other researchers in meta-analysis investigations.

Thanks for pointing this out. This was an oversight from our side.

We have now added **Supplementary Table 4** showing the metadata and count data for all 1389 samples. A statement about **Supplementary Table 4** is present at the end of the manuscript, before the references.

Development of the early life microbiome has been the focus of research attention for a number of years. The opportunity from a pre-industrial population is to find parallels and contrast with that of industrialized populations. There is a limited comparison to other cohorts, the manuscript would be strengthened by placing the Gambia cohort in the context of existing literature, both from industrialized and pre-industrialized populations. For instance, pre-industrialized doi.org/10.1038/s41598-019-49274-y; doi.org/10.1111/ppe.12623; DOI: 10.1016/j.celrep.2018.05.018

Thank for providing the link to these important African study from Malawi and Nigeria.

We have discussed the major findings from these papers in context with our Gambia Data together with other studies already referenced in the discussion section (Yatsunenko 2012, Nature, De Filippo 2010 PNAS, and Schnorr 2014 Nat. Com). We have now summarized other selected studies

together with the above-mentioned studies in regards with *Prevotella* information in **Supplementary Table 4**. All the studies are now discussed in the discussion section.

Line 556-569: The authors mention the *P. copri* abundance in their cohort emphasizes its importance in fermenting complex carbohydrate. While it is true *P. copri* utilizes carbohydrates, the benefit of *P. copri,* particularly in association with glucose metabolism, has received contradictory reports as to whether it is beneficial or not.

Also, *Bacteroides* which are believed to be excluded by Prevotella and dominate industrialized microbiomes are also known fermenters and have a large number of carbohydrate utilization pathways. This highlights that the intestinal dynamics, industrialization and shifts in diet are complex and could be mentioned in the discussion.
We have added additional information of some interesting importance of *P. copri* and *Bacteroides* into the discussion section in line 548 to 562.

In recent years P. copri has been the subject of contradictory reports as to whether it is beneficial or not; particularly P. copri was implicated as a risk for rheumatoid arthritis [38,39], and Prevotella intestinalis was reported in a mouse model to alter the composition and function of the ecosystem resulting in a reduction of short-chain fatty acids, specifically acetate [40].

However, the largest (to date) cross-continent P. copri metagenomic analysis using over 6500 metagenomes by the Huttenhower group reported notable differences in carbohydrate metabolism driven by dietary modifications resulted in a reduced prevalence in industrialized populations [41]. The same group also reported four distinctive globally distributed P. copri clades of country specific sub-types and in their meta-analysis they found no strong evidence that any of the four clades were associated with disease. Finally, their gene function analysis revealed some interesting clade specific metabolic pathways [41]. Our high Prevotella-to -Bacteroides ratio (~1:10) also highlights an interesting intestinal dynamic which changes through industrialization and shifts in diet [10]. A high Prevotella-to-Bacteroides (P/B) ratio pre-treatment has been associated with more effective loss of body weight when utilizing high fiber diets [42].

A finding of this study is the abundance of Prevotella and particularly P. copri in the infant microbiome. The substantial sub-species diversity of P. copri over the past few years has become increasingly evident. The resolving power of 16S is limited but did you look for and identify sub species types for the Prevotella species?

Using a different pipeline it might indeed be possible to achieve a resolution for some species at a strain-like level (using amplicon sequencing variants, DaDa2) but we specifically chose to limit ourselves (using Oligotyping) to the species level (which many think still cannot even be done with 16S sequencing), and in the case of *Bifidobacterium* and *Bacteroides* even to the genus level, in order to limit the amount noise in the data. Doing so might indeed miss some particular details of relevance but in general increases the biological interpretability of the data as many of the strains of the same species have (largely) the same functions and the same associations with other species with whom they form a trophic network (or an antagonistic interaction). In relation to *P. copri*, it does not appear to form dichotomous relationships/patterns (as far as we've observed) with other bacteria that might be indicative of different *P. copri* strains with very different properties and interactions.

The question of the reviewer is nonetheless interesting but would likely be better answerable using longer-read technology, such as PacBio sequencing using the full-length 16S genome in combination with the 23S genome in cases where groups don't have the financial and bioinformatics man-power to analyse large number of samples by shot-gun metagenomic sequencing.

Probably a simple oversight, but certain statements in the manuscript should be backed-up by references. This should be checked throughout the manuscript. For example:

Line 410: "These positive associations do not seem to represent a trophic network except possibly for the association of Bifidobacterium (which produces lactate)" reference(s) needed
Line 411 "Megasphaera elsdenii (which metabolizes lactate and produces butyrate)." Reference(s) needed

Reference should all be included and updated now.

Line 542: "Similar to infant microbiotas in industrialized countries, the Bifidobacterium genus is the

most important and dominant group early in infancy" Reference(s) needed.
Line 524 to 525 now references Bifidobacterium.

Similar to infant microbiotas in industrialized countries, the Bifidobacterium genus is the most important and dominant group early in infancy [30,31] (reviewed in [32]).

Minor points
Lines 151: At the opening of the Results section effort has been taken to demonstrate that antibiotic usage has little to no consequences on the samples from those participants. Based on the relatively small number of participants receiving antibiotics (2.4%), I wonder if it is not simpler and cleaner to remove those subjects?

We agree with the reviewer and have removed 16 samples from 15 patients who received antibiotics leaving 1389 samples from 633 patients for detailed analysis. Thus, the whole study was re-analysed without the antibiotic samples.

Alpha diversity: Figure 2 is dedicated to the changes in alpha diversity. Other than presenting the changes in the results these analyses, and significance thereof are not commented on in the discussion?
We have rewritten the alpha diversity analysis result section and discussed it in the discussion section, and also added a more detailed alpha diversity analysis as supplemental material.

Line 215: Reference to the cohort as patients. Wouldn't participants be a more fitting term?

We fully agree, we have changed patients to participants. The children are indeed not patients but were participants in the trial.

Figure 1: Check Plot axes for formatting errors

Most of the figures were redone and updated

Reviewer #4 (Remarks to the Author):

The manuscript by De Goffau & Jallow and colleagues present faecal microbiome data collected from month 7-37 of life from subjects in the The Gambia. The original study aimed to investigate whether children receiving iron supplementation differed from the placebo group. However, as no differences between groups were observed, the authors instead conducted a more descriptive analysis of gut microbiome development over time in African children. Given the unique cohort, this is still a worthy pursuit and I was pleased to see authors from The Gambia included on the publication. My major concerns with the analysis relate to the way the different statistical tests have been performed, which have an inconsistent angle of age/time, and in many cases an apparent failure to account to repeated measures. I also feel the manuscript lacks a clear narrative and would greatly benefit from a critical rewrite and focusing of the main messages. I expand on these and other points below which I hope the authors will find helpful.

At 27 pages, the results section is very long and at times lacks a clear narrative. This is further complicated as throughout the results the authors split the data into various different time groupings, such as “age at enrolment”, “timepoint”, and “age in months”. It can be difficult to follow why samples have been split as they have and what the different way of presenting the data adds. The manuscript would improve drastically with a critical rewrite of the results to make it more focused and with a constant narrative throughout.

We have completely re-written the result section and provided bullet point heading at the start of the result sections to provide a hopefully more understandable flow of the results

The Result section summarizes the following findings:

1) *Justification to combine treatment and placebo groups*
2) *Alpha diversity analysis*
3) *Beta diversity analysis*
4) *Multivariable statistical analysis to identify taxa associated with age, season, and treatment*
5) *Taxonomic differences between the young, middle, and old age groups*
6) *Taxonomic differences between the 11 3-months age groups*
7) *Result - Correlation analysis identifies distinct clusters representing bacterial trophic networks*

The introduction reads more like a discussion in parts and could be improved to give a broader overview that is relevant to the current study. Currently it focuses primarily on 1) one study by Yatsunenko and 2) the concept of enterotypes, neither of which set the scene for why the current study is important.

The bits about enterotypes have been adjusted throughout the manuscript to better answer several questions of reviewers. We agree that this study does not really inform the reader much about the different enterotypes but it does add extensive insight into the formation of what is commonly regarded as the Prevotella-enterotype. Specifically, we now mention the following in the discussion: Line 433 to 442

"This study does not aim to disentangle the discussion about enterotypes yet it does provide new insights in regards to what has thus far been regarded as the Prevotella enterotype. This study shows that the Prevotella genus should not be seen as a monolithic entity but that it consists out of at least 2 important and seemingly independent components. In these children, which can be said to develop a Prevotella-enterotype composition, Prevotella copri rises to dominance rapidly in the first 12 months and remains the most abundant species during the remainder of early childhood (~35% of all reads), irrespective of the abundance of other species. Other Prevotella species are however part of a network that increases much more gradually over time in abundance in what has been coined in this study as the Prevotella stercorea trophic network. "

How did the authors account for repeated measures in their analysis? For example, in Figure 1 box plots and the supporting PERMANOVA test, would more than 1 sample from a given subject appear within the same age group? If so, a PERMANOVA test, which assumes cross-sectional study design, would not be a valid approach. Similarly for analysis in Figures 3, 4, and throughout, how were multiple comparisons dealt with?
Reviewer 2 (Question 3) also asked the same questions. Please see our reply below:

We re-analysed the antibiotic free samples datasets with PERMANOVA and PCoA by further separating the three time points (7-12 mths "young", 1 to 2 years "middle", and plus 2 years "old") into day1, day15, and day 85 sampling timepoint.

The individual Beta diversity analyses (PCoA plot) for the combined timepoint (TP) samples and separate TP samples for iron-treated samples and placebo are presented in **Supp Fig 1.**

The individual Beta diversity analyses (PCoA plot and PERMANOVA and ANOSIM) for the combined TP samples and separate TP samples for the three age groups are presented in **Supp Fig 5.**

The individual Beta diversity analyses (PCoA plot and PERMANOVA and ANOSIM) for the combined TP samples and separate TP samples for the 11 3-months age groups are presented in **Supp Fig 6.**

The individual Beta diversity analyses (PCoA plot) for the combined TP samples and separate TP samples for gender and geographic are presented in **Supp Fig 7.**

The individual Beta diversity analyses (PCoA plot, PERMANOVA and ANOSIM) for the combined TP samples and separate TP samples for wet and try season are presented in **Supp Fig 8.**

In summary, all individual TP beta diversity analysis provided the same results as for the combined TP analysis, except for the DAY 85 samples in the wet and dry season analysis which seemed to be influenced by age.

Only P values adjusted for multiple comparisons should be presented. Mention of "significant" features before adjusting for multiple comparisons should be avoided.

For multiple group Beta-diversity analysis we now only show the Bonferroni P value.

For other analysis we now show only the corrected P values.

In the analysis looking at the different time points (e.g., lines 212 – 232) the authors include all subjects, regardless of age at enrolment. I am not sure this is the best approach, as one can expect a vastly different gut microbiome between the three age categories based on many factors, most notably diet (specifically whether they are breastfed or not). The amalgamation of the different age ranges might explain why so few species were found to differ between the different time points? Do the authors find a consistent result if the analysis is stratified by the previously used "age at enrolment" subgroups?

We have completely re-analysed the entire cohort with different statistical method to account for repeated measurements.

We have specifically used mixed-effect linear regression to examine the effect of timepoints, adjusting for 3 and 11 age categories and the other metadata.

We now also included the results from a "Multivariable statistical analysis" using the R package MaAsLin2 to identify taxa significantly associated with age. The MaAsLin2 analysis was recommended by one of the other reviewers.

The authors state "age was the only contributing factor which differentiated the gut microbiomes of samples in this cohort". However, it is not clear what this is based on and what other "factors" were tested. As this paper is largely descriptive, it would be beneficial to perform an analysis of all factors of interest and determine what explains the most variance / what is most significant (e.g., using

adonis or envfit). Given the well reported impact of factors such as subjectID, birth mode, and diet on gut microbiome development during infancy/childhood of industrialised infants, similar analysis from the current non-industrialised population would serve as a nice comparator.

In our study we analysed the following variables in the Beta diversity analysis:

1) AGE, and age split into three age groups and 11 3-months age groups
2) Gender
3) Geographic location
4) Participant groups (Treatment and Placebo)
5) Season (Wet and Dry)

These different groupings have now been analysed in detail by PCoA multivariate analysis and by two different Beta diversity indexes including PERMANOVA and ANOSIM.

Age was by far the most discriminating factor. Season had a minor effect. Others did not appear relevant.

Diet was very much homogeneous and we do not have the data for mode of delivery but CS is extremely low in this region so there would at most be a handful of cases which would not allow statistical analysis.

It is not clear why the 11 age groups were created and why the Kruskal-Wallis rank test was used to determine significant species. This test will only inform if there is a significant difference between two of the groups, and will not show which taxa change over time. For example, it might be the case group 3 happens to be different from group 7, but groups 1 and 11 are comparable. If the authors are trying to determine what species change over time there are established tools for this, such as MaAsLin2 or the random forest approach that forms for the basis for the microbiota by age analysis (doi: 10.1038/nature13421).

We have taken the reviewer's suggestion into account and have used Huttenhower's tool MaAsLin2 to determine what taxa change over time. A new bioinformatic section (4.6) has been added to the online supplement. The top taxa identified from MaAsLin2 analysis were then plotted in a stacked line chart to show development at 11 3-months age group intervals. For the combined time point analysis, we also used age at enrolment and used the "subject identifier" as random effect. The subject identifier denotes the individual sampling timepoints.

It is also not clear how samples from the same subject are distributed over the time intervals. Presumably the earlier time intervals contain mostly subjects from the "youngest age at enrolment" and the later time intervals containing "oldest age at enrolment"? Thus, how the authors accounted for this important bias – that the repeated measures are restricted to very specific time frames?

We have completely reanalysed the entire cohort by taking the different sampling timepoints (repeated measurements) into account using mixed-effect linear regression which is now explained in result section 5.

For the 3 age group comparisons we now plot the individual sampling timepoints of the top 10 taxa in Figure 2.

The title and abstract are focused on the "distinct non-industrial Prevotella-based trophic network", yet this analysis represents only a minor portion of the results and is not presented until very late in the results. This relates to my previous comment of a need to reword the results and not dilute the main messages.

The results section was completely redone, and we have changed the title of the manuscript to:

The gut microbiome of 7-37 month old children from The Gambia shows a Prevotella rich gut microbiome and the development of a rural pre-industrial Prevotella-based trophic network

Regarding the trophic network analysis, the authors use phrases like "The most distinct and visually striking cluster". It is not clear what is significant or biologically meaningful. I also found it difficult to follow the analysis in Figure 6 and how this relates to that in Figure 5. I suggest combining into a single cohesive or moving elements (e.g., Figure 6) to the supplement.

We thank the reviewer for the comment about combining these two figures making them into a more cohesive whole. We kept the figures separately though (combining them into 1 Figure would have been too large) but integrated their findings using the clusters from the heatmap directly in a 1:1 fashion (only using the species shown in the heatmap per cluster) to give much more accurate descriptions of the first three principal components. We furthermore better highlighted the negative associations found in the heatmap that are visualized by PC2 and PC3 (*Bacteroides* vs *Prevotella* and Trophic networks vs *Bifidobacterium*, respectively). The quality of both figures has improved tremendously as a result.

In addition, Reviewer 2 requested:

"A more statistically robust approach would be to estimate the number of clusters using an algorithm such as the gap statistic".

We now used gap statistic which confirms the different clusters seen in the heatmap dendrogram. Supp Fig 13

The WAZ, HAZ, and WHZ analysis does not add anything to the manuscript and could be removed to help focus the results, or at least condensed.

We have followed the reviewer's recommendation and have removed this section. This also helped us to bring the word count down.

I suggest the authors consider including publicly available data from industrialised populations as a comparison. Given the paper is highly descriptive, this would significantly improve the impact of the work and highlight how different the development of the gut microbiome is in non-industrialised infants.

We have now included additional studies throughout the manuscript from industrialised and non-industrialised countries to further highlight the differences and commonalities between these two different populations.

To do a more comprehensive comparison between our bacterial 16S dataset and publicly available bacterial 16S datasets in the form of a review would be beyond the already very comprehensive scope of this paper.

However, we have compared our study with some of other interesting published studies from Children with an emphasis/focus on *Prevotella* abundances. This information is now presented in Supplementary Table 3.

Our study was compared with a study from

1) Malawi children 18 months and 30 months
2) Arizona USA, with 40 children with neurotypical condition and ASD
3) Burkina Faso and Tuscany Italian study
4) HIV positive and negative children study from India
5) Australia with two children's cohorts, with or without behavioural problems
6) Leyte Island in the Philippines from two cities, one with high fast-food consumption and the other with a healthier diet.

These studies generally reported lower levels of Prevotella in more industrialized settings, a higher abundance of *Prevotella* with a healthier diet, More Prevotella associated with non-disease

conditions, such as in the case of ASD/non-ASD and behavioural problems and non-behavioural problems but interestingly an opposite effect was observed in regards to HIV status.

At 5 pages, the discussion is also on the longer side and would benefit from condensing and focusing on the main messages.
We have modified the discussion section but unfortunately it did not get shorter since the other reviewers asked for additional studies to be mentioned in the discussion section.

Minor comments

I cannot see a word count, but the manuscript may exceed the 3,500 suggest word limit for Nature Microbiology.
With all the additional information added to the manuscript as requested by all four reviewers the word count is now: 5800

WAZ, HAZ, and WHZ need to be defined in the abstract.

This has been now removed from the abstract, since the whole section about WAZ, HAZ, and WHZ has been removed.

The “study setting” and “nutritional and diet” sections are helpful but could be provided in the supplementary material.

This has been now moved to the Online Supplement Section 1

Did antibiotics have any significant impact on alpha diversity (e.g., richness or Shannon diversity)?
Antibiotic samples (N16) have been removed as suggested by one of the other reviewers and hence the entire dataset was completely re-analysed. We only had a very limited number of antibiotics treated children (9) and were thus not in the position to adequately address this relevant question.

Check English vs American spelling – e.g., duel use of industrialised and industrialized

We have now used English spelling throughout the manuscript

I am not sure what the genus and family level plots (Figure 1b and 1c) add beyond what is observed in the species plot (Figure 1a). Figure 1 legend states “All Bacteroides species and all

Bifidobacterium species were combined" but I am not sure what this means exactly or why it was done.

We have removed the genus and family level plots and only reported the species level where taxonomically possible with the exception of *Bacteroides* and *Bifidobacterium* which we kept at the genus level. This was done to get a stronger signal for these important taxa in the analysis. Analysis on the species-level for these genera would mainly just have generated more noise (including many zero's) and a biologically less interpretable signal. The heatmap and PCA analyses now furthermore better highlight the importance of these 2 genera.

I am not sure what is gained by doing numerous alpha-diversity metrics in Figure 2.

The Chao 1, the overserved Richness, and the Simpson's alpha diversity are most commonly reported alpha diversity indexes. These three indexes are very useful to compare our study with other studies. The Fisher's alpha has been included in this study for the reason outlined in the appropriate sections.

Lines 377 – 384 reference Supplementary figure 5, but I am not sure if this is the figure the authors are referring to.
The entire result section was re-written and the figure issue should have been fixed

The authors could explain upfront what is a "trophic network"
The trophic network is explained at the beginning of the 7th result section.

Scientific language could be improved. For example, "the Faecalibacterium & co. cluster does seem to benefit". I don't follow what "seem" means.

These has been now fixed throughout the manuscript

Decision Letter, first revision:

Dear Josef,

Thank you for submitting your revised manuscript "The gut microbiome of 7-37 month old children from The Gambia shows a rural pre-industrial Prevotella-based trophic network" (NMICROBIOL-21020383A). It has now been seen by the original referees and their comments are below. The

reviewers find that the paper has improved in revision, and therefore we'll be happy in principle to publish it in Nature Microbiology, pending minor revisions to satisfy the referees' final requests and to comply with our editorial and formatting guidelines.

If the current version of your manuscript is in a PDF format, please email us a copy of the file in an editable format (Microsoft Word or LaTex)-- we can not proceed with PDFs at this stage.

We are now performing detailed checks on your paper and will send you a checklist detailing our editorial and formatting requirements in about a week. Please do not upload the final materials and make any revisions until you receive this additional information from us.

Thank you again for your interest in Nature Microbiology Please do not hesitate to contact me if you have any questions.

Sincerely,

{redacted}

Reviewer #1 (Remarks to the Author):

The authors have carefully revised the manuscript in the light of the reviewers' comments and in doing so I think they have made considerable improvements. There are a few outstanding points:

Major
1 In response to my question about calling E. coli pathogens, the authors have re-designated them "opportunistic pathogens", but on what grounds? My point was that treating all E. coli as pathogens is not justified on the basis of 16S sequencing. For that we would need to know about lt, eae, ipaH, aat and quite a few other genes. I am as unconfortable, if not more so, about "opportunistic pathogens" as I was about "pathogens". My point was that some E. coli are demonstrably pathogenic, but not all. It would be less imprecise to describe it as "a species which includes many important diarrhoeagenic pathogens".

Minor
2 One row in Table 1 has n-640. How?
3 The 'bullet points' at the beginning of Results should be removed.
4 In line 186, this was not a case/control study but a controlled trial.
5 I could not find a citation in the text to Figure S4
6 The text is confusing about Figure 5 and Figure S12, only one of which has dotted lines in the dendrogram.

Reviewer #2 (Remarks to the Author):

Overall, I find the manuscript much improved and the shifted focus of the analysis and discussion is of more broad interest to the microbiome field. I have just a couple remaining minor points.

1. The below statement is confusing. What is meant by “our”? I assume it means the data presented in this manuscript. Best to reword to be clear. Also, it seems the ratio is reversed (ie should be 10:1 not 1:10)?

"Our high Prevotella-to-Bacteroides ratio (~1:10) also highlights an interesting intestinal dynamic which changes through industrialization and shifts in diet."

2. The final statement of the manuscript is problematic given the prior statements in the discussion. Are opportunistic pathogens more of an issue in industrial, low prevotella microbiome people? I think an argument could be made that the opposite is true, however, it would be difficult to disentangle the effects of abx use and sanitation practices in industrialized regions of the world on opportunistic pathogens vs loss of prevotella. It seems that an argument could be made (and is made in the prior statements in the discussion) that the prevotella networks could play a role in keeping diseases of affluence at bay. This final statement could be interpreted as saying that Bacteroides are opportunistic pathogens given that the manuscript demonstrates their anti-correlation, which I don’t think is what the authors are implying.

“The parallel loss of P. copri and the P. stercorea trophic network, whose development is described here, indicates the likely importance of the metabolic pathways and capabilities of Prevotella and species associated with them, both for maintaining a higher fermentative capacity (SCFA production), a healthier gut microbiome environment and for keeping opportunistic pathogens at bay.”

Reviewer #3 (Remarks to the Author):

De Goffau and Jallow et al., have improved the manuscript and taken into consideration of the reviewers comments. However, I would add a couple minor points. Firstly, in response to potential influence of intestinal parasites and gut inflammation, the authors respond to the comment but it would be good if this was noted in the methods when describing the cohort. Secondly, on lines 552-553, The Huttenhower group is credited with the P. copri meta analysis (https://doi.org/10.1016/j.chom.2019.08.018), while true that C. Huttenhower was involved in the study, the main lead on the project should be accredited to N. Segata.

Reviewer #4 (Remarks to the Author):

The authors have recognized that many of the statistics presented initially suffered from bias, such as not accounting for repeated measures. Having addressed these issues the manuscript is now much improved and could be published if the other reviewers are satisfied. I have only one point of clarification:

- Can the authors confirm that whenever they used statistics such as PERMANOVA, that does not account for repeated measures, they only include 1 sample per subject?

natureresearch

Decision Letter, final checks:

Dear Josef,

Thank you for your patience as we've prepared the guidelines for final submission of your Nature Microbiology manuscript, "The gut microbiome of 7-37 month old children from The Gambia shows a rural pre-industrial Prevotella-based trophic network" (NMICROBIOL-21020383A). Please carefully follow the step-by-step instructions provided in the attached file, and add a response in each row of the table to indicate the changes that you have made. Please also check and comment on any additional marked-up edits we have proposed within the text. Ensuring that each point is addressed will help to ensure that your revised manuscript can be swiftly handed over to our production team.

We would like to start working on your revised paper, with all of the requested files and forms, as soon as possible (no later than two weeks). Please get in contact with us if you anticipate delays.

When you upload your final materials, please include a point-by-point response to any remaining reviewer comments.

If you have not done so already, please alert us to any related manuscripts from your group that are under consideration or in press at other journals, or are being written up for submission to other journals (see: https://www.nature.com/nature-research/editorial-policies/plagiarism#policy-on-duplicate-publication for details).

In recognition of the time and expertise our reviewers provide to Nature Microbiology's editorial process, we would like to formally acknowledge their contribution to the external peer review of your manuscript entitled "The gut microbiome of 7-37 month old children from The Gambia shows a rural pre-industrial Prevotella-based trophic network". For those reviewers who give their assent, we will be publishing their names alongside the published article.

Nature Microbiology offers a Transparent Peer Review option for new original research manuscripts submitted after December 1st, 2019. As part of this initiative, we encourage our authors to support increased transparency into the peer review process by agreeing to have the reviewer comments, author rebuttal letters, and editorial decision letters published as a Supplementary item. When you submit your final files please clearly state in your cover letter whether or not you would like to participate in this initiative. Please note that failure to state your preference will result in delays in accepting your manuscript for publication.

Cover suggestions

As you prepare your final files we encourage you to consider whether you have any images or illustrations that may be appropriate for use on the cover of Nature Microbiology.

Covers should be both aesthetically appealing and scientifically relevant, and should be supplied at the best quality available. Due to the prominence of these images, we do not generally select images featuring faces, children, text, graphs, schematic drawings, or collages on our covers.

We accept TIFF, JPEG, PNG or PSD file formats (a layered PSD file would be ideal), and the image should be at least 300ppi resolution (preferably 600-1200 ppi), in CMYK colour mode.

If your image is selected, we may also use it on the journal website as a banner image, and may need to make artistic alterations to fit our journal style.

Please submit your suggestions, clearly labeled, along with your final files. We'll be in touch if more information is needed.

Nature Microbiology has now transitioned to a unified Rights Collection system which will allow our Author Services team to quickly and easily collect the rights and permissions required to publish your work. Approximately 10 days after your paper is formally accepted, you will receive an email in providing you with a link to complete the grant of rights. If your paper is eligible for Open Access, our Author Services team will also be in touch regarding any additional information that may be required to arrange payment for your article.

Please note that you will not receive your proofs until the publishing agreement has been received through our system.

Please note that <i>Nature Microbiology</i> is a Transformative Journal (TJ). Authors may publish their research with us through the traditional subscription access route or make their paper immediately open access through payment of an article-processing charge (APC). Authors will not be required to make a final decision about access to their article until it has been accepted. Find out more about Transformative Journals

<B>Authors may need to take specific actions to achieve compliance with funder and institutional open access mandates. For submissions from January 2021, if your research is supported by a funder that requires immediate open access (e.g. according to Plan S principles) then you should select the gold OA route, and we will direct you to the compliant route where possible. For authors selecting the subscription publication route our standard licensing terms will need to be accepted, including our self-archiving policies. Those standard licensing terms will supersede any other terms that the author or any third party may assert apply to any version of the manuscript.

For information regarding our different publishing models please see our Transformative Journals page. If you have any questions about costs, Open Access requirements, or our legal forms, please contact ASJournals@springernature.com.

Please use the following link for uploading these materials:
{redacted}

If you have any further questions, please feel free to contact me.

{redacted}

Reviewer #1:
Remarks to the Author:
The authors have carefully revised the manuscript in the light of the reviewers' comments and in doing so I think they have made considerable improvements. There are a few outstanding points:

Major
1 In response to my question about calling E. coli pathogens, the authors have re-designated them "opportunistic pathogens", but on what grounds? My point was that treating all E. coli as pathogens is not justified on the basis of 16S sequencing. For that we would need to know about lt, eae, ipaH, aat and quite a few other genes. I am as unconfortable, if not more so, about "opportunistic pathogens" as I was about "pathogens". My point was that some E. coli are demonstrably pathogenic, but not all. It would be less imprecise to describe it as "a species which includes many important diarrhoeagenic pathogens".

Minor
2 One row in Table 1 has n-640. How?
3 The 'bullet points' at the beginning of Results should be removed.
4 In line 186, this was not a case/control study but a controlled trial.
5 I could not find a citation in the text to Figure S4
6 The text is confusing about Figure 5 and Figure S12, only one of which has dotted lines in the dendrogram.

Reviewer #2:
Remarks to the Author:
Overall, I find the manuscript much improved and the shifted focus of the analysis and discussion is of more broad interest to the microbiome field. I have just a couple remaining minor points.

1. The below statement is confusing. What is meant by “our”? I assume it means the data presented in this manuscript. Best to reword to be clear. Also, it seems the ratio is reversed (ie should be 10:1 not 1:10)?

"Our high Prevotella-to-Bacteroides ratio (~1:10) also highlights an interesting intestinal dynamic which changes through industrialization and shifts in diet."

2. The final statement of the manuscript is problematic given the prior statements in the discussion. Are opportunistic pathogens more of an issue in industrial, low prevotella microbiome people? I think an argument could be made that the opposite is true, however, it would be difficult to disentangle the effects of abx use and sanitation practices in industrialized regions of the world on opportunistic

pathogens vs loss of prevotella. It seems that an argument could be made (and is made in the prior statements in the discussion) that the prevotella networks could play a role in keeping diseases of affluence at bay. This final statement could be interpreted as saying that Bacteroides are opportunistic pathogens given that the manuscript demonstrates their anti-correlation, which I don't think is what the authors are implying.

"The parallel loss of P. copri and the P. stercorea trophic network, whose development is described here, indicates the likely importance of the metabolic pathways and capabilities of Prevotella and species associated with them, both for maintaining a higher fermentative capacity (SCFA production), a healthier gut microbiome environment and for keeping opportunistic pathogens at bay."

Reviewer #3:
Remarks to the Author:
De Goffau and Jallow et al., have improved the manuscript and taken into consideration of the reviewers comments. However, I would add a couple minor points. Firstly, in response to potential influence of intestinal parasites and gut inflammation, the authors respond to the comment but it would be good if this was noted in the methods when describing the cohort. Secondly, on lines 552-553, The Huttenhower group is credited with the P. copri meta analysis (https://doi.org/10.1016/j.chom.2019.08.018), while true that C. Huttenhower was involved in the study, the main lead on the project should be accredited to N. Segata.

Reviewer #4:
Remarks to the Author:
The authors have recognized that many of the statistics presented initially suffered from bias, such as not accounting for repeated measures. Having addressed these issues the manuscript is now much improved and could be published if the other reviewers are satisfied. I have only one point of clarification:

- Can the authors confirm that whenever they used statistics such as PERMANOVA, that does not account for repeated measures, they only include 1 sample per subject?

Final Decision Letter:

Dear Josef,

I am pleased to accept your Article "Gut microbiomes from Gambian infants reveals the development of a non-industrialised Prevotella-based trophic network" for publication in Nature Microbiology. Thank you for having chosen to submit your work to us and many congratulations.

Before your manuscript is typeset, we will edit the text to ensure it is intelligible to our wide readership and conforms to house style. We look particularly carefully at the titles of all papers to ensure that they are relatively brief and understandable.

Once your manuscript is typeset and you have completed the appropriate grant of rights, you will receive a link to your electronic proof via email with a request to make any corrections within 48 hours. If, when you receive your proof, you cannot meet this deadline, please inform us at rjsproduction@springernature.com immediately. Once your paper has been scheduled for online publication, the Nature press office will be in touch to confirm the details.

Acceptance of your manuscript is conditional on all authors' agreement with our publication policies (see https://www.nature.com/nmicrobiol/editorial-policies). In particular your manuscript must not be published elsewhere and there must be no announcement of the work to any media outlet until the publication date (the day on which it is uploaded onto our website).

Please note that <i>Nature Microbiology</i> is a Transformative Journal (TJ). Authors may publish their research with us through the traditional subscription access route or make their paper immediately open access through payment of an article-processing charge (APC). Authors will not be required to make a final decision about access to their article until it has been accepted. Find out more about Transformative Journals

<B>Authors may need to take specific actions to achieve compliance with funder and institutional open access mandates. For submissions from January 2021, if your research is supported by a funder that requires immediate open access (e.g. according to Plan S principles) then you should select the gold OA route, and we will direct you to the compliant route where possible. For authors selecting the subscription publication route our standard licensing terms will need to be accepted, including our self-archiving policies. Those standard licensing terms will supersede any other terms that the author or any third party may assert apply to any version of the manuscript.

In approximately 10 business days you will receive an email with a link to choose the appropriate publishing options for your paper and our Author Services team will be in touch regarding any additional information that may be required.

You will not receive your proofs until the publishing agreement has been received through our system.

If you have any questions about our publishing options, costs, Open Access requirements, or our legal forms, please contact ASJournals@springernature.com

An online order form for reprints of your paper is available at https://www.nature.com/reprints/author-reprints.html. All co-authors, authors' institutions and authors' funding agencies can order reprints using the form appropriate to their geographical region.

We welcome the submission of potential cover material (including a short caption of around 40 words) related to your manuscript; suggestions should be sent to Nature Microbiology as electronic files (the image should be 300 dpi at 210 x 297 mm in either TIFF or JPEG format). Please note that such

pictures should be selected more for their aesthetic appeal than for their scientific content, and that colour images work better than black and white or grayscale images. Please do not try to design a cover with the Nature Microbiology logo etc., and please do not submit composites of images related to your work. I am sure you will understand that we cannot make any promise as to whether any of your suggestions might be selected for the cover of the journal.

You can now use a single sign-on for all your accounts, view the status of all your manuscript submissions and reviews, access usage statistics for your published articles and download a record of your refereeing activity for the Nature journals.

To assist our authors in disseminating their research to the broader community, our SharedIt initiative provides you with a unique shareable link that will allow anyone (with or without a subscription) to read the published article. Recipients of the link with a subscription will also be able to download and print the PDF.

As soon as your article is published, you will receive an automated email with your shareable link.
